# Antiviral, Cytotoxic, and Antioxidant Activities of Three Edible Agaricomycetes Mushrooms: *Pleurotus columbinus*, *Pleurotus sajor-caju*, and *Agaricus bisporus*

**DOI:** 10.3390/jof7080645

**Published:** 2021-08-08

**Authors:** Shaza M. Elhusseiny, Taghrid S. El-Mahdy, Mohamed F. Awad, Nooran S. Elleboudy, Mohamed M. S. Farag, Khaled M. Aboshanab, Mahmoud A. Yassien

**Affiliations:** 1 Department of Microbiology and Immunology, Faculty of Pharmacy, Ahram Canadian University (ACU), 4th Industrial Area, 6th of October City, Cairo P.O. Box 12566, Egypt; shaza.ahmed@acu.edu.eg (S.M.E.); taghrid-elmahdy@pharm.helwan.edu.eg (T.S.E.-M.); 2 Department of Microbiology and Immunology, Faculty of Pharmacy, Helwan University, Cairo P.O. Box 11795, Egypt; 3 Department of Biology, College of Science, Taif University, P.O. Box 11099, Taif 21944, Saudi Arabia; m.fadl@tu.edu.sa; 4 Department of Microbiology and Immunology, Faculty of Pharmacy, Ain Shams University, Organization of African Unity St., Abbassia, Cairo P.O. Box 11566, Egypt; nooran.elleboudy@pharma.asu.edu.eg (N.S.E.); mahmoud.yassien@pharma.asu.edu.eg (M.A.Y.); 5 Botany and Microbiology Department, Faculty of Science, Al-Azhar University, Nasr City, Cairo P.O. Box 11884, Egypt; mohamed.farag@azhar.edu.eg

**Keywords:** white rot fungus, *Pleurotus columbinus*, *Pleurotus sajor-caju*, *Agaricus bisporus*, medicinal mushrooms, antioxidant, cytotoxic, antiviral

## Abstract

In this study, we investigated aqueous extracts of three edible mushrooms: *Agaricus bisporus* (white button mushroom), *Pleurotus columbinus* (oyster mushroom), and *Pleurotus sajor-caju* (grey oyster mushroom). The extracts were biochemically characterized for total carbohydrate, phenolic, flavonoid, vitamin, and protein contents besides amino acid analysis. Triple TOF proteome analysis showed 30.1% similarity between proteomes of the two *Pleurotus* spp. All three extracts showed promising antiviral activities. While *Pleurotus columbinus* extract showed potent activity against adenovirus (Ad7, selectivity index (SI) = 4.2), *Agaricus bisporus* showed strong activity against herpes simplex II (HSV-2; SI = 3.7). The extracts showed low cytotoxicity against normal human peripheral blood mononuclear cells (PBMCs) and moderate cytotoxicity against prostate (PC3, DU-145); colorectal (Colo-205); cecum carcinoma (LS-513); liver carcinoma (HepG2); cervical cancer (HeLa); breast adenocarcinoma (MDA-MB-231 and MCF-7) as well as leukemia (CCRF-CEM); acute monocytic leukemia (THP1); acute promyelocytic leukemia (NB4); and lymphoma (U937) cell lines. Antioxidant activity was evaluated using 2,2-diphenyl-1-picryl-hydrazyl-hydrate (DPPH) radical scavenging, 2,2′-Azinobis-(3-Ethylbenzthiazolin-6-Sulfonic Acid) ABTS radical cation scavenging, and oxygen radical absorbance capacity (ORAC) assays. The three extracts showed potential antioxidant activities with the maximum activity recorded for *Pleurotus columbinus* (IC_50_ µg/mL) = 35.13 ± 3.27 for DPPH, 13.97 ± 4.91 for ABTS, and 29.42 ± 3.21 for ORAC assays.

## 1. Introduction

Mushrooms have been used in traditional ancient therapies since the Neolithic period. Humankind has valued mushrooms as an edible and medicinal food source. Most of the ancient knowledge about medicinal mushrooms has been confirmed and registered by modern science [1]. Current mushrooms with medicinal value are used in various fields such as dietary food, nutritive auxiliary products, and in the medicinal field called “pharmaceuticals of mushrooms” [2]. Remedial mushrooms are similar to “curing plants” in that they are observable fungi, mostly some Ascomycetes and higher Basidiomycetes, that are used in the extract formula or powder for disease avoidance, allegation or mending, and/or to provide an equitable healthy regimen [2]. About 130 therapeutic actions are thought to exist in medicinal mushroom and fungi, including antitumor, antiviral, antiparasitic, antibacterial, antifungal, antioxidant, radical scavenging, detoxicating, immunomodulating,, antidiabetic, and hepatoprotective effects [3]. Medicinal mushroom are used with patients under chemotherapeutic treatment or radiation therapy, in different forms of cancers, bloodborne chronic viral infections of hepatitis B, C, and D, and herpes simplex virus (HSV) [4].

Many edible mushrooms have been portrayed as therapeutic cures for a range of ailments. Both mycelia and fruiting bodies were used to extract fractions with antiviral activity [5]. The identified active compounds appeared to operate by directly inhibiting viral enzymes, viral replication, absorption of virus, and mammalian cells uptake. Small molecules were responsible for the direct antiviral activity, whereas the indirect effects may be elicited by polysaccharides or complex molecules [6].

Mushroom carbohydrates inhibit tumor genesis, have direct anticancer activities against a variety of synergetic tumors, and stop tumors from spreading. When used in combination with chemotherapy, their operation is particularly beneficial [7]. Polysaccharides’ antitumor activity is resolved through a thymus-dependent immune system, which requires an intact T cell portion [8]. In vitro and in vivo studies indicate that medicinal mushroom drugs besides medicinal mushroom polysaccharide preparations from various mushroom species are effective in treating cancer [9]. Biological reaction modifiers are a new class of antitumor medicinal mushrooms medications (BRMs) [10]. Along with surgery, chemotherapy, and radiotherapy, BRMs have been used as an advanced type of cancer treatment [10]. Moreover, enzyme therapies, particularly those produced by mushrooms, have been shown to be effective in the treatment of cancer by limiting oxidative stress and restricting cell growth [11].

Interestingly, mushrooms are rich in high-quality proteins that contain the essential amino acids, vitamins, carbohydrates, minerals, unsaturated fatty acids, and fibers. They are considered an excellent and nutritious food for people with high blood cholesterol levels or hypertension due to their low energy, fat, and sodium contents [12]. Aside from phenolic, mushrooms with relatively high levels of vitamins A, C, and β-carotene have been shown to be the key contributor to their antioxidant activity [13]. Researchers are interested in quantifying in vitro mushroom extracts antioxidant activities as a way to easily evaluate total antioxidant concentrations and possible effectiveness due to the potential benefits of antioxidant intake [14]. In addition, Barros et al. reported that flavonoids of mushroom can play an important role as free radical scavengers, stopping the chain reactions that occur during triglyceride oxidation in the food system [15]. Therefore, this study was conducted to assess the antioxidant, cytotoxic, and antiviral activities of the three edible mushroom aqueous extracts, namely *Pleurotus columbinus*, *Pleurotus sajor-caju*, and *Agaricus bisporus* followed by composition analysis of the respective extracts.

## 2. Materials and Methods

### 2.1. Collection and Culture Conditions of Fungal Isolates

**Figure 1:**
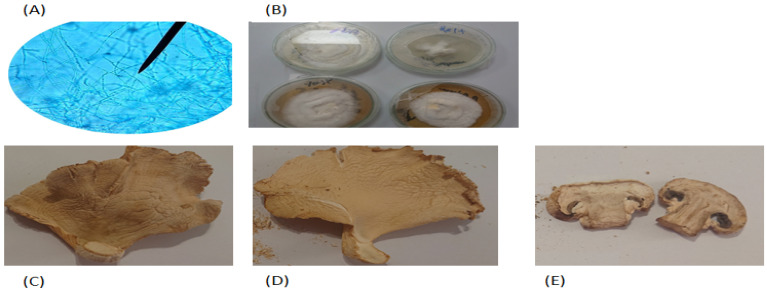
(**A**) Microscopic examination of the white rot fungus using light microscope, (**B**) growth of mushroom on potato dextrose agar post 7 days culture. Dried fruiting bodies of the collected mushroom isolates, (**C**) *Pleurotus columbinus*, (**D**) *Pleurotus sajor-caju*, and (**E**) *Agaricus bisporus*.

Preidentified *Pleurotus columbinus, Pleurotus sajor-caju*, and *Agaricus bisporus* mushrooms were obtained, as spawns and/or fruiting bodies (Figure 1), from the culture collection of Al-Orman botanical reservoir, Cairo, Egypt [16,17,18]. For subculturing from fungal fruits, sterile surgical blades were aseptically used to cut the fruit. The inner mycelia were picked by sterile needles and subcultured in sterile potato dextrose agar (PDA; Merck, Darmstadt, Germany) plates [19]. For subculturing of fungal spawns, sterile forceps were used to pick the seeds and aseptically transfer them to sterile PDA plates that were incubated at 28 °C for 7 days, and then the fruiting bodies of each isolate were separately washed. Aliquots (500 g) of each isolate were air dried at room temperature for 48 h, ground in a kitchen grinder and stored at room temperature in a well-aerated place until use. 

### 2.2. Molecular Identification of Mushroom Species

#### 2.2.1. Extraction of Genomic DNA and PCR Amplification

Total genomic DNA was extracted from 7-day-old mycelial mats using Zymo Research (ZR) Fungal/Bacterial DNA kit™ (Zymo Research, Irvine, CA, USA) following the manufacturer instructions.

The internal transcribed spacer (ITS) region of the rDNA was amplified by PCR with previously described universal primers ITS1 (5′-TCC GTA GGT GAA CCT GCG G-3′) and ITS4 (5′-TCC TCC GCT TAT TGA TAT GC-3′) [20]. PCR reaction mixture was performed in a total volume of 50 μL containing 8 μL DNA, 1 μL (20 pico mol) of each primer, 25 μL My *Taq* Red mixture (Bioline GmbH, Luckenwalde, Germany) and 15 μL nuclease free water. The amplification reaction was done in a C1000 Touch thermal cycler (BioRad, Hercules, CA, USA) according to Das et al. [21] with slight modifications. Initial denaturation at 94 °C for 6 min, followed by 35 cycles of denaturation at 94 °C for 45 s, annealing at 56 °C for 45 s, extension at 72 °C for 1 min, and a final extension at 72 °C for 5 min.

#### 2.2.2. DNA Sequencing and ITS Sequence Analysis 

The pair of universal ITS primers ITS-1 (forward) and ITS-4 (reverse) was used for the Sanger sequencing of the purified PCR products using model ABI PRISM^®^ 3500XL DNA Sequencer (Applied Biosystems, Foster City, CA, USA) following manufacturer’s instructions. For good quality sequence assurance, FinchTV version1.4.0 software was used for the analysis of sequences (sense and antisense) resulting from sequencing reaction (Geospiza, Inc.; Seattle, WA, USA; https://digitalworldbiology.com/FinchTV, http://www.geospiza.com (accessed on 5 August 2021)). The resulting sequences were edited using BioEdit Sequence Alignment Editor (Ibis Therapeutics, Carlsbad, CA, USA). Then, the resulting consensus ITS sequences were blasted in the NCBI (http://www.ncbi.nlm.nih.gov, accessed on 5 August 2021) database with the BLASTn for homology in order to identify the probable mushroom in question. The sequences were deposited in the GenBank^®^. 

### 2.3. Mushroom Aqueous Extract Preparation

The dried fruit of each isolate was blended using a household blender to give 500 g powder, then macerated three times for three successive days in 7 L of distilled water at room temperature and filtered. Maceration and filtration were repeated until exhaustion. The yield was stored with ethanol. A total of 500 mL of each isolate was freeze-dried after ethanol evaporation at 45 °C to give approximately 20 g dry residue of each extract [22].

### 2.4. Biochemical Charactrization of the Crude Mushroom Extracts

#### 2.4.1. Total Carbohydrate Content 

Total soluble carbohydrate content was assayed using phenol sulfuric acid method as previously reported [23]. Data are presented as means ± standard deviation (SD) and experiments were performed in triplicate. 

#### 2.4.2. Total Phenolic and Total Falvonoid Content

Phenolic and flavonoid content was determined as reported by Ryan et al. [24]. Samples were prepared at a concentration of 20 mg/mL in water. Gallic acid (1 mg/mL stock solution) was prepared in methanol, and 9 serial dilutions were prepared (concentrations: 12.5, 25, 50, 100, 200, 400, 500, 800, and 1000 µg/mL) for the calibration curve. Rutin (1 mg/mL stock solution) was prepared in methanol, and 10 serial dilutions were prepared (concentrations 1000, 800, 500, 400, 200, 100, 50, 25, 12.5, and 6.25 µg/mL) used in the calibration curve. Gallic acid standards and samples were pipetted in the wells of the plate in six replicates and measured at 630 nm. Each of the 10 rutin standards and samples in six replicates were measured at 420 nm.

#### 2.4.3. Analysis of Phenolics and Flavonoids Using High-Performance Liquid Chromatography (HPLC)

This was performed according to the method of Singh et al. [25]. Each of the samples and 10 different standards solutions were dissolved in methanol and filtered using 0.22 µm syringe filters; then, 100 µL of each sample and 10 µL of each standard were injected using HPLC column, Waters 2690 Alliance HPLC system equipped with a Waters 996 photodiode array detector. Column C18 Inertsil ODS 3: 4.6 × 250 mm, 5 µm, and mobile phase: Buffer (0.1% phosphoric acid in water) and methanol mode of elution was gradient with a flow rate of 1 mL/min at wavelength 280 nm.

#### 2.4.4. Vitamin Content

##### Water Soluble Vitamins 

The three tested mushroom extracts (50 mg/mL), and reference standards (10 mg in 10 mL 0.05 M sodium hydroxide) of each of the seven water soluble vitamins (thiamine HCl, ascorbic acid, riboflavin, nicotinic acid, nicotinamide, pyridoxine HCl, and folic acid) were diluted to 100 µg/mL and filtered using a 0.22 µm syringe filter; then, 100 µL were injected onto the HPLC column, Waters 2690 Alliance HPLC system (Milford, CT, USA) equipped with a Waters 996 photodiode array detector. Column Inertsil ODS 3: 4.6 × 250 mm, 5 µm, mobile phase: Buffer (0.85 g hexane sulphonic acid sodium salt in 1000 mL water and pH was adjusted to 3 with orthophosphoric acid): Methanol elution was gradient at a flow rate of 1 mL/min at wavelength 210 nm [26]. 

##### Fat Soluble Vitamins 

Solutions (50 mg/mL) of the three mushroom extracts and a standard solution in methanol of three fat vitamins; E, D3, and A, at 806.2, 114, and 400 IU/mL, respectively, were filtered using 0.22 µm syringe filters; then, 10 µL of each was injected onto HPLC column, Waters 2690 Alliance HPLC system equipped with photodiode array detector. Column Inertsil ODS 3: 4.6 × 250 mm, 5 µ, mobile phase: Methanol 100% with isocratic elution of flow rate 1 mL/min at wavelength: 210 nm [27]. 

#### 2.4.5. Total Protein Analysis 

This was performed using the bicinchoninic acid (BCA) assay [28] using bovine serum albumin as a standard. One mg of each sample was dispersed in 1 mL phosphate buffer saline (pH 7.4), sonicated for 30 min to extract proteins among other water-soluble substances, filtrated using a 0.45 µm syringe filter, then tested using the BCA Protein Assay Kit (Novagen, Madison, WI, USA) as stated by the manufacturer’s instructions. 

#### 2.4.6. Amino Acids Analysis 

Composition of amino acid of the mushroom extracts was performed using the Sykam amino acid analyzer (Sykam GmbH, Eresing, Germany) equipped with solvent delivery system S 2100 (Quaternary pump with flow range 0.01 to 10.00 mL/min and maximum pressure up to 400 bar), auto sampler S5200, amino acid reaction module S4300 (with built-in dual filter photometer at 570 nm), and refrigerated reagent organizer S4130. One g of each mushroom extract was mixed with 5 ml hexane, allowed to macerate for 24 h, and then the mixture was filtered using Whatman no. 1 filter paper. The residue was placed in a test tube containing 10 mL 6 M HCl and incubated in an oven at 110 °C for 24 h. After the incubation, filtration was done using Whatman no. 1 filter paper, followed by evaporation in a rotary evaporator. The residue was dissolved completely in 2 mL of dilution buffer (Tri-sodium citrate dehydrate 0.06 M; citric acid 0.03 M, phenol 0.02 M, Thiodiglycol 1.4%, HCl 32%), diluted 1000 folds in the same buffer, then loaded onto an ammonia filter column (LCA, K04/Na, 4.6 × 100 mm, Sykam, GmbH, Eresing, Germany) equipped with an automatic amino acid analyzer (Sykam GmbH, Eresing, Germany) [29].

### 2.5. TripleTOF Analysis of Proteomes 

Proteomic analysis was conducted at the proteomics and metabolomics unit at Children’s Cancer Hospital Egypt 57,357 (CCHE), Cairo, Egypt. About 600 μL 8 M urea in 500 mM Tris (pH 8.5) and 60 μL complete ultraproteases (Roche, Mannheim, Germany) were added to each sample; then, samples were shaken vigorously and centrifuged at 10,000 RPM for 30 min. Supernatants were collected and fractions (1 μg/10 μL) were injected using the NanoLC system. The mass spectrometry triple TOF system (Sciex TripleTOF 5600+, AB SCIEX, Concord) was coupled with liquid chromatography (LC) (3 µm, ChromXP C18CL, 120A, 150 × 0.3 mm), consisting of the Eksigent nanoLC400 auto sampler attached to an Ekspert nanoLC425 pump with a flow rate of 10 µL/min for 55 min for each sample [30]. Data analysis was performed by Protein pilot (version 5.0.1.0, 4895) and paragon Algorithm (version 5.0.1.0, 4874). Protein sequences were aligned against sequences in the Swiss-Prot and TrEMBL databases (*Pleurotus* sp. containing 14,792 entries for *Pleurotus columbinus* and *Pleurotus sajor-caju*, and *Agaricus* sp. containing 29,258 entries for *Agricus bisporus*) [31]. Commonly identified proteins between the mushroom species were illustrated using Venny2.1.0.BioinfoGP software [32] (Kingston upon Hull, UK).

### 2.6. Cell Lines and Cultures 

Primary peripheral blood mononuclear cells (PBMCs), normal, human (ATCC^®^ PCS-800-011™) were purchased from Vascera, Cairo, Egypt. The cancer cell lines human leukemia (CCRF-CEM); acute promyelocytic leukemia (NB-4); human lymphoma (U937); prostate cancer (DU-145 and PC3); hepatocellular carcinoma (HepG2; breast adenocarcinoma (MCF-7 and MDA-MB-231); Vero; and Hep-2 cells were obtained from Nawah Scientific Inc., (Mokatam, Cairo, Egypt). Cervical cancer (HeLa), acute monocytic leukemia (Thp1), colorectal carcinoma (Colo-205); and cecum carcinoma (LS-513) cells were maintained in Roswell Park Memorial Institute (RPMI) medium (Lonza, Germany) supplemented with 100 mg/mL of streptomycin, 100 units/mL of penicillin, and 10% of heat-inactivated fetal bovine serum. For other cell lines, Dulbecco’s modified Eagle’s medium (DMEM; Invitrogen, Carlsbad, CA, USA) supplemented with 10% FBS (Hyclone, Marlborough, MA, USA), 10 µg/mL of insulin (Sigma-Aldrich, St. Louis, MO, USA), 100 mg/mL of streptomycin, and 100 U/mL of penicillin (Grand Island, NY, USA) was used for cell culture. Incubation was performed at 37 °C in a humidified atmosphere with 5% CO_2_. 

### 2.7. Antiviral Activity 

Antiviral activity was tested against adenovirus type 7 (Ad7) and herpes simplex virus type 2 (HSV-2) (Nawah Scientific, Mokattam, Egypt). Antiviral activity was assessed as previously described [33]. Vero and Hep-2 cells were seeded into a 96-well culture plate at a density of 2 × 10^4^ cells/well one day before infection. The cell culture medium (2.5) was removed the next day, and phosphate-buffered saline (PBS) was used for cells to be washed. The infectivity of human Ad7 and HSV2 was determined using the sulforhodamine B (SRB) method [34], which monitored CPE and calculated the percentage of cell viability. One hundred microliters of diluted virus suspension of Ad7 and HSV-2 containing 50% cell culture infective dose (CCID_50_) of virus stock was added to mammalian cells. To test the antiviral activity of mushroom extracts, 0.01 mL of medium containing each extract (10-fold serial dilution ranging from 0.1 to 100 µg/mL) was added to cells. Controls (virus-infected, non-extract-treated cells and noninfected, non-extract-treated cells) were included. After 4 days of incubation at 37 °C in 5% CO_2_, PBS was used to wash cells, then 0.01 mL of 70% (*v*/*v*) cold acetone was added and left for 30 min at −20 °C. After removing acetone, plates were dried at 60 °C for 30 min, then wells were filled with 0.01 mL of 0.4% (*w*/*v*) SRB solution in 1% acetic acid (*v*/*v*) and at room temperature incubated for 30 min. Unbound SRB was removed from the plates by washing 5 times using 1% acetic acid (*v*/*v*) and allowing them to dry. Fixed SRB in wells was dissolved in 100 µL of unbuffered Tris base solution (10 mM), and then plates were incubated for 30 min at room temperature. Finally, optical density (OD) was measured at 540 nm and a reference absorbance of 620 nm using a microplate reader (FluoStar Omega, BMG labtech, Ortenberg, Germany).

To assess cytotoxicity, cells were seeded at a density of 2 × 10^4^ cells/well in a 96-well culture plate. The next day, the cells were given culture medium containing serially diluted samples, which was cultured for 48 h before being withdrawn and the cells rinsed with PBS. The following step was carried out in the same manner as described above for the antiviral activity assay [35].The antiviral activity was calculated based on the extracts ability to inhibit the viral cytopathogenic effects where the 50% cytotoxic concentrations (CC_50_) and the 50% inhibitory concentration (IC_50_) were determined using GraphPad PRISM software (GraphPad Software, San Diego, CA, USA). Selectivity index (SI) was calculated as stated by Doğan et al. [36] according to the Equation:

Selectivity index (SI) = CC_50_/IC_50_ (1)

### 2.8. Evaluation of Cytotoxic Effect against PBMCs

Cytotoxicity was assessed using MTT assay [37]. Aliquots (100 µL) of cell suspensions (cell density 1.2–1.8 × 10^4^ cells/well) were placed in 96-well plates, complete medium (2.5) was added, and cells were incubated for 24 h. Then cells were treated with serial dilutions of fungal extracts for 48 h. MTT (3-(4, 5-dimethylthiazol-2-yl)-2, 5-diphenyltetrazolium bromide, 5 mg/mL in PBS) was added to wells and incubated for 4 h. Formazan crystals were extracted using MTT solubilization solution (10% Triton X-100 plus, 0.1 M HCl in anhydrous isopropanol) and at 570 nm absorbance were read using microplate reader.

### 2.9. Evaluation of Cytotoxic Effect against Cancer Cell Lines

Cytotoxicity against prostate cancer (DU-145 and PC3); hepatocellular carcinoma (HepG2); colorectal carcinoma (Colo-205); cecum carcinoma (LS-513); cervical cancer (HeLa); and breast adenocarcinoma (MDA-MB-231 and MCF-7) cell lines was evaluated using a sulforhodamine B (SRB) colorimetric assay. Cell suspensions in complete media (2.5) were incubated in 96-well plates for 24 h. Then, various concentrations (0 to 100 μg/mL in complete media) of mushroom extracts were added and incubated for 72 h. Cell viability was measured using SRB method as described above (2.6).

### 2.10. Cytotoxcicty Assay against Leukemia and Lymphoma Cell Lines

Cytotoxicity against human leukemia (CCRF-CEM), acute promyelocytic leukemia (NB4), acute monocytic leukemia (THP1), and human lymphoma (U937) was determined using the Abcam^®^ Water Soluble Tetrazolium Salts (WST-1) assay kit (ab155902 WST-1 Cell Proliferation Reagent, UK). Cell suspensions were incubated for 24 h, and then treated with different concentrations (0 to 100 μg/mL in complete media) of mushroom extracts for 48 h. Cells were then treated with 10 μL WST-1 reagent for 1 h, then A_450_ was read using a microplate reader [38].

### 2.11. Antioxidant Activity Assay 

#### 2.11.1. DPPH Radical Scavenging Activity 

DPPH radical scavenging assay was carried out according to the method of [39]. Briefly, in 96-well plates, 100 µL of freshly prepared DPPH reagent (Sigma-Aldrich, Taufkirchen, Germany) (0.1% in methanol) were added to 100 µL of a range of concentrations (0 to 100 μg/mL) of the mushroom extracts, in 6 replicates each, and incubated in the dark at room temperature for 30 min. The resulting reduction in DPPH color was measured at 540 nm. Antioxidant activity was expressed as the inhibition percentage with reference to calibration curve (*R*^2^ = 0.9903). Data are represented as means ± SD according to Equation (2):

*Percentage inhibition%* = (*Average absorbance of blank − average absorbance of the* extract) × 100 *Average absorbance of blank* (2)

#### 2.11.2. ABTS Radical Cation Scavenging Activity 

The assay was carried out as previously stated [40]. Mushroom extract (10 µL of 0 to 100 μg/mL solutions) were mixed with 190 µL of freshly prepared ABTS (Sigma-Aldrich, Taufkirchen, Germany) in 96-well plates, incubated in the dark at room temperature for 2 h. Each sample was tested in 4 replicates and absorbance was measured at 734 nm. Antioxidant activity was expressed as percentage of inhibition (Equation (1)) with reference to calibration curve (*R*^2^ = 0.9948).

#### 2.11.3. Oxygen Radical Absorbance Capacity (ORAC) Assay

The analysis was carried out as previously determined [41], with minor modifications. Briefly, 12.5 µL of the mushroom extracts, in triplicate, were incubated with 75 µL of fluorescein (10 nM) for 30 min at 37 °C. Fluorescence measurement (485 EX, 520 EM, nm) was carried out for three cycles (cycle time = 90 s). Afterwards, 12.5 µL of freshly prepared 240 mM solution of 2,2′-Azobis 2-amidinopropane dihydrochloride (AAPH) (Abcam, Cambridge, UK) were added to each well immediately and fluorescence measurement was continued for 2.5 h (85 cycles, each 90 sec). Antioxidant activity was expressed as percentage of inhibition (Equation (1)) with reference to calibration curve (*R*^2^ = 0.9957).

### 2.12. Statistical Analysis

For all experiments, results were presented as the mean ± standard deviation (SD) of, unless otherwise specified, three independent readings. Statistical analyses were performed using one-way ANOVA. The significance of differences between means was evaluated with the Tukey–Kramer multiple comparisons test. *p* ≤ 0.05 was considered statistically significant. Statistical evaluation was determined using GraphPad PRISM software (Graph-Pad Software, San Diego, CA, USA). 

## 3. Results

### 3.1. Molecular Identification of Mushrooms

According to BLASTn results the mushrooms were identified as *Pleurotus columbinus*, *Pleurotus sajor-caju* (*Lentinus sajor-caju*), and *Agaricus bisporus*, and the sequences were deposited in GenBank^®^ with accession numbers MZ642245, MZ642259, and MZ642282, respectively.

### 3.2. Biochemical Characterization of Mushroom Extract

The biochemical analysis (total carbohydrate, protein, phenolic, and flavonoid contents) showed distinctive differences between the three mushrooms (Table 1). *Pleurotus columbinus* extract had the highest carbohydrate content and the lowest phenolic content. It is worth noting that flavonoids were not detected in the *Pleurotus columbinus* extract; neither were carbohydrates in the *Pleurotus sajor-caju* extract.

#### 3.2.1. Characterization of Total Phenolic and Total Falvonoid Contents by HPLC 

Characterization of phenols and flavonoids in the three mushroom extracts was done by HPLC. The chromatogram is shown in Figure S1 and the TPC and TFC quantification is in Table 2. Catechin was found in all 3 mushroom extracts (retention time 26.48 min), cholorgenic acid was detected in *Pleurotus sajor-caju* and *Agaricus bisporus* extracts at retention time 29.61 min, and gallic acid was found in *Pleurotus columbinus* and *Pleurotus sajor-caju* extracts at retention time 12.22 min. Caffeic acid, hesperidin, rutin, ellagic acid, kampeferol, quercetin, and apigenin were not detected in any of the mushroom extracts. There results were confirmed by including a mixture of standard phenolics and flavonoids that has shown the same retention times as those of the detected in the mushroom extracts.

#### 3.2.2. Vitamin Content 

The content of both water-soluble and fat-soluble vitamins was analyzed using HPLC (chromatograms in Figures S2 and S3). Vitamin C (at a high content), nicotinic acid, nictinamide, and vitamin D were detected in all three mushrooms, unlike folic acid, thiamine, vitamin A, and vitamin E (Table 3). Riboflavin was only detected in *Agaricus bisporus* (0.11 µg/100 g). 

#### 3.2.3. Amino Acids Analysis 

The composition of amino acids of the mushroom extracts as detected using the Sykam Amino Acid Analyzer is shown in Table 4 and Figure S4. A wide range of amino acids was detected in the mushroom extracts, with the exceptions of histidine and aspartic acid, which were not detected. It is worth noting that glutamic acid was the major amino acid in all three mushroom extracts. 

#### 3.2.4. Triple TOF Analysis of Proteomes

**Figure 2:**
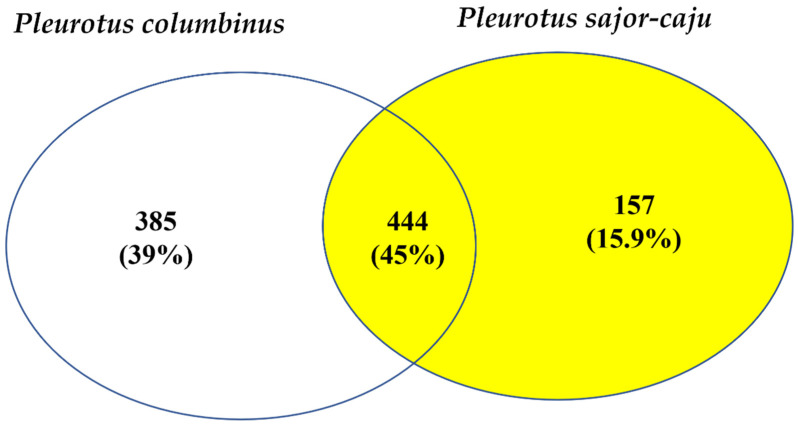
Venn diagram showing the common identified proteins between the three mushroom species *Pleurotus columbinus*, *Pleurotus sajor-caju*, and *Agaricus bisporus*.

Protein and peptide identification and quantification of the three mushroom extracts were performed using triple TOF. A total of 541 proteins were identified in *Pleurotus columbinus*, 402 in *Pleurotus sajor-caju,* and 408 in *Agaricus bisporus* (Tables S1–S3, respectively). The Venn diagram in Figure 2 summarizes the common proteins between the two tested *Pleurotus* species. Whereas 444 proteins with the same accession codes were found in both *Pleurotus columbinus* and *Pleurotus sajor-caju*, no proteins with the same accession codes were found in common between the two *Pleurotus* mushrooms and *Agaricus bisporus.* A multitude of proteins and enzymes were detected, including S-adenosyl-L-homocysteine hydrolase, intradiol dioxygenase, alpha- and beta-form tubulin, thioredoxin reductase, haloacid dyhydrogenase, serine proteinase, ubiquitin-like protein, prohibitin, tubulin, septin, and chaperone. Pleurotolysin and lectin were also identified in both *Pleurotus* species.

### 3.3. Cytopatheic Effect against Viral Cell Lines 

The antiviral activities of the aqueous extracts *of Pleurotus columbinus*, *Pleurotus sajor-caju*, and *Agaricus bisporus* were examined in vitro against human adenovirus type 7 and herpes simplex virus type II. The dose–response curves are shown in Figures S5 and S6, and the CC_50_, IC_50,_ and selectivity indices (SI) in Table 5. Potent antiviral activities with relatively low toxicities were recorded for *Pleurotus columbinus* against Ad7 and *Agaricus bisporus* against HSV2.

### 3.4. Cytotoxic Activity against Normal Human PBMCs

All three mushroom extracts (*Pleurotus columbinus*, *Pleurotus sajor-caju*, and *Agaricus bisporus*) showed low cytotoxicity against the normal human PBMCs (IC_50_ µg/mL; 75.03 ± 0.62, 70.42 ± 1.89, and 57.72 ± 0.95, respectively). 

#### 3.4.1. Cytotoxic Activity against Cancer Cell Lines 

**Figure 3:**
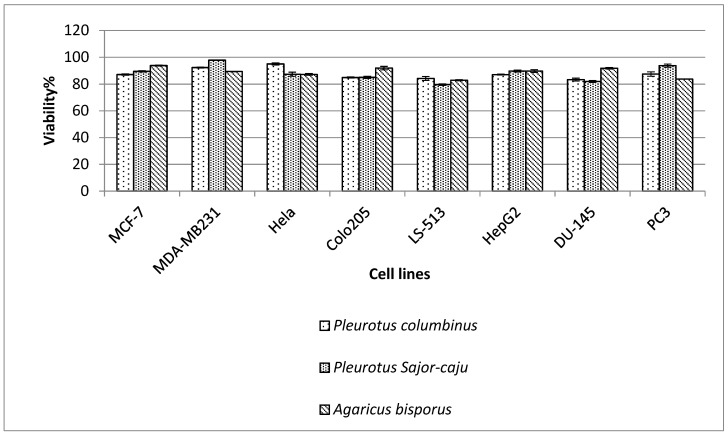
Cytotoxic effect of the three mushrooms extracts *Pleurotus columbinus*, *Pleurotus sajor*-*caju*, and *Agaricus bisporus* against cancer cell lines MCF-7, MDA-MBA-231, Hela, Colo-205, LS-513, HepG2, Du-145, and PC-3.

Cytotoxicity of the three mushroom extracts was tested at five different concentrations (0.01, 0.1, 1, 10, and 100 µM) on a range of cancer cell lines including prostate cancer (DU-145 and PC3); hepatocellular carcinoma (HepG2); colorectal carcinoma (Colo-205); cecum carcinoma (LS-513); cervical cancer (HeLa); and breast adenocarcinoma (MDA-MB-231 and MCF-7). Results are shown in Figure 3. *Pleurotus sajor-caju* and *Agaricus bisporus* reported the highest cytotoxicity against LS-513 cell line with cell viabilities 79.60 ± 0.53% and 82.83 ± 0.42%, respectively.

#### 3.4.2. Cytotoxic Activity against Leukemia and Lymphoma 

**Figure 4:**
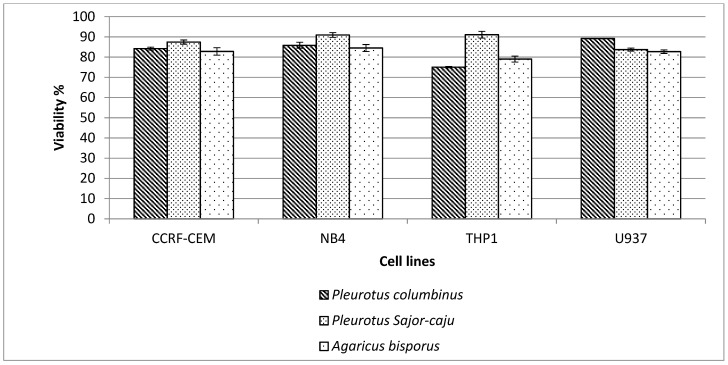
Cell viability of the three mushrooms extracts *Pleurotus columbinus*, *Pleurotus sajor-caju*, and *Agaricus bisporus* against leukemic cells (CCRF-CEM, NB4, and THP1) and lymphoma U937.

Mushroom extracts of *Pleurotus columbinus*, *Pleurotus sajor-caju*, and *Agaricus bisporus* were tested for cytotoxicity against leukemia and lymphoma cells at a concentration of 100 µM. As shown in Figure 4, comparable results were obtained for the three mushroom extracts. 

### 3.5. Antioxidant Activity

Antioxidant activity was investigated through three different evaluation methods (Table 6) against the antioxidant standard, Trolox. The DPPH assay showed that the free radicle scavenging ability of *Pleurotus columbinus* was significantly higher than *Pleurotus sajor-caju* and much higher than that of *Agaricus bisporus*. The ABTS scavenging activity showed that all three extracts showed antioxidant activities higher than that of Trolox. Results of the ORAC assay are shown in Table 6, Figures S7a,b and S8 with results of various concentrations of Trolox. ORAC assay results show that *Pleurotus columbinus* and *Pleurotus sajor-caju* have significantly higher antioxidant capacities than *Agaricus bisporus*. 

## 4. Discussion

The three mushrooms *Pleurotus columbinus*, *Pleurotus sajor-caju*, and *Agaricus bisporus* are edible mushrooms with high consumption in many countries [42]. In this study, we biochemically characterized the aqueous extracts of the respective three mushrooms. Our results revealed that they were all protein-rich and that *Pleurotus sajor-caju* contained the least carbohydrate content [43]. Similar data were reported by Agarwal et al. (2017), who recommended *Pleurotus sajor-caju* for diabetics and weight watchers [44]. It is well documented that phenolics are ubiquitous in mushrooms at approximately 2 to more than 30 mg/g concentration for *Pleurotus* spp. [45,46]. Our data reported phenolic concentrations of 22.50 ± 1.53 and 18.16 ± 0.54 for *P. columbinus* and *P. sajor-caju*, respectively. The phenol content of *Agaricus bisporus* was 27.45 ± 0.8 mg/g. Gąsecka et al. [47] studied the properties of seven *Agaricus* spp. mushrooms and reported a wide range of total phenolic content (132.7 to 1154.7 mg GAE/100 g DW). The presence of flavonoids in *Pleurotus* spp. is reported to be species specific [45,48]. In this study, flavonoids were not detected in *Pleurotus columbinus*. Similarly, Vieira et al. [49] reported the absence of flavonoids in *P. ostreatus*. The highest flavonoid concentration was found in *A. campestris* (15.63 mg GAE/g extract). Catechin was detected in the three mushrooms. Likewise, Butkhup et al. [14] reported the presence of catechin in 25 edible mushrooms. Catechin has often been linked to antioxidant activity of natural extracts [50,51,52].

Since all three mushroom species belong to Agaricomycetes, their amino acids profiles were remarkably similar. While overall amino acid contents were similar in the three mushrooms (5.04%, 5.78%, and 5.58% for *Pleurotus columbinus, Pleurotus sajor-caju*, and *Agaricus bisporus*, respectively), specific amino acids were found at different concentrations. Glutamic acid was the most prominent amino acid in the three mushroom extracts. Tagkouli et al. [53] also reported that glutamic acid was the most abundant amino acid in the studied three *Pleurotus* extracts.

The vitamin profiles of the three mushrooms were relatively close; this may be because they belong to the same family. Regarding water soluble vitamins, vitamin C was detected in high amounts (1.40, 0.75, and 0.96 mg/100 g in *Pleurotus columbinus*, *Pleurotus sajor-caju*, and *Agaricus bisporus*, respectively) compared to other vitamins. Sánchez et al. [54] reported that mushrooms contain vitamin C at concentrations in the range of 0.15–0.06 mg/mL. Several mushrooms have also been shown to contain vitamin C including *Boletus edulis* [55], *B. pseudosulphureus* [56], *Lactarius deliciosus* [57], *Pleurotus ostreatus* [58], and *Suillus luteus* [59]. Vitamin D was the only fat-soluble vitamin detected in the extracts.

*Pleurotus columbinus*, *Pleurotus sajor-caju*, and *Agaricus bisporus* showed effective antiviral activities against the Adv7 and HSV2. Selectivity index (SI) is commonly used parameters for measuring how safe it is to use a compound as an antiviral agent. SI estimates the gap between the cytotoxic and antiviral activity. Higher SI values indicate higher efficacy and safety of drug use. A perfect drug would affect the virus at low concentration and the cells at a high one; thus, it would eradicate the virus at a concentration that will not harm host cells [60,61].

The antiviral properties of edible mushrooms have been attributed to water extracts and are typically linked to the presence of water-soluble polysaccharides. β glucans isolated from the *Pleurotus tuber-regium* sclerotium have antiviral action against HSV-1 [62]. Huang et al. [63] discovered that an acidic polysaccharide bound to protein isolated from water-soluble extracts of *Ganoderma lucidum* fruiting bodies has ant herpetic action. Sulphates of *Lentinus edodes* (lentinan) β-(1-3)-D-glucan have also been shown to have significant anti-HSV-1 action. Antiviral activity may also be attributed to other molecules. Two phenolic compounds, for example, were extracted from the fruiting bodies of *Inonotus hispidus* and found to have exceptional antiviral activity in face of influenza viruses. [64]

Mushroom components such as peptide RC28, polysaccharide, proteoglycan, sulfated polysaccharide, and triterpenoid (lucialdehyde B, ganoderone A, and ganodermadiol) have exhibited pre- and post-treatment antiviral effects [65]. They could work on all stages of viral replication. The activity of the peptide RC28 against herpes viruses was comparable to the activity of ganciclovir [66]. Peptide RC28 and sulfated polysaccharide from mushrooms have antiviral activity against HSV in vitro as well as in vivo, suggesting that they could be used as therapeutic treatments [66].

The aqueous extracts of the mushrooms showed inhibiting growth effects on the cell lines prostate cancer (DU-145 and PC3); hepatocellular carcinoma (HepG2); colorectal carcinoma (Colo-205); cecum carcinoma (LS-513); cervical cancer (HeLa); and breast adenocarcinoma (MCF-7 and MDA-MB-231) at concentration 100 μg/mL. *Pleurotus columbinus* and *Pleurotus sajor-caju* demonstrated a decrease in the cell viability of MCF-7, Hela, Colo-205, LS-513, HepG2, Du-145, PC-3 and a slight reduction in viability of MDA-MD-231 cells. *Agaricus bisporus* showed the same effect as the other two mushroom isolates but with a little more reduction in the cell viability of MDA-MD-231. *Agaricus bisporus* conjugated linoleic acids have been proved to constrain prostate cancer cell types in vitro [67]. In a similar way, Adams et al. [68] reported that this extract suppresses breast cancer cell proliferation, and drinking white button mushroom powder with green tea may help to prevent breast cancer. Abdalla et al. [69] proposed that *Agaricus blazei* extracts suppressed breast cancer cell proliferation by inhibiting aromatase activity. Gu and Leonard discovered that edible *mushrooms Coprinellus* sp., *Coprinus comatus*, and *Flammulina velutipes* had strong antiproliferative activity in human breast cancer cell lines (MCF-7) and (MDA-MB-231) [70]. 

Though the antiproliferative effect of polysaccharides on tumor cell lines in vitro is unknown, certain studies have shown that incubating polysaccharides with tumor cells can cause changes in signal expression inside the tumor cells. Such modifications could cause cell cycle arrest and apoptosis, which would explain polysaccharides’ antiproliferative activity in vitro [71]. *Inonotus obliquus* mushroom water extract reported to have antitumor activity towards colon cancer cells HT-29. The stimulation of apoptosis, which causes cancer cells to die, was used to restrict cell growth. The mushroom *Inonotu obliquus* has been shown to have anticancer properties in several research studies. Sclerotia extracts from *Inonotu obliquus*, for example, have been shown to decrease tumor cell proliferation and protein synthesis [72]. Suillin, a tetraprenylphenol derivative separated from *Suillus placidus*, Suillin was reported to destroy human liver cancer cells preferentially. Suillin could cause apoptotic death in HepG2 cells in addition to inhibiting proliferation [73]. Lau et al. [74] found that an ethanol–water extract of *Coriolus versicolor*, a commonly used Chinese medicinal mushroom, may dramatically reduce the development of human promyelocytic leukemia in its natural form. HL-60 and NB-4 cells, as well as Raji cells from B-cell lymphoma, were tested in vitro using the MTT assay. The present study found out that the three mushrooms have some cytotoxic effect on the leukemia and lymphoma cell lines. *Pleurotus columbinus*, *Pleurotus sajor-caju*, and *Agaricus bisporus* declared some decline in cell viability of the CCRF-CEM, NB-4, THP1, and human lymphoma (U937). However, *Pleurotus sajor-caju* showed no significant effect against NB4 and THP1 cell lines.

In this study, the protein expression profiles of fruiting bodies were investigated using Triple TOF protein identification. A myriad of proteins and enzymes were detected including catalase, S-adenosyl-L-homocysteine hydrolase, intradiol dioxygenase, alpha- and beta-form tubulin, thioredoxin reductase, haloacid dyhydrogenase, prohibitin, septin, chaperone, serine proteinase, and ubiquitin-like protein. Tubulin was reported to contribute to the cytotoxic activity of mushroom extracts [75,76,77]. Similarly, thioredoxin reductase has shown high cytotoxic activity against a range of cell lines vis-à-vis low systemic toxicity [78,79,80]. Pleurotolysin was detected in the *Pleurotus* species. It is a sphingomyelin-specific cytolysin composed of two subunits (17 and 59 kDa) that integrate to cause an outflow of K ions leading to selective lysis of cancer cells [81,82]. Serine proteinase plays an important role in the antiviral properties of mushrooms [83]. Wang et al. proposed that the anti-HIV effect of the aqueous extracts of some mushroom species may be caused by inhibition of reverse transcriptase enzyme by ubiquitin-like protein [84]. The antioxidant properties of mushroom extracts was attributed to the presence of enzymes including superoxide dismutase, catalase, and glutathione peroxidase, which quench free radicals and detoxify reactive oxygen species [85]. 

The antioxidant activity of the mushroom extracts was investigated using three different activity measurements. This is because antioxidants have multiple mechanisms of action, and no single approach can capture all of them. The two major mechanisms of antioxidant activity are HAT (hydrogen atom transfer) and SET (single electron transfer). whereas DPPH is classified as SET, ABTS, and ORAC assays are HAT reactions [86]. The measurements of the ABTS test showed slightly lower scavenging capacity compared to the DPPH method. The IC_50_ values of the ABTS scavenging method for *Pleurotus columbinus*, *Pleurotus sajor-caju*, and *Agaricus bisporus* were 13.97 ± 4.91, 16.89 ± 5.77, and 29.96 ± 7.03 µg/mL, respectively. 

Our findings revealed that, based on the different antioxidant assays, DPPH radical scavenging activity, ABTS radical cation scavenging activity, and the ORAC assay, the mushroom aqueous extracts had strong antioxidant activities, comparable to those of the standard antioxidant, Trolox. Bakir et al. [87] compared the DPPH scavenging ability of *Pleurotus ostreatus* stored at different temperatures and found that the highest antioxidant activity IC_50_ = 0.321 mg/mL) was when the mushroom was stored at 20 °C. In the present study, we report lower IC_50_. Highly effective antioxidant capacities were recorded for *Pleurotus columbinus* (IC_50_ = 35.13 ± 3.2 µg/mL) and *Pleurotus sajor-caju* (IC_50_ = 40.91 ± 2.27 µg/mL). That of *Agaricus bisporus* was significantly lower (83.93 ± 0.62 µg/mL) (*p* ≤ 0.05).

In the ORAC assay, oxidative degradation or quenching of fluorescence probe by the proxy radicals is assessed. Antioxidants can prevent surrogate radicals from quenching the fluorescence probe. As a result, the time required to quench the fluorescent probe may vary depending on the sample’s antioxidant potential [88,89]. The IC_50_ values by ORAC assay for *Pleurotus sajor-caju* and *Pleurotus columbinus* (29.42 ± 3.21 and 32.00 ± 2.17 µg/mL, respectively) were also significantly higher than those of *Agaricus bisporus* (75.64 ± 4.65 µg/mL).

The strong fungal extracts’ antioxidant activity is commonly linked to high content of total phenols. However, our findings report that *Pleurotus columbinus* having the lowest phenolic content exerted the strongest antioxidant activity compared to the other tested mushrooms. This agrees with the previous study by Matuszewska et al. [5], in which it was reported that the fractions of the mushroom *Cerrena unicolor* with lowest phenol content had the strongest antioxidant effect and suggested that phenols may not be the key player in their antioxidant activity. 

The antioxidant effect of the test extracts may be attributed to more than one bioactive compound. All three mushroom extracts were rich in vitamin C (1.40, 0.75 and 0.96 mg/100 g for *Pleurotus sajor-caju*, *Pleurotus columbinus*, and *Agaricus bisporus,* respectively). Antioxidants, including vitamins C and E, are nonenzymatic scavengers. Since L-ascorbic acid (vitamin C) is water-soluble, it can fight free radical damage both within and outside the cell [90]. Vitamin C is most commonly linked to antioxidant effect; however, vitamin D’s antioxidant impact is one of the most recent noncalcemic activities proposed for this molecule [91]. It may initiate an antioxidant by inhibiting nitric oxide synthase (iNOS) or boosting glutamate concentrations [92]. The mushrooms also contain catechin, which has been linked to antioxidant effect. They may exert their effect through breaking chains and inhibiting lipid peroxidation of low-density lipoprotein (LDL) [93]. 

## 5. Conclusions

The three mushrooms *Pleurotus columbinus*, *Pleurotus sajor-caju*, and *Agaricus bisporus* contain a myriad of bioactive compounds. Aqueous extracts of the mushrooms have promising antiviral activities against Ad7 and HSV2 viruses. Cytotoxic effects were detected against cancer cell lines but not against normal human PBMCs. The extracts show potent antioxidant effects. *Pleurotus columbinus*, *Pleurotus sajor-caju* and *Agaricus bisporus* mushrooms offer significant medicinal potential for the prohibition and treatment of a variety of ailments.

## Figures and Tables

**Table 1:** Biochemical characterization of the three mushroom extracts.

Mushroom	Glucose Content (mg/g Extract)	Total Phenolic Content (mg/g Extract)	Total Flavonoid Content (mg/g Extract)	Total ProteinContent (mg/g Extract)
*Pleurotus columbinus*	82.24 ± 3.98 ^a^	18.16 ± 0.54 ^b^	ND *	0.08 ± 0.006 ^b^
*Pleurotus sajor-caju*	ND *	22.50 ± 1.53 ^b^	2.27 ± 0.18 ^b^	0.06 ± 0.005 ^b^
*Agaricus bisporus*	34.18 ± 1.48 ^b^	27.45 ± 0.8 ^a^	11.96 ± 1.81 ^a^	0.12 ± 0.004 ^a^

All results are expressed as mean ± SD from three experiments (*n* = 6). Values with different letters within the columns are significantly different (*p* ≤ 0.05), * ND: not detected in the mushroom.

**Table 2:** Phenolic and flavonoid composition of *mushroom Sp*. (µg/mL).

Compounds	*Pleurotus columbinus*	*Pleurotus sajor-caju*	*Agaricus bisporus*
Gallic acid	0.18 ± 0.05 ^a^	0.15 ± 0.03	ND *
Catechin	0.10 ± 0.03 ^a^	0.14 ± 0.02 ^a^	0.07 ± 0.01 ^b^
Chlorogenic acid	ND *	0.17 ± 0.04 ^a^	0.09 ± 0.02 ^b^
Caffeic acid	ND *	ND *	ND *
Rutin	ND *	ND *	ND *
Ellagic acid	ND *	ND *	ND *
Hesperidin	ND *	ND *	ND *
Querceitin	ND *	ND *	ND *
Kampeferol	ND *	ND *	ND *
Apigenin	ND *	ND *	ND *

All results are expressed as mean ± SD from three experiments (*n* = 3). Values with different letters within the rows are significantly different (*p* ≤ 0.05). ND * = Not detected.

**Table 3:** Total vitamin content of the test mushroom species.

	Vitamins	*Pleurotus columbinus*	*Pleurotus sajor-caju*	*Agaricus bisporus*
	Ascorbic acid (Vitamin C) (mg/100 g)	1.40 ± 0.39 ^a^	0.75 ± 0.23 ^a^	0.96 ± 0.08 ^a^
	Nicotinic acid (µg/100 g)	0.21 ± 0.02 ^b^	0.22 ± 0.03 ^b^	0.18 ± 0.02 ^c^
	Nicotinamide (µg/100 g)	0.05 ± 0.03 ^c^	0.03 ± 0.01 ^c^	0.07 ± 0.02 ^d^
Water soluble Vitamins	Pyridoxine (Vitamin B6) (µg/100 g)	0.25 ± 0.15 ^b^	0.27 ± 0.20 ^b^	0.30 ± 0.23 ^b^
	Folic acid (Vitamin B9) (µg/100 g)	ND *	ND *	ND *
	Thiamine (Vitamin B1) (µg/100 g)	ND *	ND *	ND *
	Riboflavin (Vitamin B2) (µg/100 g)	ND *	ND *	0.11 ± 0.02 ^c^
Fat soluble vitamins	Retinol (Vitamin A) (µg/100 g)	ND *	ND *	ND *
	Cholecalciferol (Vitamin D) (µg/100 g)	0.04 ± 0.02 ^c^	0.03 ± 0.01 ^c^	0.29 ± 0.03 ^b^
	Tocopherol (Vitamin E) (mg/100 g)	ND *	ND *	ND *

Each value is presented as the mean ± standard deviation (*n* = 3). Data with different superscript letters in the same column of variety indicate a significant difference (*p* ≤ 0.05). ND *: Not detected.

**Table 4:** Amino acids composition in three mushroom species.

	Amino Acids Content (g/100 g Protein)		
Amino Acids	*Pleurotus columbinus*	*Pleurotus sajor-caju*	*Agaricus bisporus*
Aspartic acid	ND *	ND *	ND *
Threonine	0.266	0.315	0.321
Serine	0.367	0.446	0.412
Glutamic acid	1.118	1.209	1.277
Proline	0.209	0.599	0.839
Glycine	0.365	0.388	0.302
Alanine	0.49	0.496	0.523
Cystine	0.206	0.201	0.223
Valine	0.241	0.257	0.268
Methionine	0.281	0.276	0.353
Isoleucine	0.117	0.124	0.127
Leucine	0.381	0.417	0.337
Tyrosine	0.167	0.176	0.197
Phenylalanine	0.202	0.217	0.161
Histadine	ND *	ND *	ND *
Lysine	0.343	0.347	0.299
Arginine	0.286	0.326	0.215
Total	5.04	5.75	5.58

ND *: Not detected

**Table 5:** Antiviral activities of the mushroom extracts.

Virus	Mushroom	CC_50_ (µg/mL)	IC_50_ (µg/mL)	SI
	*Pleurotus columbinus*	185.5	40.29	4.60
Ad7	*Pleurotus sajor-caju*	60.96	43.61	1.39
	*Agaricus bisporus*	8.339	15.28	0.54
	*Pleurotus columbinus*	17.615	20.04	0.87
HSV2	*Pleurotus sajor-caju*	25.43	34.34	0.74
	*Agaricus bisporus*	59.07	15.9	3.7

CC50, half-maximal cytotoxic concentration; IC50, half-maximal inhibitory concentration; SI, selectivity index = CC50/IC50.

**Table 6:** IC_50_ values of *Pleurotus columbinus*, *Pleurotus sajor-caju*, and *Agaricus bisporus* in the DPPH, ABTS radical scavenging, and ORAC assays.

		IC_50(_µg/mL)	
Mushroom	DPPH Radical Scavenging	ABTS Radical Cation Scavenging	ORAC Assay
*Pleurotus columbinus*	35.13 ± 3.27 ^c^	13.97 ± 4.91 ^c^	29.42 ± 3.21 ^b^
*Pleurotus sajor-caju*	40.91 ± 2.27 ^b^	16.89 ± 5.77 ^b^	32.00± 2.17 ^b^
*Agaricus bisporus*	83.93 ± 0.62 ^a^	29.96 ± 7.03 ^a^	75.64 ± 4.65 ^a^
*Trolox*	24.00 ± 0.87	40.00 ± 0.03	55.51 ± 0.06

All results are expressed as mean ± SD from three experiments (*n* = 6). Values with different letters within the columns are significantly different (*p* ≤ 0.05).

## Supplementary Materials

The following are available online at https://www.mdpi.com/article/10.3390/jof7080645/s1. Figure S1: HPLC chromatogram (λ280 nm) elution profile of pure phenolics and flavonoids standards (a) mixture (gallic acid, catechin, chlorogenic acid, caffeic acid, hespiridin, rutin, ellagic acid, quercetin, kampeferol, and apigenin) and the three mushroom extracts *Pleurotus columbinus*, *Pleurotus sajor-caju*, and *Agaricus bisporus* (b–d), Figure S2. HPLC chromatogram the standard mixture of water-soluble vitamins (a) and the three mushroom extracts *Pleurotus columbinus*, *Pleurotus sajor-caju*, and *Agaricus bisporus* (b–d), Figure S3: HPLC chromatogram of the standard mixture of fat-soluble vitamins (a), and the three mushroom extracts *Pleurotus columbinus*, *Pleurotus sajor-caju*, and *Agaricus bisporus* (b–d), Figure S4: Chromatogram of amino acids analysis of the mushroom isolates, Figure S5. Cytotoxicity concentration 50 (CC50) and inhibitory concentration 50 (IC50) of Hep 2 cells and Adv7, Figure S6. Cytotoxicity concentration (CC50) and 50% inhibitory concentration (IC50) on Vero cells and HSV 2, Figure S7. Effect of Trolox on the decay of fluorescein in ORAC assay. (A) Blank corrected linear regression curve of Trolox. (B) Signal curves of different Trolox concentrations and blank indicating the decay of fluorescein with different concentrations of Trolox, Figure S8. Signal curves of the three mushroom extracts and blank indicating the decay of fluorescein upon applying the samples. (1) *Pleurotus columbinus*, (2) *Pleurotus sajor-caju*, and (3) *Agaricus bisporus*. Table S1: Protein identification of *Pleurotus columbinus by Uniprot database*; Table S2: Protein identification of *Pleurotus sajor caju by Uniprot database*; Table S3: Protein identification of *Agricus bisporus by Uniprot database*.
