# Supplementary material for: Antiviral, Cytotoxic, and Antioxidant Activities of Three Edible Agaricomycetes Mushrooms: Pleurotus columbinus, Pleurotus sajor-caju, and Agaricus bisporus
Source: J Fungi (Basel). 2021 Aug 8;7(8):645. doi: 10.3390/jof7080645 (PMC8399653; doi:10.3390/jof7080645)
Supplement: Supplementary file 1 [file jof-07-00645-s001.zip › jof-1279201-supplementary.pdf]

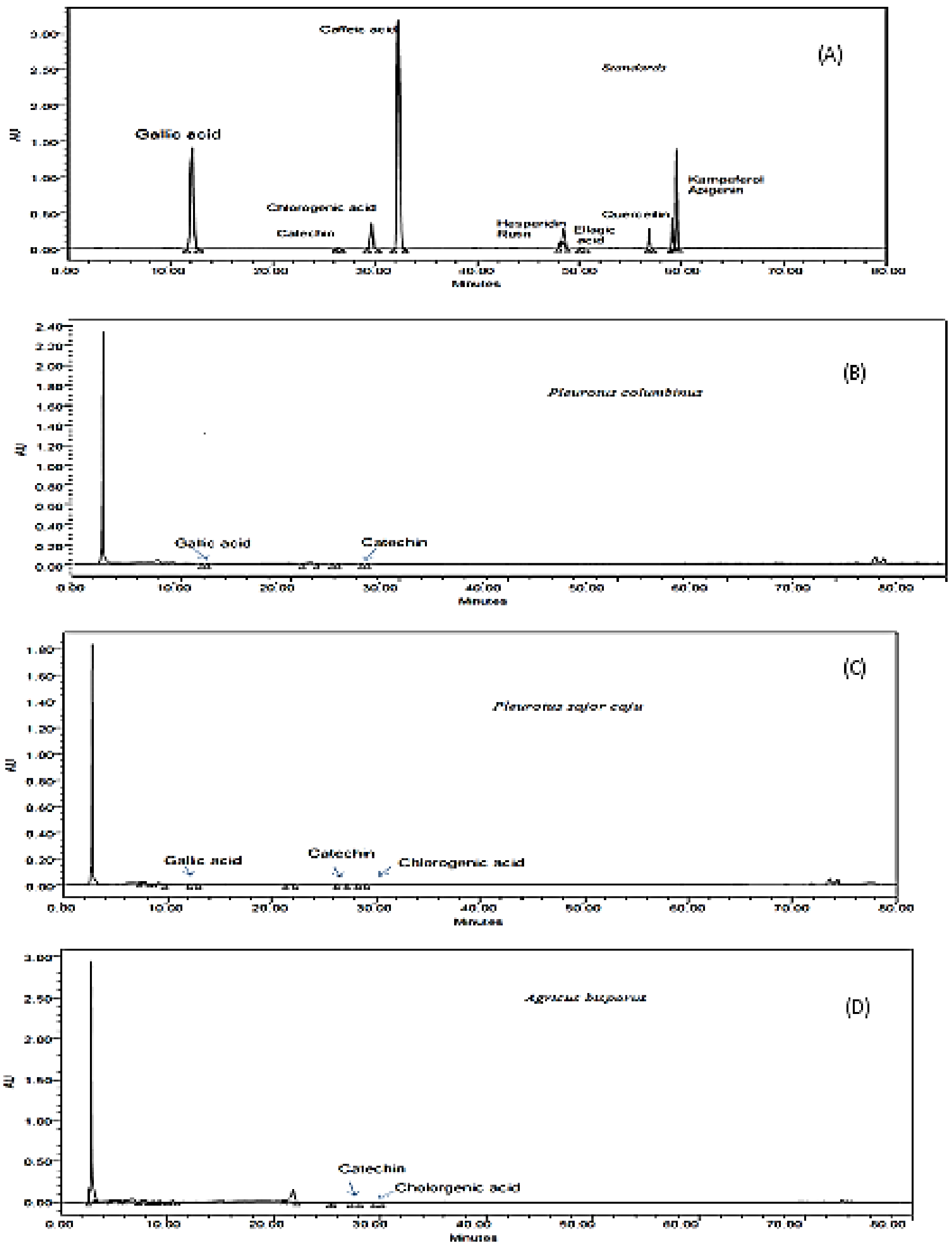

Figure S1: HPLC chromatogram (λ280 nm) elution profile of pure phenolics and flavonoids standards (a) mixture (Gallic acid, Catechin, Chlorogenic acid, Caffeic acid, Hespiridin, Rutin, Ellagic acid, Quercetin, Kampeferol and Apigenin) and the three mushroom extracts *Pleurotus columbinus*, *Pleurotus sajor caju*, and *Agricus bisporus* (b, c and d).

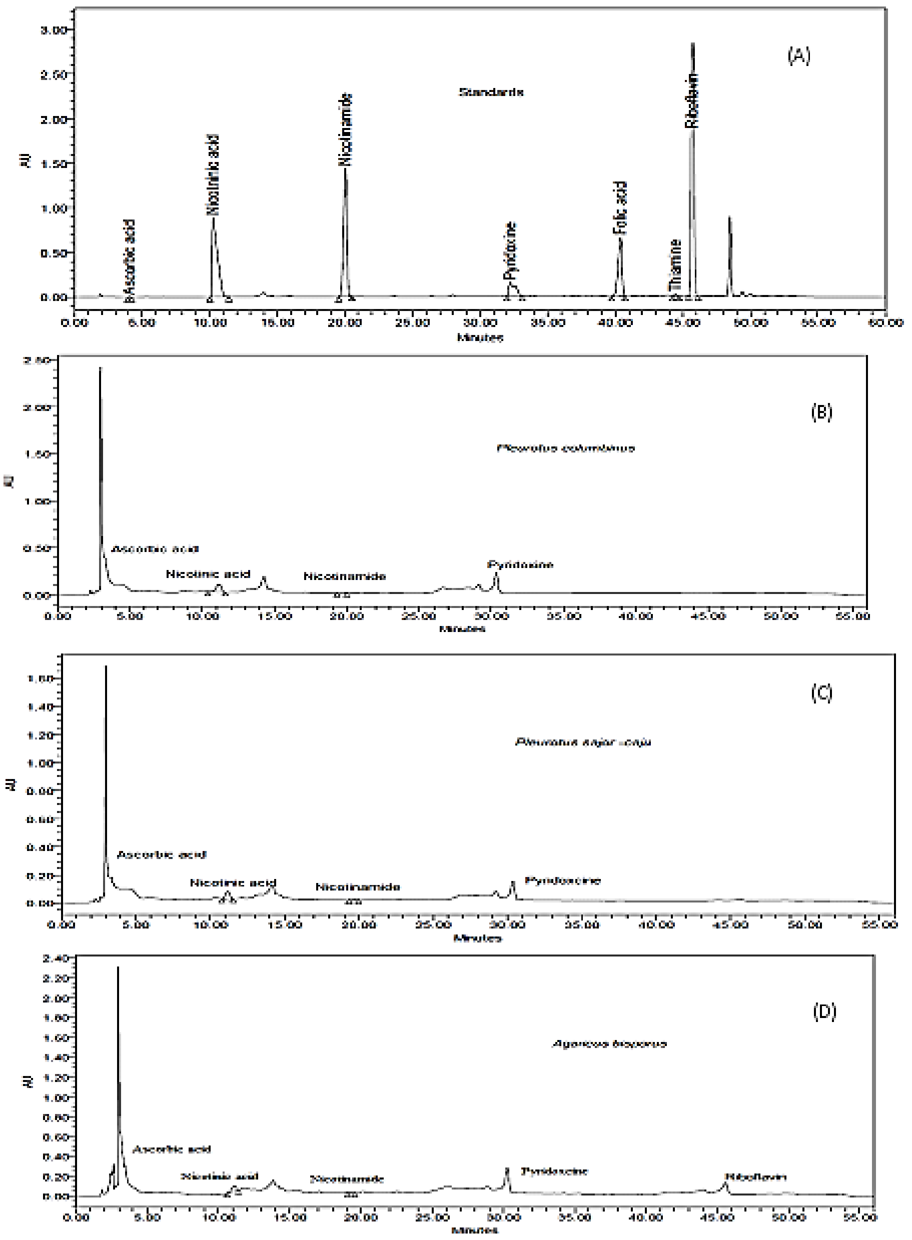

Figure S2. HPLC chromatogram the standard mixture of water-soluble vitamins (a) and the three mushroom extracts *Pleurotus columbinus*, *Pleurotus sajor caju,* and *Agricus bisporus* (b, c and d).

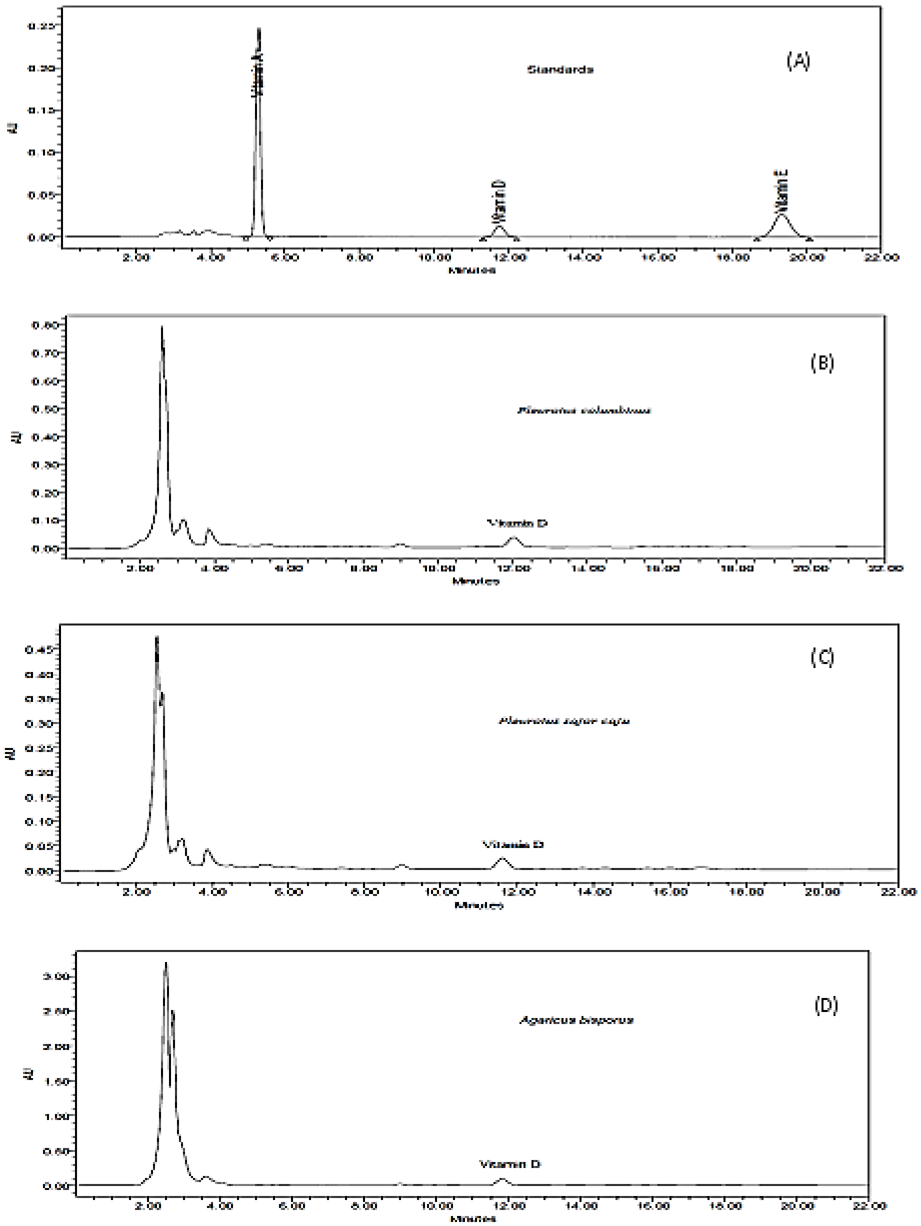

Figure S3: HPLC chromatogram of the standard mixture of fat-soluble vitamins (a), and the three mushroom extracts *Pleurotus columbinus*, *Pleurotus sajor caju*, and *Agricus bisporus* (b, c and d).

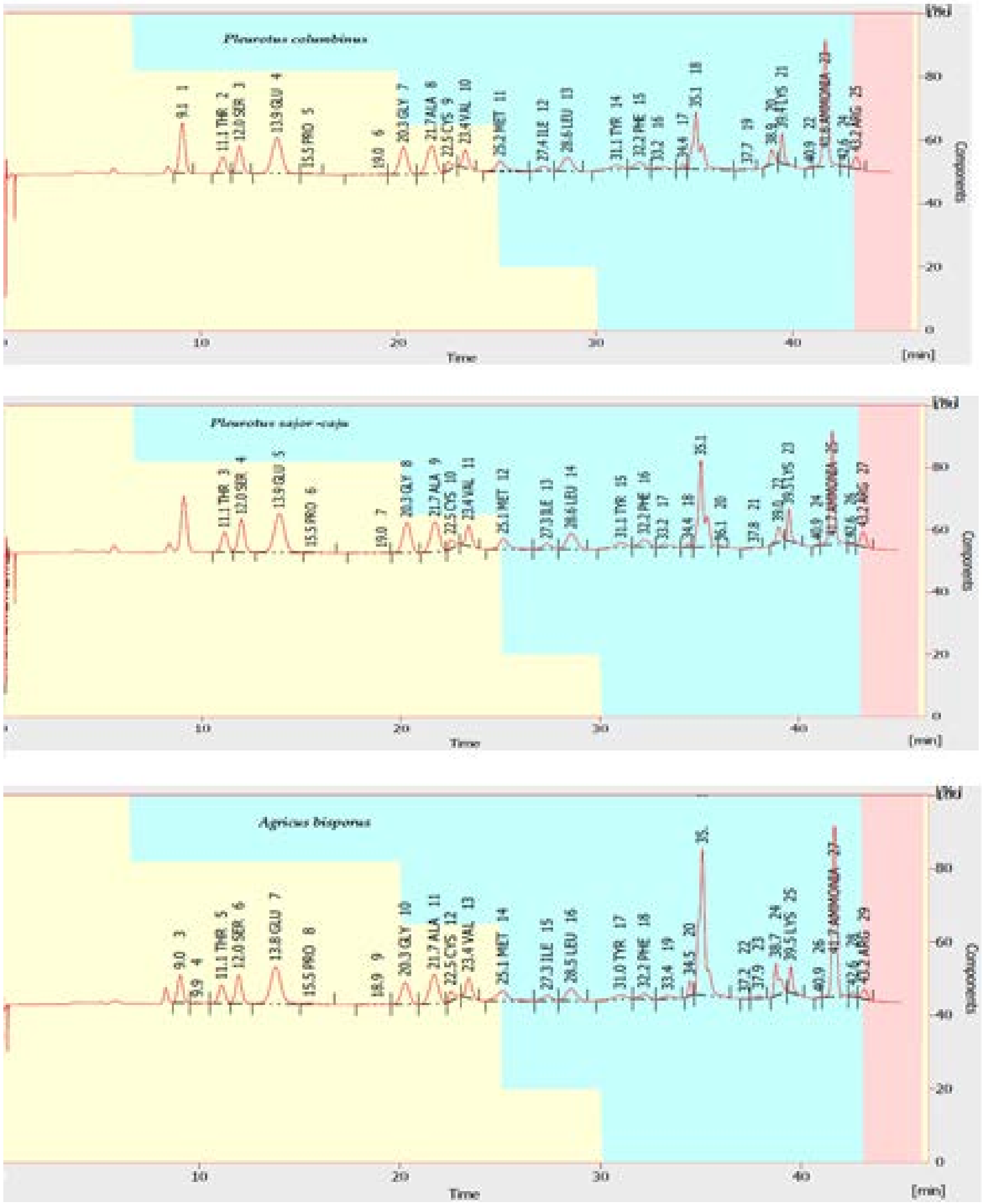

Figure S4: Chromatogram of amino acid analysis of the mushroom isolates.

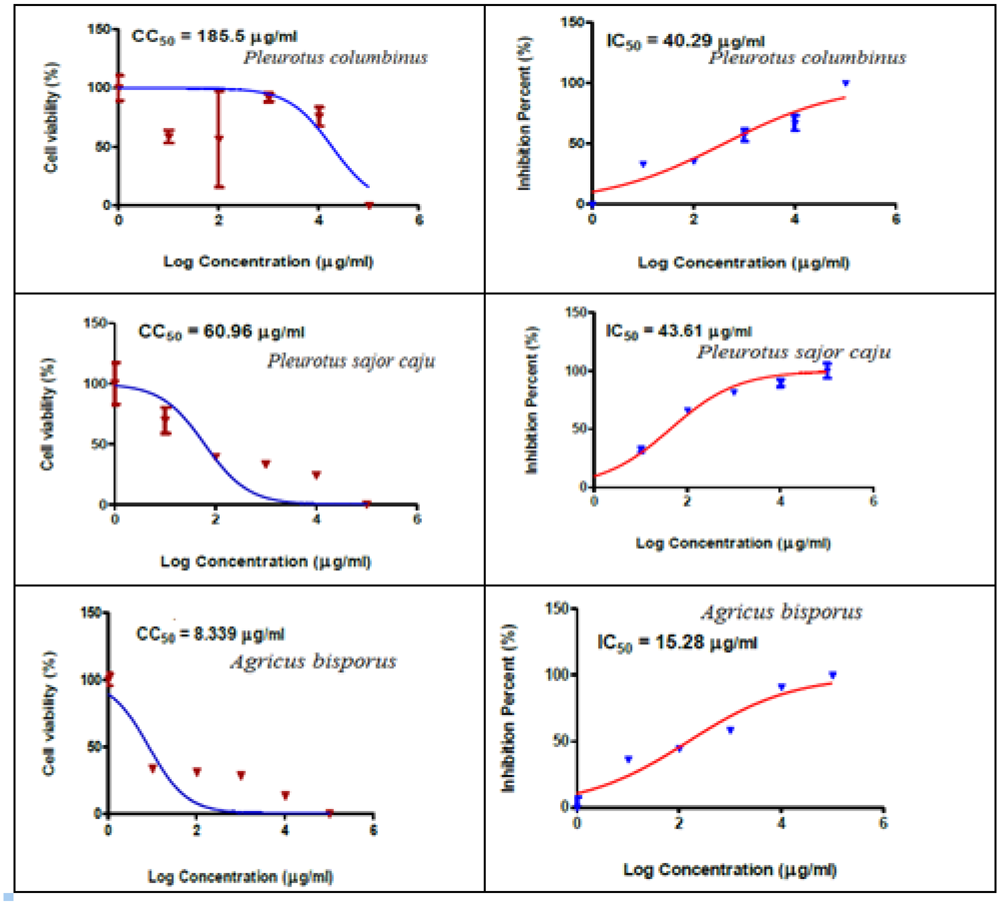

Figure S5. Cytotoxicity concentration 50 (CC50) and inhibitory concentration 50 (IC50) of Hep 2 cells and Adv7.

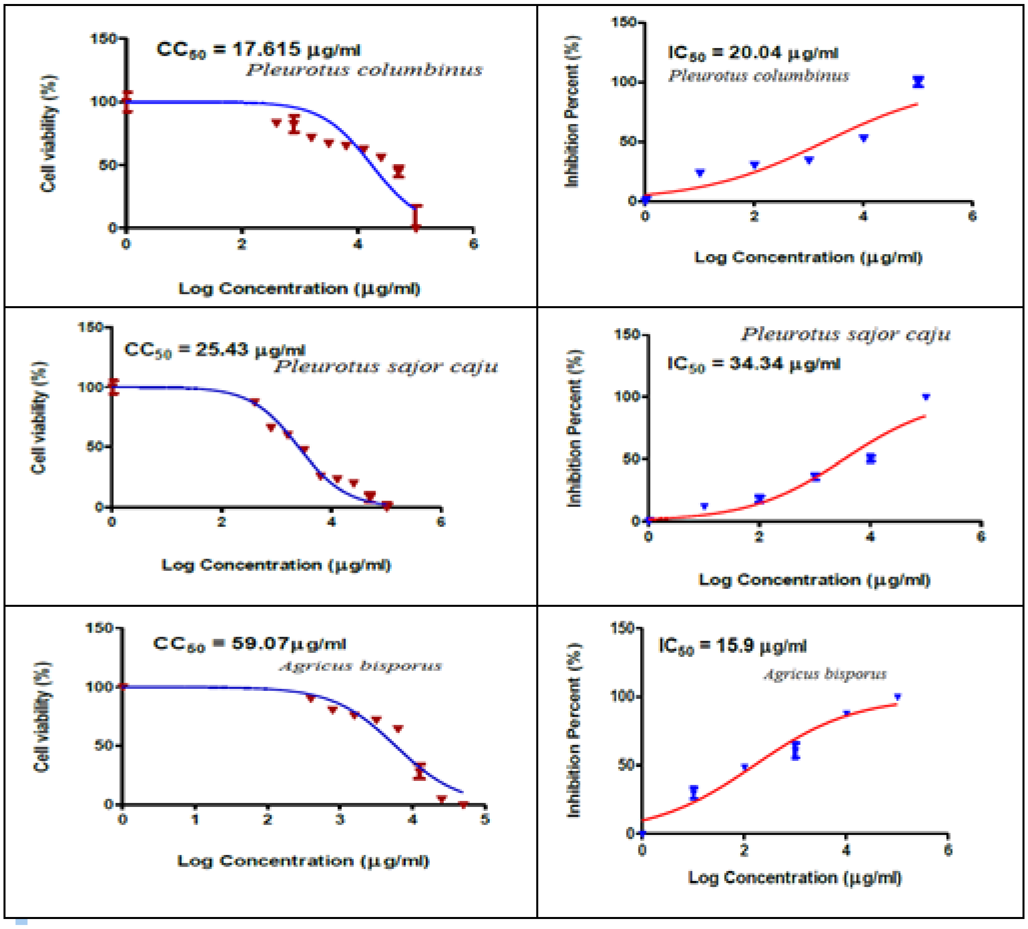

Figure S6. Cytotoxicity concentration (CC50) and 50% inhibitory concentration (IC50) on Vero cells and HSV 2.

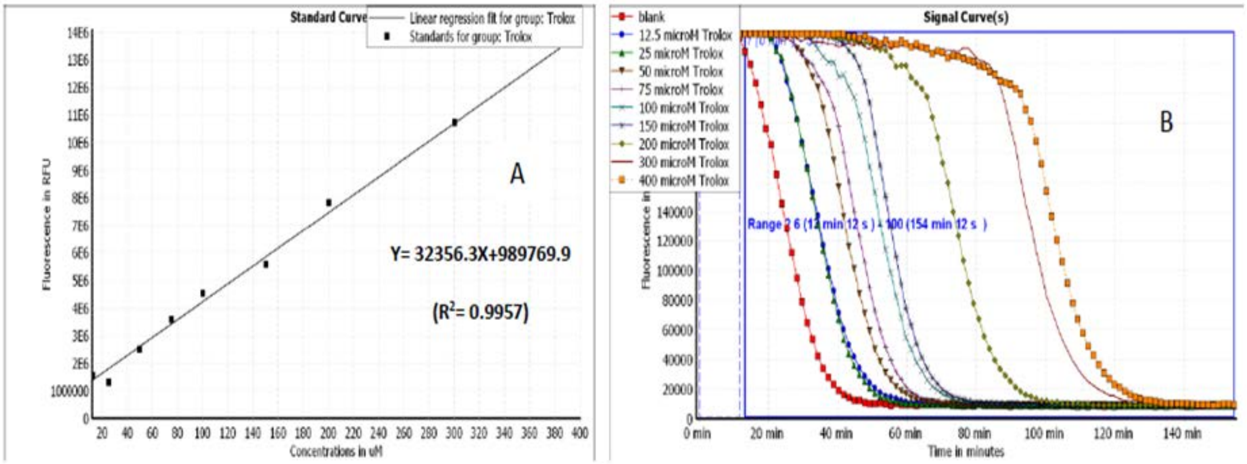

Figure S7. Effect of Trolox on the decay of fluorescein in ORAC assay. (A) blank corrected linear regression curve of Trolox. (B) signal curves of different Trolox concentrations and blank indicating the decay of fluorescein with different concentrations of Trolox.

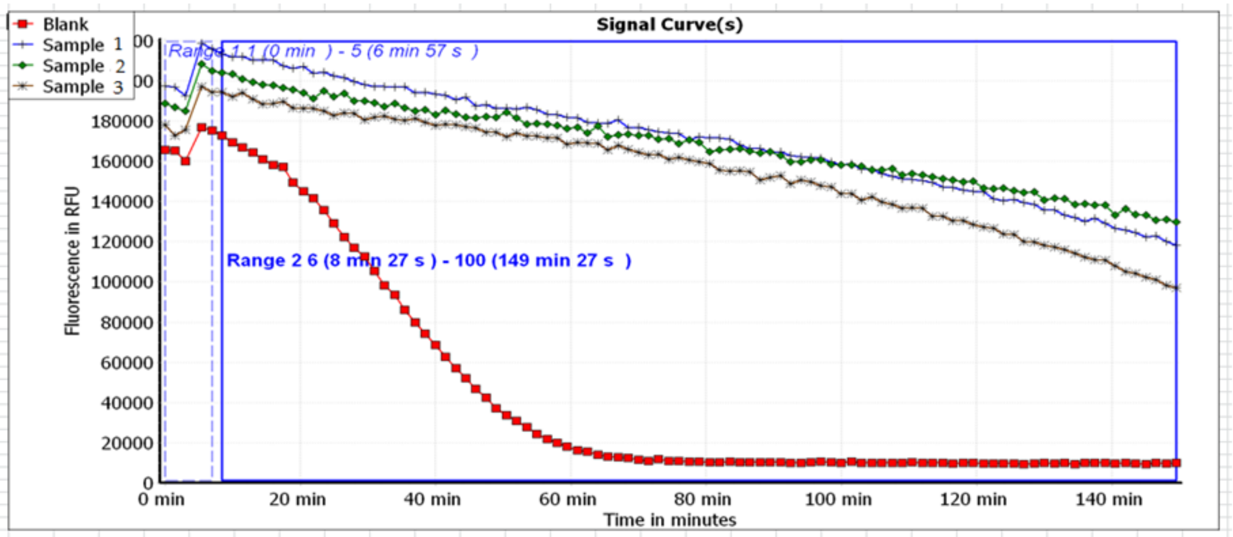

Figure S8. Signal curves of the three mushroom extracts and blank indicating the decay of fluorescein upon applying the samples. (1) *Pleurotus columbinus*, (2) *Pleurotus sajor caju* and (3) *Agricus bisporus.*

Table S1: Protein identification of *Pleurotus columbinus by Uniprot database*

Number	Accession number	Protein name	Peptide(95%)
1	tr|A0A067NW08|A0A067NW08_PLEOS	Elongation factor 2	50
2	tr|A0A067P0J4|A0A067P0J4_PLEOS	5-methyltetrahydropteroyltriglutamate	44
3	tr|A0A067NLR3|A0A067NLR3_PLEOS	ATP-citrate synthase	28
4	tr|A0A067NUP1|A0A067NUP1_PLEOS	Elongation factor 1-alpha	57
5	tr|A0A067P9U4|A0A067P9U4_PLEOS	HATPase_c domain-containing protein	28
6	tr|A0A482GPW1|A0A482GPW1_PLEOS	Catalase	36
7	tr|A0A2H4UZK7|A0A2H4UZK7_PLEOS	Catalase (Fragment)	36
8	tr|A0A067NHY5|A0A067NHY5_PLEOS	Catalase	36
9	tr|A0A067NS53|A0A067NS53_PLEOS	Pyruvate carboxylase	24
10	tr|A0A067N9N5|A0A067N9N5_PLEOS	Alpha-1,4 glucan phosphorylase	27
11	tr|A0A067NV82|A0A067NV82_PLEOS	Pyruvate kinase	27
12	tr|A0A067P9S7|A0A067P9S7_PLEOS	Glycosyltransferase	34
13	tr|A0A067P4U9|A0A067P4U9_PLEOS	Phosphopyruvate hydratase	41
14	tr|A0A067NRP1|A0A067NRP1_PLEOS	14_3_3 domain-containing protein	31
15	tr|Q9UWF3|Q9UWF3_PLESA	Tubulin beta chain	29
16	tr|A0A067N725|A0A067N725_PLEOS	Tubulin beta chain	29
17	tr|A0A067NP23|A0A067NP23_PLEOS	Pleurotolysin B	32
18	tr|A0A067NEB2|A0A067NEB2_PLEOS	Aldo_ket_red domain-containing protein	35
19	tr|A0A067P303|A0A067P303_PLEOS	6-phosphogluconate dehydrogenase, decarboxylating	32
20	tr|A0A067N302|A0A067N302_PLEOS	Adenosylhomocysteinase	20
21	tr|A0A067NGR8|A0A067NGR8_PLEOS	Ribos_L4_asso_C domain-containing protein	19
22	tr|A0A067NII7|A0A067NII7_PLEOS	Formate dehydrogenase	24
23	tr|A0A067NJB6|A0A067NJB6_PLEOS	Carbohydrate-binding module family 12 protein	21
24	tr|A0A067NI01|A0A067NI01_PLEOS	UTP--glucose-1-phosphate uridylyltransferase	23
25	tr|A0A067NVZ7|A0A067NVZ7_PLEOS	D-fructose-6-phosphate amidotransferase	18
26	tr|A0A067NYL0|A0A067NYL0_PLEOS	UDP-glucose 6-dehydrogenase	17
27	tr|A0A067NTI0|A0A067NTI0_PLEOS	ATP synthase subunit alpha	17
28	tr|A0A067NCW7|A0A067NCW7_PLEOS	Ostreolysin-like protein	41
29	sp|P83467|OLYA6_PLEOS	Ostreolysin A6	39
30	tr|A0A067NZL3|A0A067NZL3_PLEOS	Transaldolase	23
31	tr|W8SJ43|W8SJ43_PLEER	Glutamine synthetase	23
32	tr|A0A2R8G1D5|A0A2R8G1D5_PLEOS	Glutamine synthetase	23
33	tr|A0A067NQW2|A0A067NQW2_PLEOS	Glutamine synthetase	23
34	tr|A0A067N544|A0A067N544_PLEOS	PKS_ER domain-containing protein	19
35	tr|A0A067NIX1|A0A067NIX1_PLEOS	Malate dehydrogenase	23
36	tr|A0A387LBC4|A0A387LBC4_PLEOS	Manganese lipoxygenase	18
37	tr|A0A067N2A8|A0A067N2A8_PLEOS	Manganese lipoxygenase	18

38	tr|A0A067N4M3|A0A067N4M3_PLEOS	Thioredoxin domain	15
39	tr|A0A067NSQ2|A0A067NSQ2_PLEOS	Inorganic diphosphatase	13
40	tr|A0A067N792|A0A067N792_PLEOS	Endoplasmic reticulum chaperone BiP	14
41	tr|A0A067NER0|A0A067NER0_PLEOS	Tubulin alpha chain	16
42	tr|A0A067NXS6|A0A067NXS6_PLEOS	Glucose-6-phosphate 1-dehydrogenase	13
43	tr|A0A067NL73|A0A067NL73_PLEOS	Malate dehydrogenase	15
44	tr|A0A067NHP6|A0A067NHP6_PLEOS	40S ribosomal protein S0	13
45	tr|D2JY80|D2JY80_PLEOS	RRM domain-containing protein	16
46	tr|A0A067NHG1|A0A067NHG1_PLEOS	Aldedh domain-containing protein	14
47	tr|A0A067N5V4|A0A067N5V4_PLEOS	Hemerythrin domain-containing protein	13
48	tr|A0A067PB97|A0A067PB97_PLEOS	Phosphoglycerate kinase	11
49	tr|A0A067NR11|A0A067NR11_PLEOS	Protein disulfide-isomerase	10
50	tr|A0A067NEK4|A0A067NEK4_PLEOS	Peptidyl-prolyl cis-trans isomerase	32
51	tr|A0A067NQG0|A0A067NQG0_PLEOS	40S ribosomal protein S1	11
52	tr|A0A067NS51|A0A067NS51_PLEOS	KH type-2 domain-containing protein	11
53	tr|G9MD63|G9MD63_PLEOS	Glyceraldehyde-3-phosphate dehydrogenase	22
54	tr|A0A067NKG0|A0A067NKG0_PLEOS	Glyceraldehyde-3-phosphate dehydrogenase	22
55	tr|H1AFL8|H1AFL8_PLEOS	Glyceraldehyde-3-phosphate dehydrogenase	18
56	tr|H1AFL5|H1AFL5_PLEOS	Glyceraldehyde-3-phosphate dehydrogenase	18
57	tr|A0A067NT84|A0A067NT84_PLEOS	Sugar phosphate phosphatase	10
58	tr|A0A067NYA3|A0A067NYA3_PLEOS	60S ribosomal protein L20	9
59	sp|O94739|CALM_PLEOS	Calmodulin	9
60	sp|P11120|CALM_PLECO	Calmodulin	8
61	tr|A0A067NZG5|A0A067NZG5_PLEOS	40S ribosomal protein S7	13
62	tr|A0A067NP59|A0A067NP59_PLEOS	Multifunctional fusion protein	9
63	tr|A0A067NV87|A0A067NV87_PLEOS	Glucose-6-phosphate isomerase	8
64	tr|A0A067P1D3|A0A067P1D3_PLEOS	Septin-type G domain-containing protein	9
65	tr|A0A067P0D8|A0A067P0D8_PLEOS	H(+)-transporting two-sector ATPase	8
66	tr|A0A067NQW4|A0A067NQW4_PLEOS	Superoxide dismutase	15
67	tr|A0A067NZS0|A0A067NZS0_PLEOS	ATP synthase subunit beta	16
68	tr|A0A067NHZ5|A0A067NHZ5_PLEOS	40S ribosomal protein S8	9
69	tr|A0A067NFT1|A0A067NFT1_PLEOS	40S ribosomal protein S4	8
70	tr|A0A067P3U3|A0A067P3U3_PLEOS	Aldo_ket_red domain-containing protein	10
71	tr|A0A067P657|A0A067P657_PLEOS	Aldo_ket_red domain-containing protein	7
72	tr|A0A067P101|A0A067P101_PLEOS	Ribosomal_L2_C domain-containing protein	7
73	tr|A0A067NI26|A0A067NI26_PLEOS	HTH cro/C1-type domain-containing protein	13
74	tr|A0A067NBU9|A0A067NBU9_PLEOS	NAD-specific glutamate dehydrogenase	6

75	tr|A0A067NJC3|A0A067NJC3_PLEOS	WD_REPEATS_REGION domain-containing protein	8
76	tr|A0A067PAP5|A0A067PAP5_PLEOS	GTP-binding nuclear protein	10
77	tr|A0A067PBA8|A0A067PBA8_PLEOS	Nucleoside diphosphate kinase	13
78	tr|A0A067NI86|A0A067NI86_PLEOS	Carbohydrate-binding module family 13 protein	22
79	tr|A0A067NZB1|A0A067NZB1_PLEOS	S-adenosylmethionine synthase	8
80	tr|A0A067NUF2|A0A067NUF2_PLEOS	Ribosomal_S13_N domain-containing protein	6
81	tr|A0A067N3J5|A0A067N3J5_PLEOS	Serine hydroxymethyltransferase	6
82	tr|A0A067NEY2|A0A067NEY2_PLEOS	60S ribosomal protein L8 (Fragment)	7
83	tr|A0A067NBS5|A0A067NBS5_PLEOS	ACB domain-containing protein	7
84	tr|A0A067N929|A0A067N929_PLEOS	Ketol-acid reductoisomerase, mitochondrial	7
85	tr|A0A067NF95|A0A067NF95_PLEOS	Septin-type G domain-containing protein	8
86	tr|A0A067NKH2|A0A067NKH2_PLEOS	Aldo_ket_red domain-containing protein	7
87	tr|A0A067NSA9|A0A067NSA9_PLEOS	Rab GDP dissociation inhibitor	9
88	tr|A0A067P2E6|A0A067P2E6_PLEOS	Septin-type G domain-containing protein	7
89	tr|A0A067NAM1|A0A067NAM1_PLEOS	Aldo_ket_red domain-containing protein	6
90	tr|A0A067NZ52|A0A067NZ52_PLEOS	Ribosomal_L18e/L15P domain-containing protein	8
91	tr|A0A067PCH8|A0A067PCH8_PLEOS	Aldo_ket_red domain-containing protein	10
92	tr|Q96TW1|Q96TW1_PLEOS	Putative ubiquitin (Fragment)	11
93	tr|A0A067NZF5|A0A067NZF5_PLEOS	Ubiquitin-like domain-containing protein	11
94	tr|A0A067NR75|A0A067NR75_PLEOS	Ubiquitin-like domain-containing protein	11
95	tr|A0A067NT90|A0A067NT90_PLEOS	Citrulline--aspartate ligase	6
96	tr|A0A067NWF1|A0A067NWF1_PLEOS	Phosphoenolpyruvate carboxykinase (ATP)	5
97	tr|A0A067NUA5|A0A067NUA5_PLEOS	S4 RNA-binding domain-containing protein	7
98	tr|A0A067P0R2|A0A067P0R2_PLEOS	Tubulin alpha chain	16
99	tr|A0A067NED1|A0A067NED1_PLEOS	Coronin	5
100	tr|A0A067NTA7|A0A067NTA7_PLEOS	Histone H4	6
101	tr|A0A067P3W4|A0A067P3W4_PLEOS	Histone H4	5
102	tr|A0A067NTP4|A0A067NTP4_PLEOS	40S ribosomal protein S26 (Fragment)	6
103	tr|A0A067P4S5|A0A067P4S5_PLEOS	60S ribosomal protein L13	11
104	tr|A0A067NCV7|A0A067NCV7_PLEOS	Vacuolar proton pump subunit B	6
105	tr|A0A067NS47|A0A067NS47_PLEOS	Septin-type G domain-containing protein	7
106	tr|A0A067N8D2|A0A067N8D2_PLEOS	RRM domain-containing protein	19
107	tr|A0A067P007|A0A067P007_PLEOS	PABS domain-containing protein	5
108	tr|D2JY73|D2JY73_PLEOS	Ribosomal_L16 domain-containing protein	9
109	tr|A0A067NTH6|A0A067NTH6_PLEOS	Ribosomal_L16 domain-containing protein	9
110	tr|A0A067N9I3|A0A067N9I3_PLEOS	Rieske domain-containing protein	8

111	tr|A0A067NY20|A0A067NY20_PLEOS	S-(hydroxymethyl)glutathione dehydrogenase	5
112	tr|A0A067NUU3|A0A067NUU3_PLEOS	40S ribosomal protein S6	6
113	tr|A0A067P305|A0A067P305_PLEOS	Transketolase	6
114	tr|A0A067P6E3|A0A067P6E3_PLEOS	Histone H2B	8
115	tr|A0A067NVC3|A0A067NVC3_PLEOS	Histone H2B	8
116	tr|A0A067NZ73|A0A067NZ73_PLEOS	Histone H2B	8
117	tr|A0A067NLW0|A0A067NLW0_PLEOS	UBA_e1_C domain-containing protein	7
118	tr|A0A067N8V5|A0A067N8V5_PLEOS	Peptidase_M24 domain-containing protein	6
119	tr|A0A067NLD9|A0A067NLD9_PLEOS	Transket_pyr domain-containing protein	6
120	tr|A0A067NB23|A0A067NB23_PLEOS	Extracellular metalloproteinase	8
121	tr|A0A067NLX2|A0A067NLX2_PLEOS	Cytochrome b5 heme-binding domain-containing protein	6
122	tr|A0A067NW47|A0A067NW47_PLEOS	S5 DRBM domain-containing protein	8
123	tr|A0A067P4P1|A0A067P4P1_PLEOS	Ribosomal_S17_N domain-containing protein	5
124	tr|A0A067P301|A0A067P301_PLEOS	Ribosomal protein L15	6
125	tr|D2JY79|D2JY79_PLEOS	Ribosomal protein L15	6
126	tr|A0A067P056|A0A067P056_PLEOS	Aldedh domain-containing protein	6
127	tr|A0A067NL37|A0A067NL37_PLEOS	Aconitate hydratase, mitochondrial	9
128	tr|A0A067NIF5|A0A067NIF5_PLEOS	Ribosomal_S7 domain-containing protein	8
129	tr|A0A067NG95|A0A067NG95_PLEOS	Dipeptidyl-peptidase V	6
130	tr|A0A067NV48|A0A067NV48_PLEOS	PKS_ER domain-containing protein	4
131	tr|A0A067NY35|A0A067NY35_PLEOS	M20_dimer domain-containing protein	4
132	tr|A0A067NM16|A0A067NM16_PLEOS	60S acidic ribosomal protein P0	5
133	tr|A0A067NMU9|A0A067NMU9_PLEOS	60S ribosomal protein L36	4
134	tr|A0A067NYA5|A0A067NYA5_PLEOS	Carbohydrate-binding module family 13 protein	8
135	tr|A0A067N2Q3|A0A067N2Q3_PLEOS	Flavodoxin-like domain-containing protein	7
136	tr|D2JY75|D2JY75_PLEOS	Flavodoxin-like domain-containing protein	6
137	tr|A0A067NTK7|A0A067NTK7_PLEOS	T-complex protein 1 subunit eta	4
138	tr|A0A067NZD8|A0A067NZD8_PLEOS	Small COPII coat GTPase SAR1	4
139	tr|A0A067NP91|A0A067NP91_PLEOS	Ribosomal protein	5
140	tr|A0A067NSY7|A0A067NSY7_PLEOS	Aspzincin_M35 domain-containing protein	8
141	sp|P81055|PLMP_PLEOS	Peptidyl-Lys metalloendopeptidase	8
142	tr|A0A067NX03|A0A067NX03_PLEOS	Ribosomal_L28e domain-containing protein	4
143	tr|A0A067NU43|A0A067NU43_PLEOS	Adenosine kinase OS=Pleurotus ostreatus	5
144	tr|A0A067NEN6|A0A067NEN6_PLEOS	Core subunit of the ubiquinol-cytochrome c reductase complex, QCR1	4
145	tr|A0A067NQG3|A0A067NQG3_PLEOS	Mitochondrial intermediate peptidase	6

146	tr|A0A067NUL0|A0A067NUL0_PLEOS	Pyrid_ox_like domain-containing protein	3
147	tr|A0A067NC11|A0A067NC11_PLEOS	Ribosomal_L23eN domain-containing protein (Fragment)	5
148	tr|A0A067NZJ1|A0A067NZJ1_PLEOS	Proteasome subunit alpha type	4
149	tr|A0A067NSH1|A0A067NSH1_PLEOS	DUF2235 domain-containing protein	5
150	tr|A0A067N3K5|A0A067N3K5_PLEOS	NmrA domain-containing protein	4
151	tr|A0A067NFQ9|A0A067NFQ9_PLEOS	NmrA domain-containing protein	4
152	tr|A0A067PC25|A0A067PC25_PLEOS	E3 ubiquitin ligase complex SCF subunit	5
153	tr|A0A067NPJ8|A0A067NPJ8_PLEOS	Alpha-amylase	5
154	tr|A0A067NYX2|A0A067NYX2_PLEOS	Pyruvate dehydrogenase E1 component subunit alpha	4
155	tr|A0A067NMW5|A0A067NMW5_PLEOS	Peptidase A1 domain-containing protein (Fragment)	4
156	tr|U6A6W7|U6A6W7_PLEOS	Aspartic protease	3
157	tr|A0A067NU68|A0A067NU68_PLEOS	Thioredoxin	5
158	tr|A0A067NHX4|A0A067NHX4_PLEOS	Malate synthase	4
159	tr|A0A067PAB4|A0A067PAB4_PLEOS	HMA domain-containing protein	4
160	tr|A0A067PAP3|A0A067PAP3_PLEOS	PKS_ER domain-containing protein	5
161	tr|A0A067NHH3|A0A067NHH3_PLEOS	Aldedh domain-containing protein	8
162	tr|A0A067NNJ9|A0A067NNJ9_PLEOS	1,4-alpha-glucan-branching enzyme (Fragment)	4
163	tr|A0A067NDZ6|A0A067NDZ6_PLEOS	Proteasome subunit beta	3
164	tr|A0A067NPY7|A0A067NPY7_PLEOS	RanBD1 domain-containing protein	6
165	tr|A0A067NU35|A0A067NU35_PLEOS	T-complex protein 1 subunit gamma	3
166	tr|A0A067NAX7|A0A067NAX7_PLEOS	Inosine-5'-monophosphate dehydrogenase	3
167	tr|A0A067NEH1|A0A067NEH1_PLEOS	ATP synthase subunit 4, mitochondrial	3
168	tr|A0A067P0M5|A0A067P0M5_PLEOS	Ribosomal_L18_c domain-containing protein (Fragment)	4
169	tr|A0A067NSE6|A0A067NSE6_PLEOS	Ribosomal protein L19	5
170	tr|A0A067P077|A0A067P077_PLEOS	KOW domain-containing protein (Fragment)	8
171	tr|A0A067NMW1|A0A067NMW1_PLEOS	S10_plectin domain-containing protein	8
172	tr|A0A067NYE7|A0A067NYE7_PLEOS	Peptide hydrolase	4
173	tr|A0A067NJ33|A0A067NJ33_PLEOS	Eukaryotic translation initiation factor 3 subunit E	3
174	tr|A0A067P1D2|A0A067P1D2_PLEOS	Cell division control protein 42 homolog	3
175	tr|A0A067PBC2|A0A067PBC2_PLEOS	AAA domain-containing protein	3
176	tr|A0A067NWN7|A0A067NWN7_PLEOS	Actin-related protein 2/3 complex subunit 4	3
177	tr|A0A067P0D4|A0A067P0D4_PLEOS	Proteasome subunit alpha type	5
178	tr|A0A067NS12|A0A067NS12_PLEOS	HABP4_PAI-RBP1 domain-containing protein	3
179	tr|A0A067NIB4|A0A067NIB4_PLEOS	DLH domain-containing protein	4

Table S2: Protein identification of *Pleurotus sajor caju by Uniprot database*

180	tr|A0A067P7Q0|A0A067P7Q0_PLEOS	Ribosomal_L18e/L15P domain-containing protein	7
181	tr|A0A067NPE8|A0A067NPE8_PLEOS	40S ribosomal protein S12	3
182	tr|A0A067NSN4|A0A067NSN4_PLEOS	Methylmalonate-semialdehyde dehydrogenase (CoA acylating)	6
183	tr|A0A067PA05|A0A067PA05_PLEOS	Succinate dehydrogenase [ubiquinone] flavoprotein subunit, mitochondrial	4
184	tr|A0A067NG45|A0A067NG45_PLEOS	Glycosyltransferase family 69 protein	6
185	tr|A0A067NXY7|A0A067NXY7_PLEOS	60S ribosomal protein L27	4
186	tr|A0A067NY79|A0A067NY79_PLEOS	PCI domain-containing protein	3
187	tr|D2JY85|D2JY85_PLEOS	Histone H2A	6
188	tr|A0A067P7W0|A0A067P7W0_PLEOS	Histone H2A	4
189	tr|A0A067NY26|A0A067NY26_PLEOS	Histone H2A (Fragment)	4
190	tr|A0A067NG34|A0A067NG34_PLEOS	Histone H2A (Fragment)	4
191	tr|A0A067NVU4|A0A067NVU4_PLEOS	Histone H2A (Fragment)	6
192	tr|A0A067NVP5|A0A067NVP5_PLEOS	GST C-terminal domain-containing protein	3
193	tr|A0A067NDL4|A0A067NDL4_PLEOS	Actin-depolymerizing factor 1	4
194	tr|A0A067NCB2|A0A067NCB2_PLEOS	Ribosomal_S10 domain-containing protein	4
195	tr|A0A067NWB7|A0A067NWB7_PLEOS	RRM domain-containing protein	17
196	tr|A0A067P3F5|A0A067P3F5_PLEOS	AB hydrolase-1 domain-containing protein	3
197	tr|A0A067NYZ2|A0A067NYZ2_PLEOS	Polyadenylate-binding protein	4
198	tr|A0A067NKI6|A0A067NKI6_PLEOS	Eukaryotic translation initiation factor 5A	5
199	tr|A0A067P4E6|A0A067P4E6_PLEOS	DLH domain-containing protein	5
200	tr|A0A067NYE3|A0A067NYE3_PLEOS	Superoxide dismutase	3
201	tr|A0A482GME7|A0A482GME7_PLEOS	Superoxide dismutase	2
202	tr|V5MZX5|V5MZX5_PLEOS	Superoxide dismutase	2
203	tr|V5MZR6|V5MZR6_PLEOS	Superoxide dismutase	2
204	tr|A0A067NUQ4|A0A067NUQ4_PLEOS	UV excision repair protein RAD23	3
205	tr|A0A067N7K3|A0A067N7K3_PLEOS	GMC_OxRdtase_N domain-containing protein	3
206	tr|A0A067P098|A0A067P098_PLEOS	Malic enzyme	2
207	tr|A0A067NKF3|A0A067NKF3_PLEOS	Citrate synthase	3
208	tr|A0A067NUN3|A0A067NUN3_PLEOS	Plasma membrane ATPase	3
209	tr|A0A067P6N3|A0A067P6N3_PLEOS	Glutaredoxin domain-containing protein	2
210	tr|A0A067NF75|A0A067NF75_PLEOS	Redoxin domain-containing protein	6
211	tr|A0A067NZF1|A0A067NZF1_PLEOS	SBDS domain-containing protein	4
212	tr|A0A067NKK5|A0A067NKK5_PLEOS	CCT-theta	2
213	tr|A0A067NG89|A0A067NG89_PLEOS	Extracellular metalloproteinase	2
214	tr|A0A067NTN6|A0A067NTN6_PLEOS	GLTP domain-containing protein	2
215	tr|A0A067N5Y3|A0A067N5Y3_PLEOS	Dihydrolipoyl dehydrogenase	3
216	tr|A0A067NBL2|A0A067NBL2_PLEOS	60S ribosomal protein L41	3

217	tr|O94154|O94154_PLEOS	Catalase	3
218	tr|A0A067NWW1|A0A067NWW1_PLEOS	Catalase	3
219	tr|A0A482GQN6|A0A482GQN6_PLEOS	Catalase	2
220	tr|A0A2H4UZK6|A0A2H4UZK6_PLEOS	Catalase (Fragment)	2
221	tr|A0A067NMW7|A0A067NMW7_PLEOS	ATP synthase subunit d, mitochondrial	3
222	tr|A0A067N481|A0A067N481_PLEOS	Glycoside hydrolase family 5 protein	2
223	tr|A0A067P946|A0A067P946_PLEOS	ATP synthase subunit gamma (Fragment)	2
224	tr|A0A067NC76|A0A067NC76_PLEOS	Obg-like ATPase 1	2
225	tr|A0A067NWV6|A0A067NWV6_PLEOS	Small nuclear ribonucleoprotein Sm D3	3
226	tr|A0A067NS58|A0A067NS58_PLEOS	Clathrin heavy chain	2
227	tr|A0A067NET3|A0A067NET3_PLEOS	HIT domain protein	2
228	tr|A0A067P1R2|A0A067P1R2_PLEOS	Alpha-NAC	2
229	tr|A0A067NUN8|A0A067NUN8_PLEOS	ATP synthase subunit 5, mitochondrial	3
230	tr|A0A067NSA2|A0A067NSA2_PLEOS	Ribosomal_L7Ae domain-containing protein	2
231	tr|A0A067NEB1|A0A067NEB1_PLEOS	Lipase_3 domain-containing protein	2
232	tr|A0A067N8U4|A0A067N8U4_PLEOS	6-phosphogluconate dehydrogenase, decarboxylating	30
233	tr|A0A067NDT5|A0A067NDT5_PLEOS	Proteasome endopeptidase complex	3
234	tr|A0A067NKW8|A0A067NKW8_PLEOS	Fructose-bisphosphate aldolase	7
235	tr|A0A067NFS2|A0A067NFS2_PLEOS	Ribosomal protein L37	2
236	tr|A0A067NVT9|A0A067NVT9_PLEOS	RRM domain-containing protein	3
237	tr|A0A067NVE7|A0A067NVE7_PLEOS	Methionine adenosyltransferase 2 subunit beta	3
238	tr|A0A067NBR1|A0A067NBR1_PLEOS	AB hydrolase-1 domain-containing protein	2
239	tr|A0A067NW13|A0A067NW13_PLEOS	NAD_binding_2 domain-containing protein	3
240	sp|Q7M4T6|PIA1_PLEOS	Serine proteinase inhibitor IA-1	1
241	sp|Q7M4T5|PIA2_PLEOS	Serine proteinase inhibitor IA-2	1
242	tr|A0A067NP30|A0A067NP30_PLEOS	Peptidase S53 domain-containing protein	2
243	tr|A0A067NQW1|A0A067NQW1_PLEOS	Trehalase	3
244	tr|A0A067NKQ1|A0A067NKQ1_PLEOS	MaoC-like domain-containing protein	2
245	tr|A0A067NFD3|A0A067NFD3_PLEOS	Dipeptidyl-peptidase V	4
246	tr|A0A067P2Z7|A0A067P2Z7_PLEOS	Thioredoxin reductase	4
247	tr|A0A067P1A7|A0A067P1A7_PLEOS	SCP domain-containing protein	3
248	tr|A0A067PCE7|A0A067PCE7_PLEOS	SCP domain-containing protein	2
249	tr|A0A067NCV3|A0A067NCV3_PLEOS	PPM-type phosphatase domain-containing protein	2
250	tr|A0A067P386|A0A067P386_PLEOS	Acetylglucosamine phosphomutase	2
251	tr|B1Q4S7|B1Q4S7_PLEOS	Ribonuclease T1	4
252	sp|P81762|RNPO_PLEOS	Guanyl-specific ribonuclease Po1	4
253	tr|A0A067P6M0|A0A067P6M0_PLEOS	CYTOSOL_AP domain-containing protein	2

254	tr|A0A067NV79|A0A067NV79_PLEOS	Proteasome subunit alpha type	2
255	tr|A0A067P416|A0A067P416_PLEOS	Adenylate kinase	3
256	tr|A0A067NIC8|A0A067NIC8_PLEOS	AMP-binding domain-containing protein	2
257	tr|A0A067N9Q5|A0A067N9Q5_PLEOS	Cytochrome c oxidase subunit	2
258	tr|A0A067P286|A0A067P286_PLEOS	Succinate--CoA ligase [ADP-forming] subunit beta, mitochondrial	2
259	tr|A0A067P2R6|A0A067P2R6_PLEOS	Aminopeptidase	2
260	tr|A0A067PBZ8|A0A067PBZ8_PLEOS	Dynamin-type G domain-containing protein	1
261	tr|A0A067NF07|A0A067NF07_PLEOS	Peptidylprolyl isomerase	5
262	tr|A0A067NVM8|A0A067NVM8_PLEOS	Seryl-tRNA synthetase	2
263	tr|A0A067NTH7|A0A067NTH7_PLEOS	Peptidyl-prolyl cis-trans isomerase	1
264	tr|A0A067NL68|A0A067NL68_PLEOS	ATP synthase subunit delta, mitochondrial	2
265	tr|A0A067P9H9|A0A067P9H9_PLEOS	Phosphoglycerate mutase (2,3-diphosphoglycerate-independent)	1
266	tr|A0A067P1B7|A0A067P1B7_PLEOS	Aldo_ket_red domain-containing protein	1
267	tr|A0A067NG33|A0A067NG33_PLEOS	Glycogen [starch] synthase	2
268	tr|A0A067NPI1|A0A067NPI1_PLEOS	Epimerase domain-containing protein	1
269	tr|A0A4P8PG55|A0A4P8PG55_PLEER	Cinnamyl alcohol dehydrogenase	1
270	tr|A0A067P3D8|A0A067P3D8_PLEOS	CS domain-containing protein	1
271	tr|A0A067N2J4|A0A067N2J4_PLEOS	26S proteasome regulatory subunit RPN1	1
272	tr|A0A067NIR7|A0A067NIR7_PLEOS	Endoplasmic reticulum transmembrane protein	2
273	tr|A0A067NEB8|A0A067NEB8_PLEOS	Eukaryotic translation initiation factor 3 subunit G	1
274	tr|A0A067N928|A0A067N928_PLEOS	Thioredoxin domain-containing protein	2
275	tr|A0A067NXU3|A0A067NXU3_PLEOS	Isocitrate dehydrogenase [NADP]	1
276	tr|A0A067N6M3|A0A067N6M3_PLEOS	MFS domain-containing protein	1
277	tr|A0A067NKG1|A0A067NKG1_PLEOS	Glyco_hydro_63 domain-containing protein	1
278	tr|A0A067PCJ7|A0A067PCJ7_PLEOS	Protein kinase domain-containing protein	2
279	tr|A0A067N6C1|A0A067N6C1_PLEOS	Protein kinase domain-containing protein	2
280	tr|A0A067NG73|A0A067NG73_PLEOS	5-aminoimidazole-4-carboxamide ribonucleotide formyltransferase	1
281	tr|A0A067NSC6|A0A067NSC6_PLEOS	S-formylglutathione hydrolase	2
282	tr|A0A067P131|A0A067P131_PLEOS	Phosphotransferase	1
283	tr|A0A067NZC1|A0A067NZC1_PLEOS	Actin-related protein 2/3 complex subunit 3	1
284	tr|A0A067NXG5|A0A067NXG5_PLEOS	Polysacc_synt_4 domain-containing protein	1
285	tr|A0A067NJF4|A0A067NJF4_PLEOS	Glucosamine-6-phosphate isomerase	1
286	tr|A0A067NCR3|A0A067NCR3_PLEOS	Alkaline phosphatase	1
287	tr|A0A067NMC7|A0A067NMC7_PLEOS	Alkaline phosphatase	1
**Number**	**Accession number**	**Protein name**	**Peptide(95 %)**

288	tr|A0A067NQJ2|A0A067NQJ2_PLEOS	NAD(P)-bd_dom domain-containing protein	2
289	tr|A0A067NMY5|A0A067NMY5_PLEOS	Translationally-controlled tumor protein homolog	3
290	tr|A0A067P8H2|A0A067P8H2_PLEOS	Protein kinase domain-containing protein	1
291	tr|A0A067NQQ1|A0A067NQQ1_PLEOS	Actin-related protein 2	1
292	tr|A0A067NWK2|A0A067NWK2_PLEOS	NTP_transferase domain-containing protein	1
293	tr|A0A067NY19|A0A067NY19_PLEOS	DHHA2 domain-containing protein	2
294	tr|A0A067NLY5|A0A067NLY5_PLEOS	Ubiquitin-like modifier-activating enzyme ATG7	1
295	tr|A0A067P2I9|A0A067P2I9_PLEOS	Small nuclear ribonucleoprotein Sm D2	1
296	tr|A0A067NIU1|A0A067NIU1_PLEOS	UBC core domain-containing protein	1
297	tr|A0A067NEF9|A0A067NEF9_PLEOS	Serine/threonine-protein phosphatase	1
298	tr|A0A067NYI3|A0A067NYI3_PLEOS	Adenylosuccinate synthetase	1
299	tr|A0A067P1G9|A0A067P1G9_PLEOS	COX4 subunit of cytochrome c oxidase	1
300	tr|A0A067N250|A0A067N250_PLEOS	Aldo_ket_red domain-containing protein	4
301	tr|A0A067NZ22|A0A067NZ22_PLEOS	Iso_dh domain-containing protein	2
302	tr|A0A067P023|A0A067P023_PLEOS	Leucyl-tRNA synthetase	1
303	tr|A0A067P7P3|A0A067P7P3_PLEOS	Nascent polypeptide-associated complex subunit beta	1
304	tr|A0A067NK38|A0A067NK38_PLEOS	AAA domain-containing protein	1
305	tr|A0A067NF91|A0A067NF91_PLEOS	AAA domain-containing protein	2
306	tr|A0A067NS64|A0A067NS64_PLEOS	Amino_oxidase domain-containing protein	1
307	tr|A0A067NP65|A0A067NP65_PLEOS	Amino_oxidase domain-containing protein	1
308	tr|A0A067P0D9|A0A067P0D9_PLEOS	Amino_oxidase domain-containing protein	1
309	tr|A0A067P119|A0A067P119_PLEOS	HABP4_PAI-RBP1 domain-containing protein	1
310	tr|A0A067NZQ8|A0A067NZQ8_PLEOS	Alanine--tRNA ligase	1
311	tr|A0A067NWA0|A0A067NWA0_PLEOS	WD_REPEATS_REGION domain-containing protein	1
312	tr|A0A067NR84|A0A067NR84_PLEOS	Branched-chain-amino-acid aminotransferase	1
313	tr|A0A067NJ05|A0A067NJ05_PLEOS	Cytochrome c1, component of the mitochondrial respiratory chain	1
314	tr|A0A067NI49|A0A067NI49_PLEOS	CHZ domain-containing protein	1
315	tr|A0A067NBE6|A0A067NBE6_PLEOS	cobW domain-containing protein	1
316	tr|A0A067PDG2|A0A067PDG2_PLEOS	eRF1_1 domain-containing protein	1
317	tr|A0A067PBX9|A0A067PBX9_PLEOS	Glutamyl-tRNA synthetase	1
318	tr|A0A067PB82|A0A067PB82_PLEOS	PCI domain-containing protein	1
319	tr|A0A067P5S8|A0A067P5S8_PLEOS	Glycosyltransferase family 8 protein	1
320	tr|A0A067P4I9|A0A067P4I9_PLEOS	40S ribosomal protein S24	1

321	tr|A0A067P498|A0A067P498_PLEOS	Aminotran_1_2 domain-containing protein	1
322	tr|A0A067P3B8|A0A067P3B8_PLEOS	U6 snRNA-associated Sm-like protein LSm5	1
323	tr|A0A067P276|A0A067P276_PLEOS	40S ribosomal protein S27 (Fragment)	2
324	tr|A0A067NZG7|A0A067NZG7_PLEOS	Glucosidase 2 subunit beta	1
325	tr|A0A067NV28|A0A067NV28_PLEOS	CVNH domain-containing protein (Fragment)	1
326	tr|A0A067NU90|A0A067NU90_PLEOS	6-phosphogluconolactonase	1
327	tr|A0A067NU40|A0A067NU40_PLEOS	Proteasome subunit alpha type	1
328	tr|A0A067NT94|A0A067NT94_PLEOS	NIPSNAP domain-containing protein	1
329	tr|A0A067NT34|A0A067NT34_PLEOS	Non-specific serine/threonine protein kinase	1
330	tr|A0A067N3W2|A0A067N3W2_PLEOS	Protein kinase domain-containing protein	1
331	tr|A0A067NSS7|A0A067NSS7_PLEOS	Protein YOP1	1
332	tr|A0A067NRL6|A0A067NRL6_PLEOS	Cytochrome b5 heme-binding domain-containing protein	1
333	tr|A0A067NQW7|A0A067NQW7_PLEOS	Phospho-2-dehydro-3-deoxyheptonate aldolase	1
334	tr|A0A067NQC8|A0A067NQC8_PLEOS	Glycerol-3-phosphate dehydrogenase	1
335	tr|A0A067NP81|A0A067NP81_PLEOS	F-box domain-containing protein	1
336	tr|A0A067NNA2|A0A067NNA2_PLEOS	PKS_ER domain-containing protein	1
337	tr|A0A067NMT1|A0A067NMT1_PLEOS	DJ-1_PfpI domain-containing protein	1
338	tr|A0A067NK74|A0A067NK74_PLEOS	Ubiquitin carboxyl-terminal hydrolase	1
339	tr|A0A067NJM9|A0A067NJM9_PLEOS	Coatomer subunit beta	1
340	tr|A0A067NIZ8|A0A067NIZ8_PLEOS	Chitinase	1
341	tr|A0A067NIW9|A0A067NIW9_PLEOS	Sm domain-containing protein	1
342	tr|A0A067NE87|A0A067NE87_PLEOS	Sm domain-containing protein	1
343	tr|A0A067NGS6|A0A067NGS6_PLEOS	MFS domain-containing protein	1
344	tr|A0A067NFC6|A0A067NFC6_PLEOS	acidPPc domain-containing protein	1
345	tr|A0A067NCM1|A0A067NCM1_PLEOS	AAA domain-containing protein	1
346	RRRRRtr|A0A067NTP5|A0A067NTP5_PLEOS	REVERSED OPT superfamily	1
347	tr|A0A067NDI3|A0A067NDI3_PLEOS	RRM domain-containing protein	1
348	tr|A0A067NI38|A0A067NI38_PLEOS	Triosephosphate isomerase	4
349	tr|A0A067ND43|A0A067ND43_PLEOS	Carbonic anhydrase	1
350	tr|A0A067NGV7|A0A067NGV7_PLEOS	Prolyl-tRNA synthetase	1
351	tr|A0A067NL12|A0A067NL12_PLEOS	Carbohydrate esterase family 4 protein	2
352	tr|A0A067P2K4|A0A067P2K4_PLEOS	KOW domain-containing protein	3
353	tr|A0A067NYY5|A0A067NYY5_PLEOS	Proteasome subunit alpha type	1
354	tr|A0A067NJ32|A0A067NJ32_PLEOS	Peptidase_M43 domain-containing protein	3
355	tr|Q5Y972|Q5Y972_PLEOS	Metalloprotease	2
356	tr|A0A067PAK5|A0A067PAK5_PLEOS	Aldo_ket_red domain-containing protein	1
357	tr|A0A067NME3|A0A067NME3_PLEOS	Serine/threonine-protein phosphatase	1

358	tr|A0A067P091|A0A067P091_PLEOS	CCT-alpha	2
359	tr|A0A067NLS2|A0A067NLS2_PLEOS	Alpha-mannosidase	1
360	tr|A0A067NIX9|A0A067NIX9_PLEOS	Eukaryotic translation initiation factor 3 subunit C	1
361	tr|A0A067NV66|A0A067NV66_PLEOS	UBC core domain-containing protein	1
362	tr|A0A067NIJ5|A0A067NIJ5_PLEOS	Protein kinase domain-containing protein (Fragment)	1
363	tr|A0A067NC64|A0A067NC64_PLEOS	Valyl-tRNA synthetase	1
364	tr|A0A067ND49|A0A067ND49_PLEOS	Thioredoxin domain-containing protein	1
365	tr|A0A067NII8|A0A067NII8_PLEOS	Histone-glutamine methyltransferase	1
366	tr|A0A067PC07|A0A067PC07_PLEOS	Proteasome subunit beta	2
367	tr|A0A067NGT9|A0A067NGT9_PLEOS	D-ribose-5-phosphate ketol-isomerase	1
368	tr|A0A067NX49|A0A067NX49_PLEOS	NDUFS2, NADH ubiquinone oxidoreductase 49 kd subunit	1
369	tr|A0A067PBM8|A0A067PBM8_PLEOS	Glyoxalase II	2
370	tr|A0A067NS15|A0A067NS15_PLEOS	WD_REPEATS_REGION domain-containing protein	1
371	tr|A0A067NG50|A0A067NG50_PLEOS	Proteasome subunit beta	1
372	tr|A0A067NCR9|A0A067NCR9_PLEOS	Eukaryotic translation initiation factor 6	1
373	tr|A0A067NQ72|A0A067NQ72_PLEOS	Protein kinase domain-containing protein	1
374	tr|A0A067NUY3|A0A067NUY3_PLEOS	Lactoylglutathione lyase	1
375	tr|A0A067NF76|A0A067NF76_PLEOS	Importin N-terminal domain-containing protein	2
376	tr|A0A067P1S4|A0A067P1S4_PLEOS	Aspartate aminotransferase	1
377	tr|A0A067NQF8|A0A067NQF8_PLEOS	RPN13_C domain-containing protein	1
378	tr|A0A067NUP7|A0A067NUP7_PLEOS	Aspartate aminotransferase	1
379	tr|A0A067NLD2|A0A067NLD2_PLEOS	CRAL-TRIO domain-containing protein	1
380	tr|A0A067NKU9|A0A067NKU9_PLEOS	U6 snRNA-associated Sm-like protein LSm2	1
381	tr|A0A067NP99|A0A067NP99_PLEOS	Ubiquitin-like domain-containing protein	1
382	tr|A0A067N771|A0A067N771_PLEOS	NMO domain-containing protein (Fragment)	2
383	tr|A0A067NWM8|A0A067NWM8_PLEOS	GMC_OxRdtase_N domain-containing protein	1
384	tr|A0A067P3I4|A0A067P3I4_PLEOS	Prenylcys_lyase domain-containing protein	1
385	tr|A0A067NYB2|A0A067NYB2_PLEOS	Proteasome subunit alpha type	1
386	tr|A0A067NR93|A0A067NR93_PLEOS	DLH domain-containing protein	1
387	tr|A0A067NIQ7|A0A067NIQ7_PLEOS	Protein-synthesizing GTPase	2
388	tr|A0A067NQR8|A0A067NQR8_PLEOS	VWFA domain-containing protein	1
389	tr|A0A067NC16|A0A067NC16_PLEOS	MSP domain-containing protein	2
390	tr|A0A067P322|A0A067P322_PLEOS	AAA domain-containing protein	1
391	tr|A0A067NN60|A0A067NN60_PLEOS	26S proteasome regulatory subunit RPN11	1
392	tr|A0A067P1M5|A0A067P1M5_PLEOS	26S proteasome regulatory subunit RPN7	1

393	tr|A0A067NYN3|A0A067NYN3_PLEOS	6,7-dimethyl-8-ribityllumazine synthase	1
394	tr|A0A067PBT5|A0A067PBT5_PLEOS	Pyridoxal 5'-phosphate synthase (glutamine hydrolyzing)	1
395	tr|A0A067PC20|A0A067PC20_PLEOS	Gln-synt_C domain-containing protein	1
396	tr|A0A067NTR2|A0A067NTR2_PLEOS	Glycoside hydrolase family 13 protein	2
397	tr|A0A067NVZ2|A0A067NVZ2_PLEOS	Clathrin light chain	1
398	tr|G8DA03|G8DA03_9AGAR	Beta-flanking protein (Fragment)	1
399	tr|G8D9Z9|G8D9Z9_9AGAR	Beta-flanking protein (Fragment)	1
400	tr|A0A067NY97|A0A067NY97_PLEOS	Threonyl-tRNA synthetase	1
401	tr|A0A067NZM0|A0A067NZM0_PLEOS	PHB domain-containing protein	1
402	tr|A0A067N7V4|A0A067N7V4_PLEOS	NAD(P)-bd_dom domain-containing protein	1
403	tr|A0A2Z4PGK3|A0A2Z4PGK3_PLEOS	Alpha,alpha-trehalose-phosphate synthase (UDP-forming)	1
404	tr|A0A2S1Q3T7|A0A2S1Q3T7_9AGAR	Alpha,alpha-trehalose-phosphate synthase (UDP-forming)	1
405	tr|A0A067NCF7|A0A067NCF7_PLEOS	Alpha,alpha-trehalose-phosphate synthase (UDP-forming)	1
406	tr|A0A067NF06|A0A067NF06_PLEOS	AMP_N domain-containing protein	1
407	tr|A0A067PCL4|A0A067PCL4_PLEOS	Glutamate decarboxylase (Fragment)	1
408	tr|A0A067NIP1|A0A067NIP1_PLEOS	Histone H3	1
409	tr|A0A067P6P6|A0A067P6P6_PLEOS	Histone H3	1
410	tr|A0A067NQZ3|A0A067NQZ3_PLEOS	Histone H3	1
411	RRRRRtr|A0A067NZM5|A0A067NZM5_PLEOS	REVERSED GAR domain-containing protein	1
412	tr|A0A067NFJ2|A0A067NFJ2_PLEOS	Thiamine thiazole synthase	1
413	tr|A0A067N9Q7|A0A067N9Q7_PLEOS	NMO domain-containing protein (Fragment)	1
414	tr|A0A482GMN1|A0A482GMN1_PLEOS	Peroxidase	1
415	tr|A0A067NK95|A0A067NK95_PLEOS	Peroxidase	1
416	tr|A0A067NTZ1|A0A067NTZ1_PLEOS	Mitogen-activated protein kinase	1
417	tr|A0A067NBT8|A0A067NBT8_PLEOS	PKS_ER domain-containing protein	1
418	tr|A0A067NBT2|A0A067NBT2_PLEOS	PKS_ER domain-containing protein	1
419	RRRRRtr|A0A067NNI2|A0A067NNI2_PLEOS	REVERSED Thymidylate synthase	1
420	tr|A0A067NIF7|A0A067NIF7_PLEOS	CMP/dCMP-type deaminase domain-containing protein	1
421	tr|A0A067PA83|A0A067PA83_PLEOS	40S ribosomal protein S30	2
422	tr|A0A067NPL8|A0A067NPL8_PLEOS	CULLIN_2 domain-containing protein	1
423	tr|A0A067NRN5|A0A067NRN5_PLEOS	Importin N-terminal domain-containing protein	1
424	tr|A0A067NLH9|A0A067NLH9_PLEOS	Calcineurin regulatory subunit	1
425	tr|A0A067P0V3|A0A067P0V3_PLEOS	Protein kinase domain-containing protein	1
426	tr|A0A067NEQ6|A0A067NEQ6_PLEOS	Protein kinase domain-containing protein	1
427	tr|A0A067P591|A0A067P591_PLEOS	Histidine--tRNA ligase (Fragment)	1
428	tr|A0A067PBH5|A0A067PBH5_PLEOS	F-box domain-containing protein	1

429	tr|A0A067NUI1|A0A067NUI1_PLEOS	Acetyltransferase component of pyruvate dehydrogenase complex	0
430	tr|A0A067NRD1|A0A067NRD1_PLEOS	Cytochrome b-c1 complex subunit Rieske, mitochondrial	1
431	tr|A0A067NI97|A0A067NI97_PLEOS	HMG box domain-containing protein (Fragment)	1
432	tr|A0A067NSW7|A0A067NSW7_PLEOS	Proteasome subunit beta	2
433	tr|A0A067NXR1|A0A067NXR1_PLEOS	Peptidase M20 domain-containing protein 2	1
434	tr|A0A067P016|A0A067P016_PLEOS	Complex I-B22	1
435	tr|A0A067NS50|A0A067NS50_PLEOS	Importin subunit alpha	1
436	RRRRRtr|A0A067NGR3|A0A067NGR3_PLEOS	REVERSED DNA polymerase epsilon catalytic subunit	0
437	tr|A0A067P193|A0A067P193_PLEOS	Protein AF-9 homolog	1
438	tr|A0A067NI18|A0A067NI18_PLEOS	Delta-aminolevulinic acid dehydratase	0
439	tr|A0A067PDW6|A0A067PDW6_PLEOS	U3 small nucleolar RNA-associated protein 22	0
440	tr|A0A067NRS7|A0A067NRS7_PLEOS	ARID domain-containing protein	0
441	tr|A0A067P3C8|A0A067P3C8_PLEOS	V-type proton ATPase subunit H	0
442	tr|A0A067NJ57|A0A067NJ57_PLEOS	Adenylyl-sulfate kinase	0
443	tr|Q8X1M9|Q8X1M9_PLEOS	Pleurotolysin A	35
444	tr|T2HUL2|T2HUL2_PLEER	Pe.pleurotolysin A	32
445	tr|A0A067P4F3|A0A067P4F3_PLEOS	Aspartyl-tRNA synthetase	0
446	tr|C4PFY6|C4PFY6_PLEOS	Peptidase 1	0
447	RRRRRtr|A0A067P4L8|A0A067P4L8_PLEOS	REVERSED Dopey_N domain-containing protein	0
448	tr|A0A067N7L7|A0A067N7L7_PLEOS	Polyketide_cyc domain-containing protein	1
449	tr|A0A067PDH6|A0A067PDH6_PLEOS	GED domain-containing protein	0
450	tr|A0A067NFB0|A0A067NFB0_PLEOS	Dynein light chain	0
451	tr|A0A067NF53|A0A067NF53_PLEOS	NADH dehydrogenase [ubiquinone] iron-sulfur protein 4, mitochondrial	0
452	tr|A0A067P034|A0A067P034_PLEOS	Uridylate kinase	0
453	tr|A0A067N3J6|A0A067N3J6_PLEOS	Eukaryotic translation initiation factor 3 subunit A	0
454	tr|A0A067NH25|A0A067NH25_PLEOS	UBC core domain-containing protein	0
455	tr|A0A067ND69|A0A067ND69_PLEOS	Midasin (Fragment)	0
456	tr|A0A067NU94|A0A067NU94_PLEOS	Prohibitin	0
457	RRRRRtr|A0A6B9F1E8|A0A6B9F1E8_PLEOS	REVERSED Zinc cluster transcription factor 44 (Fragment)	0
458	RRRRRtr|A0A067NJG4|A0A067NJG4_PLEOS	REVERSED Zn(2)-C6 fungal-type domain-containing protein	0
459	tr|A0A067P5K9|A0A067P5K9_PLEOS	Lysine--tRNA ligase	1
460	tr|A0A067P1P7|A0A067P1P7_PLEOS	NDUFV2, NADH dehydrogenase 24 kd subunit	0

461	tr|A0A067NTG4|A0A067NTG4_PLEOS	Peptidase A1 domain-containing protein	1
462	RRRRRtr|A0A067NT85|A0A067NT85_PLEOS	REVERSED SMP-LTD domain-containing protein	0
463	tr|A0A067NGA5|A0A067NGA5_PLEOS	NEDD8-activating enzyme E1 regulatory subunit	0
464	tr|A0A067N9Q3|A0A067N9Q3_PLEOS	Proline iminopeptidase	0
465	tr|A0A067NDJ2|A0A067NDJ2_PLEOS	PH domain-containing protein	0
466	tr|A0A067N2Y5|A0A067N2Y5_PLEOS	MACPF domain-containing protein	0
467	tr|A0A067PBX5|A0A067PBX5_PLEOS	3-hydroxyisobutyryl-CoA hydrolase, mitochondrial	0
468	tr|A0A067NSE0|A0A067NSE0_PLEOS	PCI domain-containing protein	0
469	tr|A0A067N4K4|A0A067N4K4_PLEOS	Proteasome subunit beta	0
470	RRRRRtr|A0A067NFR2|A0A067NFR2_PLEOS	REVERSED NMO domain-containing protein	0
471	tr|A0A067NDT8|A0A067NDT8_PLEOS	GDP-Man:Man(3)GlcNAc(2)-PP-Dol alpha-1,2-mannosyltransferase	0
472	tr|A0A067NHD2|A0A067NHD2_PLEOS	Diadenosine tetraphosphate synthetase	0
473	tr|A0A067PCH4|A0A067PCH4_PLEOS	Actin cytoskeleton-regulatory complex protein PAN1	0
474	tr|A0A067P638|A0A067P638_PLEOS	U6 snRNA-associated Sm-like protein LSm8	0
475	tr|A0A067NGV9|A0A067NGV9_PLEOS	Phosphatidylinositol transfer protein SFH5	0
476	tr|A0A067NAR2|A0A067NAR2_PLEOS	3-hydroxyacyl-[acyl-carrier-protein] dehydratase	0
477	tr|A0A067NMD2|A0A067NMD2_PLEOS	CTP synthase	0
478	tr|A0A067NR04|A0A067NR04_PLEOS	Patatin-like phospholipase domain-containing protein	0
479	RRRRRtr|A0A067NPT5|A0A067NPT5_PLEOS	REVERSED Peptidase_S9 domain-containing protein	0
480	tr|A0A067NPZ8|A0A067NPZ8_PLEOS	GMC_oxred_C domain-containing protein	0
481	tr|A0A067PAL3|A0A067PAL3_PLEOS	DNA-(apurinic or apyrimidinic site) endonuclease	0
482	RRRRRtr|A0A067NFU2|A0A067NFU2_PLEOS	REVERSED Structural maintenance of chromosomes protein	0
483	tr|A0A067NQU6|A0A067NQU6_PLEOS	PKS_ER domain-containing protein	0
484	tr|A0A067NMV8|A0A067NMV8_PLEOS	Asparaginyl-tRNA synthetase	0
485	tr|A0A067NKJ0|A0A067NKJ0_PLEOS	Diphosphomevalonate decarboxylase	0
486	tr|A0A067NXW5|A0A067NXW5_PLEOS	ERF-3	0
487	tr|A0A067NRV8|A0A067NRV8_PLEOS	Aspartate-semialdehyde dehydrogenase	0
488	tr|A0A067NT86|A0A067NT86_PLEOS	Sorting nexin-3	0
489	tr|A0A067NIZ1|A0A067NIZ1_PLEOS	Importin N-terminal domain-containing protein	0
490	tr|A0A067NLR9|A0A067NLR9_PLEOS	Homocitrate synthase	0

491	tr|A0A067NYQ3|A0A067NYQ3_PLEOS	Methylenetetrahydrofolate reductase	0
492	tr|A0A067NH04|A0A067NH04_PLEOS	Profilin	0
493	RRRRRtr|A0A067NKD1|A0A067NKD1_PLEOS	REVERSED Non-ribosomal peptide synthetase	1
494	RRRRRtr|A0A067NI85|A0A067NI85_PLEOS	REVERSED F-box domain-containing protein	0
495	tr|A0A067NL86|A0A067NL86_PLEOS	CCT-beta	0
496	tr|O14445|O14445_PLEOS	Succinate dehydrogenase [ubiquinone] iron-sulfur subunit, mitochondrial	0
497	tr|A0A067N939|A0A067N939_PLEOS	Succinate dehydrogenase [ubiquinone] iron-sulfur subunit, mitochondrial	0
498	tr|A0A067P7D0|A0A067P7D0_PLEOS	Reverse transcriptase domain-containing protein (Fragment)	0
499	tr|A0A067P6D9|A0A067P6D9_PLEOS	PSDC domain-containing protein	0
500	tr|A0A067NSH9|A0A067NSH9_PLEOS	CS domain-containing protein (Fragment)	0
501	tr|A0A067NRP5|A0A067NRP5_PLEOS	Arp2/3 complex 34 kDa subunit	0
502	RRRRRtr|A0A067NKD0|A0A067NKD0_PLEOS	REVERSED CCHC-type domain-containing protein	0
503	RRRRRtr|A0A067NT01|A0A067NT01_PLEOS	REVERSED CCHC-type domain-containing protein	0
504	RRRRRtr|A0A067NLS4|A0A067NLS4_PLEOS	REVERSED CCHC-type domain-containing protein	0
505	RRRRRtr|A0A067NMX8|A0A067NMX8_PLEOS	REVERSED CCHC-type domain-containing protein	0
506	RRRRRtr|A0A067N8X4|A0A067N8X4_PLEOS	REVERSED CCHC-type domain-containing protein	0
507	RRRRRtr|A0A067NDD6|A0A067NDD6_PLEOS	REVERSED CCHC-type domain-containing protein	0
508	RRRRRtr|A0A067P0Y1|A0A067P0Y1_PLEOS	REVERSED CCHC-type domain-containing protein	0
509	RRRRRtr|A0A067NLJ0|A0A067NLJ0_PLEOS	REVERSED CCHC-type domain-containing protein	0
510	RRRRRtr|A0A067N3A3|A0A067N3A3_PLEOS	REVERSED CCHC-type domain-containing protein	0
511	tr|A0A067NKY0|A0A067NKY0_PLEOS	EXS domain-containing protein	0
512	RRRRRtr|A0A067P599|A0A067P599_PLEOS	REVERSED DUF2235 domain-containing protein	0
513	tr|A0A067NTZ2|A0A067NTZ2_PLEOS	GP-PDE domain-containing protein	0
514	RRRRRtr|A0A067NVP4|A0A067NVP4_PLEOS	REVERSED YccV-like domain-containing protein	0
515	RRRRRtr|A0A067NNJ5|A0A067NNJ5_PLEOS	REVERSED SAC domain-containing protein	0
516	RRRRRtr|A0A067NED5|A0A067NED5_PLEOS	REVERSED SHSP domain-containing protein	0

1	tr|A0A067NUP1|A0A067NUP1_PLEOS	Elongation factor 1-alpha	42

517	RRRRRtr|Q5GHF1|Q5GHF1_9VIRU	REVERSED RNA-dependent RNA polymerase	0
518	tr|A0A067P1F8|A0A067P1F8_PLEOS	Dolichyl-diphosphooligosaccharide--protein glycosyltransferase subunit WBP1	0
519	RRRRRtr|A0A067P4F5|A0A067P4F5_PLEOS	REVERSED 1-phosphatidylinositol-3-phosphate 5-kinase	0
520	tr|A7KCT0|A7KCT0_PLEOS	Cytochrome c oxidase subunit 2	0
521	tr|A0A344LZ57|A0A344LZ57_PLECO	Cytochrome c oxidase subunit 2	0
522	tr|A0A343AWQ7|A0A343AWQ7_PLEER	Cytochrome c oxidase subunit 2	0
523	tr|A0A2U8XDF0|A0A2U8XDF0_PLEPU	Cytochrome c oxidase subunit 2	0
524	tr|A0A2K9YPH9|A0A2K9YPH9_9AGAR	Cytochrome c oxidase subunit 2	0
525	tr|A0A067NV80|A0A067NV80_PLEOS	FK506-binding protein	0
526	RRRRRtr|A0A067NGQ5|A0A067NGQ5_PLEOS	REVERSED F-box domain-containing protein	0
527	tr|A0A067NUJ5|A0A067NUJ5_PLEOS	Glycosyltransferase family 20 protein	0
528	tr|A0A067NAC8|A0A067NAC8_PLEOS	QCR2, subunit of the ubiquinol cytochrome-c reductase complex	0
529	RRRRRtr|A0A067P9G0|A0A067P9G0_PLEOS	REVERSED Pkinase_fungal domain-containing protein	0
530	tr|A0A067NFY1|A0A067NFY1_PLEOS	DNA polymerase	0
531	tr|A0A067PCR2|A0A067PCR2_PLEOS	Cnn_1N domain-containing protein	0
532	RRRRRtr|A0A067P8P1|A0A067P8P1_PLEOS	REVERSED HNHc domain-containing protein	0
533	RRRRRtr|A0A067NV50|A0A067NV50_PLEOS	REVERSED Serine/threonine-protein kinase	0
534	RRRRRtr|A0A067NJG9|A0A067NJG9_PLEOS	REVERSED zf-C2HC5 domain-containing protein	0
535	tr|A0A067NJB2|A0A067NJB2_PLEOS	Jacalin-type lectin domain-containing protein	0
536	tr|A0A067NZF0|A0A067NZF0_PLEOS	Dipeptidyl peptidase 3	0
537	tr|A0A067NVI9|A0A067NVI9_PLEOS	ADF-H domain-containing protein	0
538	tr|A0A067NTW8|A0A067NTW8_PLEOS	DNA helicase	0
539	tr|A0A067PCU8|A0A067PCU8_PLEOS	acidPPc domain-containing protein	0
540	tr|A0A067P9P4|A0A067P9P4_PLEOS	Proliferating cell nuclear antigen	0
541	tr|A0A067NL09|A0A067NL09_PLEOS	MPN domain-containing protein	0

2	tr|A0A067NEB2|A0A067NEB2_PLEOS	Aldo_ket_red domain-containing protein	38
3	tr|A0A067P9U4|A0A067P9U4_PLEOS	HATPase_c domain-containing protein	24
4	tr|A0A067P0J4|A0A067P0J4_PLEOS	5-methyltetrahydropteroyltriglutamate--homocysteine S-methyltransferase	22
5	tr|A0A067P9S7|A0A067P9S7_PLEOS	Glycosyltransferase family 4 protein	18
6	tr|A0A067NW08|A0A067NW08_PLEOS	Elongation factor 2	20
7	tr|A0A067P4U9|A0A067P4U9_PLEOS	Phosphopyruvate hydratase	23
8	tr|A0A067P303|A0A067P303_PLEOS	6-phosphogluconate dehydrogenase, decarboxylating	16

9	tr|A0A067N8U4|A0A067N8U4_PLEOS	6-phosphogluconate dehydrogenase, decarboxylating	16
10	tr|A0A482GPW1|A0A482GPW1_PLEOS	Catalase	17
11	tr|A0A2H4UZK7|A0A2H4UZK7_PLEOS	Catalase (Fragment)	17
12	tr|A0A067NHY5|A0A067NHY5_PLEOS	Catalase	17
13	tr|A0A067NRP1|A0A067NRP1_PLEOS	14_3_3 domain-containing protein	16
14	tr|A0A067NLR3|A0A067NLR3_PLEOS	ATP-citrate synthase	9
15	tr|A0A067NV82|A0A067NV82_PLEOS	Pyruvate kinase	10
16	tr|A0A067NJB6|A0A067NJB6_PLEOS	Carbohydrate-binding module family 12 protein	10
17	tr|A0A067NCW7|A0A067NCW7_PLEOS	Ostreolysin-like protein	17
18	sp|P83467|OLYA6_PLEOS	Ostreolysin A6	17
19	tr|A0A067N8D2|A0A067N8D2_PLEOS	RRM domain-containing protein	14
20	tr|A0A067NYL0|A0A067NYL0_PLEOS	UDP-glucose 6-dehydrogenase	11
21	tr|A0A067N792|A0A067N792_PLEOS	Endoplasmic reticulum chaperone BiP	11
22	tr|A0A067NS53|A0A067NS53_PLEOS	Pyruvate carboxylase	8
23	tr|A0A067NGR8|A0A067NGR8_PLEOS	Ribos_L4_asso_C domain-containing protein	9
24	tr|A0A067NVZ7|A0A067NVZ7_PLEOS	D-fructose-6-phosphate amidotransferase	7
25	tr|A0A067N302|A0A067N302_PLEOS	Adenosylhomocysteinase	10
26	tr|U6A6W7|U6A6W7_PLEOS	Aspartic protease	8
27	tr|A0A067NMW5|A0A067NMW5_PLEOS	Peptidase A1 domain-containing protein (Fragment)	8
28	tr|A0A067N9N5|A0A067N9N5_PLEOS	Alpha-1,4 glucan phosphorylase	14
29	tr|A0A067NTI0|A0A067NTI0_PLEOS	ATP synthase subunit alpha	9
30	tr|A0A067NI01|A0A067NI01_PLEOS	UTP--glucose-1-phosphate uridylyltransferase	8
31	tr|A0A067NZL3|A0A067NZL3_PLEOS	Transaldolase	7
32	tr|A0A067N929|A0A067N929_PLEOS	Ketol-acid reductoisomerase, mitochondrial	7
33	tr|A0A067NIF5|A0A067NIF5_PLEOS	Ribosomal_S7 domain-containing protein	8
34	tr|A0A067NR75|A0A067NR75_PLEOS	Ubiquitin-like domain-containing protein	12
35	tr|Q96TW1|Q96TW1_PLEOS	Putative ubiquitin (Fragment)	11
36	tr|A0A067NZF5|A0A067NZF5_PLEOS	Ubiquitin-like domain-containing protein	11
37	tr|A0A067NZG5|A0A067NZG5_PLEOS	40S ribosomal protein S7	9
38	tr|A0A067NII7|A0A067NII7_PLEOS	Formate dehydrogenase	8
39	tr|D2JY80|D2JY80_PLEOS	RRM domain-containing protein	9
40	tr|A0A067NP04|A0A067NP04_PLEOS	RRM domain-containing protein (Fragment)	8
41	tr|A0A067PB97|A0A067PB97_PLEOS	Phosphoglycerate kinase	8
42	sp|Q92268|TBB_PLESA	Tubulin beta chain	10
43	tr|Q9UWF3|Q9UWF3_PLESA	Tubulin beta chain	11
44	tr|A0A067N725|A0A067N725_PLEOS	Tubulin beta chain	11

45	tr|A0A067P101|A0A067P101_PLEOS	Ribosomal_L2_C domain-containing protein	4
46	sp|O94739|CALM_PLEOS	Calmodulin	6
47	sp|P11120|CALM_PLECO	Calmodulin	5
48	tr|A0A067NXS6|A0A067NXS6_PLEOS	Glucose-6-phosphate 1-dehydrogenase	4
49	tr|A0A067NS51|A0A067NS51_PLEOS	KH type-2 domain-containing protein	4
50	tr|A0A067NHG1|A0A067NHG1_PLEOS	Aldedh domain-containing protein	5
51	tr|A0A067NI86|A0A067NI86_PLEOS	Carbohydrate-binding module family 13 protein	12
52	tr|G9MD63|G9MD63_PLEOS	Glyceraldehyde-3-phosphate dehydrogenase	11
53	tr|A0A067NKG0|A0A067NKG0_PLEOS	Glyceraldehyde-3-phosphate dehydrogenase	11
54	sp|Q9UW96|G3P_PLESA	Glyceraldehyde-3-phosphate dehydrogenase	11
55	tr|H1AFL8|H1AFL8_PLEOS	Glyceraldehyde-3-phosphate dehydrogenase	10
56	tr|H1AFL5|H1AFL5_PLEOS	Glyceraldehyde-3-phosphate dehydrogenase	10
57	tr|A0A067NJ24|A0A067NJ24_PLEOS	Glyceraldehyde-3-phosphate dehydrogenase	10
58	tr|A0A067NG95|A0A067NG95_PLEOS	Dipeptidyl-peptidase V	4
59	tr|A0A067N5V4|A0A067N5V4_PLEOS	Hemerythrin domain-containing protein	7
60	tr|A0A067P657|A0A067P657_PLEOS	Aldo_ket_red domain-containing protein	5
61	tr|D2JY84|D2JY84_PLEOS	Aldo_ket_red domain-containing protein	4
62	tr|A0A067NHZ5|A0A067NHZ5_PLEOS	40S ribosomal protein S8	5
63	tr|A0A067NT84|A0A067NT84_PLEOS	Sugar phosphate phosphatase	5
64	tr|A0A067NL73|A0A067NL73_PLEOS	Malate dehydrogenase	6
65	tr|A0A067P6E3|A0A067P6E3_PLEOS	Histone H2B	6
66	tr|A0A067NZ73|A0A067NZ73_PLEOS	Histone H2B	6
67	tr|A0A067NVC3|A0A067NVC3_PLEOS	Histone H2B	6
68	tr|A0A067NSQ2|A0A067NSQ2_PLEOS	Inorganic diphosphatase	6
69	tr|A0A067NQG0|A0A067NQG0_PLEOS	40S ribosomal protein S1	6
70	tr|D2JY77|D2JY77_PLEOS	40S ribosomal protein S1	5
71	tr|A0A067NP23|A0A067NP23_PLEOS	Pleurotolysin B	6
72	tr|W8SJ43|W8SJ43_PLEER	Glutamine synthetase	8
73	tr|A0A2R8G1D5|A0A2R8G1D5_PLEOS	Glutamine synthetase	8
74	tr|A0A067NQW2|A0A067NQW2_PLEOS	Glutamine synthetase	8
75	tr|A0A067NR11|A0A067NR11_PLEOS	Protein disulfide-isomerase	5
76	tr|A0A067NEK4|A0A067NEK4_PLEOS	Peptidyl-prolyl cis-trans isomerase	16
77	tr|Q96TV2|Q96TV2_PLEOS	Peptidyl-prolyl cis-trans isomerase (Fragment)	13
78	tr|A0A067NCR3|A0A067NCR3_PLEOS	Alkaline phosphatase	4
79	tr|A0A067P0D8|A0A067P0D8_PLEOS	H(+)-transporting two-sector ATPase	5
80	tr|A0A067NL37|A0A067NL37_PLEOS	Aconitate hydratase, mitochondrial	3

81	tr|A0A067NYA3|A0A067NYA3_PLEOS	60S ribosomal protein L20	3
82	tr|A0A067NIX1|A0A067NIX1_PLEOS	Malate dehydrogenase	6
83	tr|A0A067NQG3|A0A067NQG3_PLEOS	Mitochondrial intermediate peptidase	3
84	tr|D2JY85|D2JY85_PLEOS	Histone H2A	6
85	tr|A0A067NVU4|A0A067NVU4_PLEOS	Histone H2A (Fragment)	6
86	tr|A0A067P7W0|A0A067P7W0_PLEOS	Histone H2A	5
87	tr|A0A067NY26|A0A067NY26_PLEOS	Histone H2A (Fragment)	5
88	tr|A0A067NG34|A0A067NG34_PLEOS	Histone H2A (Fragment)	5
89	tr|A0A067P305|A0A067P305_PLEOS	Transketolase	3
90	tr|A0A067NS47|A0A067NS47_PLEOS	Septin-type G domain-containing protein	4
91	tr|A0A067P0R2|A0A067P0R2_PLEOS	Tubulin alpha chain	5
92	tr|A0A067NT90|A0A067NT90_PLEOS	Citrulline--aspartate ligase	3
93	tr|A0A067P3U3|A0A067P3U3_PLEOS	Aldo_ket_red domain-containing protein	3
94	tr|A0A067NG45|A0A067NG45_PLEOS	Glycosyltransferase family 69 protein	8
95	tr|A0A067N544|A0A067N544_PLEOS	PKS_ER domain-containing protein	5
96	tr|A0A067P7Q0|A0A067P7Q0_PLEOS	Ribosomal_L18e/L15P domain-containing protein	3
97	tr|A0A067NYA5|A0A067NYA5_PLEOS	Carbohydrate-binding module family 13 protein	4
98	tr|A0A067NU68|A0A067NU68_PLEOS	Thioredoxin	3
99	tr|A0A067NTH7|A0A067NTH7_PLEOS	Peptidyl-prolyl cis-trans isomerase	3
100	tr|A0A067NED1|A0A067NED1_PLEOS	Coronin	3
101	tr|A0A067NAM1|A0A067NAM1_PLEOS	Aldo_ket_red domain-containing protein	3
102	tr|A0A067NEN6|A0A067NEN6_PLEOS	Core subunit of the ubiquinol-cytochrome c reductase complex, QCR1	3
103	tr|A0A067NMU9|A0A067NMU9_PLEOS	60S ribosomal protein L36	4
104	tr|A0A067NEY2|A0A067NEY2_PLEOS	60S ribosomal protein L8 (Fragment)	4
105	tr|A0A067PAP3|A0A067PAP3_PLEOS	PKS_ER domain-containing protein	3
106	tr|A0A067NKI6|A0A067NKI6_PLEOS	Eukaryotic translation initiation factor 5A	3
107	tr|A0A067NBU9|A0A067NBU9_PLEOS	NAD-specific glutamate dehydrogenase	3
108	tr|A0A067NV87|A0A067NV87_PLEOS	Glucose-6-phosphate isomerase	4
109	tr|A0A067P0M5|A0A067P0M5_PLEOS	Ribosomal_L18_c domain-containing protein (Fragment)	3
110	tr|A0A067NB23|A0A067NB23_PLEOS	Extracellular metalloproteinase	8
111	tr|A0A067PBA8|A0A067PBA8_PLEOS	Nucleoside diphosphate kinase	5
112	tr|A0A067NFT1|A0A067NFT1_PLEOS	40S ribosomal protein S4	2
113	tr|A0A067NLD9|A0A067NLD9_PLEOS	Transket_pyr domain-containing protein	2
114	tr|C4PFY6|C4PFY6_PLEOS	Peptidase 1	5
115	tr|A0A067NKH2|A0A067NKH2_PLEOS	Aldo_ket_red domain-containing protein	4
116	tr|A0A067NZS0|A0A067NZS0_PLEOS	ATP synthase subunit beta	8
117	tr|A0A067P077|A0A067P077_PLEOS	KOW domain-containing protein (Fragment)	3
118	tr|A0A067NQW1|A0A067NQW1_PLEOS	Trehalase	3
119	tr|A0A067NKK5|A0A067NKK5_PLEOS	CCT-theta	4

120	tr|A0A067NIB4|A0A067NIB4_PLEOS	DLH domain-containing protein	2
121	tr|A0A067NUU3|A0A067NUU3_PLEOS	40S ribosomal protein S6	3
122	tr|A0A067N4M3|A0A067N4M3_PLEOS	Thioredoxin domain-containing protein	4
123	tr|A0A067NGA1|A0A067NGA1_PLEOS	Thioredoxin domain-containing protein	3
124	tr|A0A067N4L6|A0A067N4L6_PLEOS	Thioredoxin domain-containing protein	3
125	tr|A0A067P2E6|A0A067P2E6_PLEOS	Septin-type G domain-containing protein	2
126	tr|A0A067N9G3|A0A067N9G3_PLEOS	Cytochrome b5 heme-binding domain-containing protein	4
127	tr|A0A067NTR2|A0A067NTR2_PLEOS	Glycoside hydrolase family 13 protein	3
128	tr|A0A067P056|A0A067P056_PLEOS	Aldedh domain-containing protein	2
129	tr|A0A067NKF3|A0A067NKF3_PLEOS	Citrate synthase	3
130	tr|A0A067NLX2|A0A067NLX2_PLEOS	Cytochrome b5 heme-binding domain-containing protein	3
131	tr|A0A067N2Q3|A0A067N2Q3_PLEOS	Flavodoxin-like domain-containing protein	3
132	tr|D2JY75|D2JY75_PLEOS	Flavodoxin-like domain-containing protein	3
133	tr|A0A067NPE8|A0A067NPE8_PLEOS	40S ribosomal protein S12	3
134	tr|A0A067NSH1|A0A067NSH1_PLEOS	DUF2235 domain-containing protein	2
135	tr|A0A067NUF2|A0A067NUF2_PLEOS	Ribosomal_S13_N domain-containing protein	2
136	tr|A0A067NBS5|A0A067NBS5_PLEOS	ACB domain-containing protein	2
137	tr|A0A067NXY7|A0A067NXY7_PLEOS	60S ribosomal protein L27	3
138	tr|Q96TV5|Q96TV5_PLEOS	Putative ribosomal protein S19 (Fragment)	2
139	tr|A0A067NZD8|A0A067NZD8_PLEOS	Small COPII coat GTPase SAR1	2
140	tr|A0A067P1S4|A0A067P1S4_PLEOS	Aspartate aminotransferase	3
141	tr|A0A067P007|A0A067P007_PLEOS	PABS domain-containing protein	2
142	tr|D7GKX8|D7GKX8_PLEOS	Spermidine synthase-saccharopine dehydrogenase (Fragment)	1
143	tr|A0A067NY20|A0A067NY20_PLEOS	S-(hydroxymethyl)glutathione dehydrogenase	2
144	tr|A0A067PBC2|A0A067PBC2_PLEOS	AAA domain-containing protein	2
145	tr|A0A067PA05|A0A067PA05_PLEOS	Succinate dehydrogenase [ubiquinone] flavoprotein subunit, mitochondrial	2
146	tr|A0A067P4P1|A0A067P4P1_PLEOS	Ribosomal_S17_N domain-containing protein	2
147	tr|A0A067NM16|A0A067NM16_PLEOS	60S acidic ribosomal protein P0	3
148	tr|A0A067NJ33|A0A067NJ33_PLEOS	Eukaryotic translation initiation factor 3 subunit E	2
149	tr|A0A067NET3|A0A067NET3_PLEOS	HIT domain protein	2
150	tr|A0A067NAX7|A0A067NAX7_PLEOS	Inosine-5'-monophosphate dehydrogenase	2
151	tr|A0A067P3W4|A0A067P3W4_PLEOS	Histone H4	2
152	tr|A0A067NTA7|A0A067NTA7_PLEOS	Histone H4	2
153	tr|A0A067NZJ1|A0A067NZJ1_PLEOS	Proteasome subunit alpha type	2
154	tr|A0A067NHP6|A0A067NHP6_PLEOS	40S ribosomal protein S0	8

155	tr|A0A067NPJ8|A0A067NPJ8_PLEOS	Alpha-amylase	4
156	tr|A0A067NZR1|A0A067NZR1_PLEOS	Alpha-amylase	1
157	tr|A0A067P9A6|A0A067P9A6_PLEOS	Alpha-amylase	1
158	tr|A0A067NQJ2|A0A067NQJ2_PLEOS	NAD(P)-bd_dom domain-containing protein	4
159	tr|A0A067NWN7|A0A067NWN7_PLEOS	Actin-related protein 2/3 complex subunit 4	2
160	tr|A0A067NVZ8|A0A067NVZ8_PLEOS	BTB domain-containing protein (Fragment)	2
161	tr|A0A067NYZ2|A0A067NYZ2_PLEOS	Polyadenylate-binding protein	3
162	tr|A0A067NJM9|A0A067NJM9_PLEOS	Coatomer subunit beta	2
163	tr|D2JY73|D2JY73_PLEOS	Ribosomal_L16 domain-containing protein	4
164	tr|A0A067NTH6|A0A067NTH6_PLEOS	Ribosomal_L16 domain-containing protein	4
165	tr|A0A067NUQ4|A0A067NUQ4_PLEOS	UV excision repair protein RAD23	3
166	tr|A0A067PC25|A0A067PC25_PLEOS	E3 ubiquitin ligase complex SCF subunit	3
167	tr|A0A067N9I3|A0A067N9I3_PLEOS	Rieske domain-containing protein	2
168	tr|D2JY79|D2JY79_PLEOS	Ribosomal protein L15	2
169	tr|A0A067P301|A0A067P301_PLEOS	Ribosomal protein L15	2
170	tr|A0A067NZB1|A0A067NZB1_PLEOS	S-adenosylmethionine synthase	1
171	tr|A0A067NPY7|A0A067NPY7_PLEOS	RanBD1 domain-containing protein	2
172	tr|A0A067P4S5|A0A067P4S5_PLEOS	60S ribosomal protein L13	2
173	tr|A0A067NDT5|A0A067NDT5_PLEOS	Proteasome endopeptidase complex	1
174	tr|A0A067NYE3|A0A067NYE3_PLEOS	Superoxide dismutase	2
175	tr|A0A067NYE7|A0A067NYE7_PLEOS	Peptide hydrolase	3
176	tr|A0A067NUA5|A0A067NUA5_PLEOS	S4 RNA-binding domain-containing protein	2
177	tr|A0A067NWB7|A0A067NWB7_PLEOS	RRM domain-containing protein	10
178	tr|A0A067NF95|A0A067NF95_PLEOS	Septin-type G domain-containing protein	1
179	tr|A0A067NS58|A0A067NS58_PLEOS	Clathrin heavy chain	2
180	tr|A0A067NKG1|A0A067NKG1_PLEOS	Glyco_hydro_63 domain-containing protein	1
181	tr|A0A067PAP5|A0A067PAP5_PLEOS	GTP-binding nuclear protein	3
182	tr|A0A067NZC1|A0A067NZC1_PLEOS	Actin-related protein 2/3 complex subunit 3	1
183	tr|A0A067NI49|A0A067NI49_PLEOS	CHZ domain-containing protein	1
184	tr|A0A067P2K4|A0A067P2K4_PLEOS	KOW domain-containing protein	1
185	tr|A0A067NWR7|A0A067NWR7_PLEOS	NDUFS1 subunit, NADH-ubiquinone oxidoreductase 75kD	1
186	tr|A0A067NBT8|A0A067NBT8_PLEOS	PKS_ER domain-containing protein	1
187	tr|A0A067NBT2|A0A067NBT2_PLEOS	PKS_ER domain-containing protein	1
188	tr|A0A067PBZ8|A0A067PBZ8_PLEOS	Dynamin-type G domain-containing protein	1
189	tr|A0A067N7L7|A0A067N7L7_PLEOS	Polyketide_cyc domain-containing protein	1
190	tr|A0A067NQ72|A0A067NQ72_PLEOS	Protein kinase domain-containing protein	1

191	tr|A0A067NCV7|A0A067NCV7_PLEOS	Vacuolar proton pump subunit B	2
192	tr|A0A067NKQ1|A0A067NKQ1_PLEOS	MaoC-like domain-containing protein	1
193	tr|A0A067N8V5|A0A067N8V5_PLEOS	Peptidase_M24 domain-containing protein	1
194	tr|A0A067NRY6|A0A067NRY6_PLEOS	Phosphatidylglycerol/phosphatidylinositol transfer protein	1
195	tr|A0A067P5E0|A0A067P5E0_PLEOS	COX5A, subunit of cytochrome c oxidase	2
196	tr|A0A067NS50|A0A067NS50_PLEOS	Importin subunit alpha	4
197	tr|A0A067NNJ9|A0A067NNJ9_PLEOS	1,4-alpha-glucan-branching enzyme (Fragment)	2
198	tr|A0A067NIP1|A0A067NIP1_PLEOS	Histone H3	1
199	tr|A0A067P6P6|A0A067P6P6_PLEOS	Histone H3	1
200	tr|A0A067NQZ3|A0A067NQZ3_PLEOS	Histone H3	1
201	tr|A0A067NER0|A0A067NER0_PLEOS	Tubulin alpha chain	4
202	tr|A0A067NV95|A0A067NV95_PLEOS	LCCL domain-containing protein	1
203	tr|A0A067NU35|A0A067NU35_PLEOS	T-complex protein 1 subunit gamma	2
204	tr|A0A067P9L7|A0A067P9L7_PLEOS	E3 ubiquitin-protein ligase PEP5	1
205	tr|A0A067P098|A0A067P098_PLEOS	Malic enzyme	1
206	tr|A0A067NDL4|A0A067NDL4_PLEOS	Actin-depolymerizing factor 1	2
207	tr|B1Q4S7|B1Q4S7_PLEOS	Ribonuclease T1	3
208	sp|P81762|RNPO_PLEOS	Guanyl-specific ribonuclease Po1	3
209	tr|A0A067PCH8|A0A067PCH8_PLEOS	Aldo_ket_red domain-containing protein	1
210	tr|A0A067NNA2|A0A067NNA2_PLEOS	PKS_ER domain-containing protein	1
211	tr|A0A067N6C1|A0A067N6C1_PLEOS	Protein kinase domain-containing protein	1
212	tr|A0A067PCJ7|A0A067PCJ7_PLEOS	Protein kinase domain-containing protein	1
213	tr|A0A6N0GTP5|A0A6N0GTP5_9AGAR	Gdi1 protein (Fragment)	2
214	tr|A0A6N0GTP3|A0A6N0GTP3_9AGAR	Gdi1 protein (Fragment)	2
215	tr|A0A6N0GTN9|A0A6N0GTN9_9AGAR	Gdi1 protein (Fragment)	2
216	tr|A0A6N0GTJ8|A0A6N0GTJ8_9AGAR	Gdi1 protein (Fragment)	2
217	tr|A0A6N0GTI9|A0A6N0GTI9_9AGAR	Gdi1 protein (Fragment)	2
218	tr|A0A6N0GTI4|A0A6N0GTI4_9AGAR	Gdi1 protein (Fragment)	2
219	tr|A0A6N0GTI3|A0A6N0GTI3_9AGAR	Gdi1 protein (Fragment)	2
220	tr|A0A6N0GTH0|A0A6N0GTH0_PLEER	Gdi1 protein (Fragment)	2
221	tr|A0A6N0GTG0|A0A6N0GTG0_PLEER	Gdi1 protein (Fragment)	2
222	tr|A0A6N0GTF9|A0A6N0GTF9_PLEPU	Gdi1 protein (Fragment)	2
223	tr|A0A6N0GTF7|A0A6N0GTF7_9AGAR	Gdi1 protein (Fragment)	2
224	tr|A0A6N0GTF5|A0A6N0GTF5_9AGAR	Gdi1 protein (Fragment)	2
225	tr|A0A6N0GTF0|A0A6N0GTF0_9AGAR	Gdi1 protein (Fragment)	2
226	tr|A0A6N0GTE2|A0A6N0GTE2_9AGAR	Gdi1 protein (Fragment)	2
227	tr|A0A6N0GTD9|A0A6N0GTD9_9AGAR	Gdi1 protein (Fragment)	2
228	tr|A0A6N0GTD8|A0A6N0GTD8_9AGAR	Gdi1 protein (Fragment)	2
229	tr|A0A6N0GTD6|A0A6N0GTD6_PLEER	Gdi1 protein (Fragment)	2
230	tr|A0A6N0GTD0|A0A6N0GTD0_PLEOS	Gdi1 protein (Fragment)	2
231	tr|A0A6N0GTC5|A0A6N0GTC5_PLEER	Gdi1 protein (Fragment)	2
232	tr|A0A6N0GTC3|A0A6N0GTC3_9AGAR	Gdi1 protein (Fragment)	2

233	tr∣A0A067NSA9∣A0A067NSA9_PLEOS	Rab GDP dissociation inhibitor	2
234	tr∣A0A6N0GTQ4∣A0A6N0GTQ4_9AGAR	Gdi1 protein (Fragment)	1
235	tr∣A0A6N0GTK1∣A0A6N0GTK1_PLECI	Gdi1 protein (Fragment)	1
236	tr∣A0A6N0GTG1∣A0A6N0GTG1_9AGAR	Gdi1 protein (Fragment)	1
237	tr∣A0A6N0GTE6∣A0A6N0GTE6_PLECI	Gdi1 protein (Fragment)	1
238	tr∣A0A6N0GTE3∣A0A6N0GTE3_9AGAR	Gdi1 protein (Fragment)	1
239	tr∣A0A6N0GTE1∣A0A6N0GTE1_9AGAR	Gdi1 protein (Fragment)	1
240	tr∣A0A067NV80∣A0A067NV80_PLEOS	FK506-binding protein	2
241	tr∣A0A387LBC4∣A0A387LBC4_PLEOS	Manganese lipoxygenase	2
242	tr∣A0A067N2A8∣A0A067N2A8_PLEOS	Manganese lipoxygenase	2
243	tr∣D4Q9Z3∣D4Q9Z3_PLEOS	Manganese lipoxygenase	2
244	tr∣A0A067P276∣A0A067P276_PLEOS	40S ribosomal protein S27 (Fragment)	2
245	tr∣A0A067P1G9∣A0A067P1G9_PLEOS	COX4 subunit of cytochrome c oxidase	1
246	tr∣A0A067NX49∣A0A067NX49_PLEOS	NDUFS2, NADH ubiquinone oxidoreductase 49 kd subunit	2
247	tr∣A0A067NIZ7∣A0A067NIZ7_PLEOS	Alanine--glyoxylate aminotransferase	1
248	tr∣A0A067NV66∣A0A067NV66_PLEOS	UBC core domain-containing protein	1
249	tr∣A0A067NQ82∣A0A067NQ82_PLEOS	V-type proton ATPase subunit	1
250	tr∣A0A067NIZ1∣A0A067NIZ1_PLEOS	Importin N-terminal domain-containing protein	2
251	tr∣A0A067NMW1∣A0A067NMW1_PLEOS	S10_plectin domain-containing protein	2
252	tr∣A0A067NDZ6∣A0A067NDZ6_PLEOS	Proteasome subunit beta	1
253	tr∣Q9UVM4∣Q9UVM4_PLESA	HSP100	1
254	tr∣A0A6C0N3I6∣A0A6C0N3I6_PLEOS	Heat shock protein 100	1
255	tr∣A0A067P4K7∣A0A067P4K7_PLEOS	Clp R domain-containing protein	1
256	tr∣A0A067P4F3∣A0A067P4F3_PLEOS	Aspartyl-tRNA synthetase	1
257	tr∣A0A067NK65∣A0A067NK65_PLEOS	RRM domain-containing protein	1
258	tr∣A0A067NF75∣A0A067NF75_PLEOS	Redoxin domain-containing protein	2
259	tr∣A0A067NC76∣A0A067NC76_PLEOS	Obg-like ATPase 1	1
260	tr∣A0A067N6Z6∣A0A067N6Z6_PLEOS	DNA-directed RNA polymerase	1
261	tr∣A0A067PA61∣A0A067PA61_PLEOS	MFS domain-containing protein OS=Pleurotus ostreatus PC15 OX=1137138 GN=PLEOSDRAFT_153566 PE=4 SV=1	1
262	tr∣A0A067P946∣A0A067P946_PLEOS	ATP synthase subunit gamma (Fragment)	1
263	tr∣A0A067P8F7∣A0A067P8F7_PLEOS	Plug_translocon domain-containing protein	1
264	tr∣A0A067P286∣A0A067P286_PLEOS	Succinate--CoA ligase [ADP-forming] subunit beta, mitochondrial	1
265	tr∣A0A067P258∣A0A067P258_PLEOS	Cytochrome b-c1 complex subunit 7	1
266	tr∣A0A067P1V4∣A0A067P1V4_PLEOS	Citrate synthase	1
267	tr∣A0A067P193∣A0A067P193_PLEOS	Protein AF-9 homolog	1
268	tr∣A0A067P0D4∣A0A067P0D4_PLEOS	Proteasome subunit alpha type	2
269	tr∣A0A067NZG7∣A0A067NZG7_PLEOS	Glucosidase 2 subunit beta	1
270	tr∣A0A067NZF0∣A0A067NZF0_PLEOS	Dipeptidyl peptidase 3	1

271	tr|A0A067NYI3|A0A067NYI3_PLEOS	Adenylosuccinate synthetase	1
272	tr|A0A067NTN6|A0A067NTN6_PLEOS	GLTP domain-containing protein	1
273	tr|A0A067NTK7|A0A067NTK7_PLEOS	T-complex protein 1 subunit eta	1
274	tr|A0A067NS15|A0A067NS15_PLEOS	WD_REPEATS_REGION domain-containing protein	1
275	tr|A0A067NRV9|A0A067NRV9_PLEOS	Eukaryotic translation initiation factor 3 subunit B	1
276	tr|A0A067NRS7|A0A067NRS7_PLEOS	ARID domain-containing protein	1
277	tr|A0A067NRC3|A0A067NRC3_PLEOS	Peptide hydrolase	1
278	tr|A0A067NP99|A0A067NP99_PLEOS	Ubiquitin-like domain-containing protein	1
279	tr|A0A067NN37|A0A067NN37_PLEOS	Plant-expansin-like protein	1
280	tr|A0A067NMT1|A0A067NMT1_PLEOS	DJ-1_PfpI domain-containing protein	1
281	tr|A0A067NIC8|A0A067NIC8_PLEOS	AMP-binding domain-containing protein	1
282	tr|A0A067NGS6|A0A067NGS6_PLEOS	MFS domain-containing protein	1
283	tr|A0A067NG73|A0A067NG73_PLEOS	5-aminoimidazole-4-carboxamide ribonucleotide formyltransferase	1
284	tr|A0A067ND80|A0A067ND80_PLEOS	PSDC domain-containing protein	1
285	tr|A0A067ND43|A0A067ND43_PLEOS	Carbonic anhydrase	1
286	tr|A0A067N3J6|A0A067N3J6_PLEOS	Eukaryotic translation initiation factor 3 subunit A	1
287	tr|A0A067N2I3|A0A067N2I3_PLEOS	Phosphatase tensin-type domain-containing protein	1
288	tr|A0A067P591|A0A067P591_PLEOS	Histidine--tRNA ligase (Fragment)	1
289	tr|A0A067N3J5|A0A067N3J5_PLEOS	Serine hydroxymethyltransferase	1
290	tr|A0A067NX03|A0A067NX03_PLEOS	Ribosomal_L28e domain-containing protein	2
291	tr|A0A067NZQ8|A0A067NZQ8_PLEOS	Alanine--tRNA ligase	1
292	tr|A0A067NJ05|A0A067NJ05_PLEOS	Cytochrome c1, component of the mitochondrial respiratory chain	1
293	tr|A0A067P016|A0A067P016_PLEOS	Complex I-B22	1
294	tr|A0A067NKW8|A0A067NKW8_PLEOS	Fructose-bisphosphate aldolase	4
295	tr|A0A067NI38|A0A067NI38_PLEOS	Triosephosphate isomerase	1
296	tr|A0A067NI97|A0A067NI97_PLEOS	HMG box domain-containing protein (Fragment)	1
297	tr|A0A067NS12|A0A067NS12_PLEOS	HABP4_PAI-RBP1 domain-containing protein	1
298	tr|A0A067NG89|A0A067NG89_PLEOS	Extracellular metalloproteinase	1
299	tr|A0A067P1D3|A0A067P1D3_PLEOS	Septin-type G domain-containing protein	3
300	tr|A0A067NVK6|A0A067NVK6_PLEOS	Ferrochelatase	1
301	tr|A0A482GMN1|A0A482GMN1_PLEOS	Peroxidase	1
302	tr|A0A067NK95|A0A067NK95_PLEOS	Peroxidase	1
303	tr|A0A067NW47|A0A067NW47_PLEOS	S5 DRBM domain-containing protein	3
304	tr|A0A067NYB2|A0A067NYB2_PLEOS	Proteasome subunit alpha type	2
305	tr|A0A067NSA2|A0A067NSA2_PLEOS	Ribosomal_L7Ae domain-containing protein	1

306	tr|A0A067NWW1|A0A067NWW1_PLEOS	Catalase	2
307	tr|A0A482GQN6|A0A482GQN6_PLEOS	Catalase	2
308	tr|A0A2H4UZK6|A0A2H4UZK6_PLEOS	Catalase (Fragment)	2
309	tr|A0A067NGV7|A0A067NGV7_PLEOS	Prolyl-tRNA synthetase	1
310	tr|A0A067N7N1|A0A067N7N1_PLEOS	TOG domain-containing protein	1
311	tr|A0A067NL12|A0A067NL12_PLEOS	Carbohydrate esterase family 4 protein	1
312	tr|A0A067NJF8|A0A067NJF8_PLEOS	Carbohydrate esterase family 4 protein	1
313	tr|A0A067NGD7|A0A067NGD7_PLEOS	Carbohydrate esterase family 4 protein	1
314	tr|A0A067NPC3|A0A067NPC3_PLEOS	Beta-hexosaminidase	1
315	tr|A0A067NC11|A0A067NC11_PLEOS	Ribosomal_L23eN domain-containing protein (Fragment)	2
316	RRRRRtr|A0A067NNI2|A0A067NNI2_PLEOS	REVERSED Thymidylate synthase	1
317	tr|A0A067NME3|A0A067NME3_PLEOS	Serine/threonine-protein phosphatase	1
318	tr|A0A067NQC8|A0A067NQC8_PLEOS	Glycerol-3-phosphate dehydrogenase	1
319	tr|A0A067NL68|A0A067NL68_PLEOS	ATP synthase subunit delta, mitochondrial	1
320	tr|A0A067NHX4|A0A067NHX4_PLEOS	Malate synthase	1
321	tr|A0A067P416|A0A067P416_PLEOS	Adenylate kinase	1
322	tr|A0A067NU43|A0A067NU43_PLEOS	Adenosine kinase	2
323	tr|A0A067P386|A0A067P386_PLEOS	Acetylglucosamine phosphomutase	1
324	tr|A0A067NTP4|A0A067NTP4_PLEOS	40S ribosomal protein S26 (Fragment)	1
325	tr|A0A067PBS7|A0A067PBS7_PLEOS	ADH_zinc_N domain-containing protein	1
326	tr|A0A067P128|A0A067P128_PLEOS	PKS_ER domain-containing protein	1
327	RRRRRtr|A0A067NV39|A0A067NV39_PLEOS	REVERSED NUC153 domain-containing protein	1
328	tr|A0A067NV48|A0A067NV48_PLEOS	PKS_ER domain-containing protein	1
329	tr|A0A067PDH6|A0A067PDH6_PLEOS	GED domain-containing protein	1
330	tr|A0A067NVT9|A0A067NVT9_PLEOS	RRM domain-containing protein	1
331	tr|A0A067NY79|A0A067NY79_PLEOS	PCI domain-containing protein	0
332	tr|A0A067P2I9|A0A067P2I9_PLEOS	Small nuclear ribonucleoprotein Sm D2	1
333	RRRRRtr|A0A067NX83|A0A067NX83_PLEOS	REVERSED HET domain-containing protein	0
334	tr|A0A067NRA4|A0A067NRA4_PLEOS	Saccharopine dehydrogenase [NAD(+), L-lysine-forming]	1
335	tr|A0A067NMU1|A0A067NMU1_PLEOS	Pyruvate dehydrogenase E1 component subunit beta	0
336	tr|A0A067NSY7|A0A067NSY7_PLEOS	Aspzincin_M35 domain-containing protein	0
337	sp|P81055|PLMP_PLEOS	Peptidyl-Lys metalloendopeptidase	0
338	tr|A0A067PCL4|A0A067PCL4_PLEOS	Glutamate decarboxylase (Fragment)	0
339	tr|A0A067N3K5|A0A067N3K5_PLEOS	NmrA domain-containing protein	1
340	tr|A0A067NP91|A0A067NP91_PLEOS	Ribosomal protein	0
341	tr|A0A067NN60|A0A067NN60_PLEOS	26S proteasome regulatory subunit RPN11	0
342	RRRRRtr|A0A067NGR3|A0A067NGR3_PLEOS	REVERSED DNA polymerase epsilon catalytic subunit	0

343	tr|A0A067N2J4|A0A067N2J4_PLEOS	26S proteasome regulatory subunit RPN1	0
344	tr|A0A067N9H1|A0A067N9H1_PLEOS	PCI domain-containing protein	0
345	RRRRRtr|A0A067P1S2|A0A067P1S2_PLEOS	REVERSED Ras modification protein ERF4	0
346	tr|A0A067NT34|A0A067NT34_PLEOS	Non-specific serine/threonine protein kinase	0
347	tr|A0A067NTR0|A0A067NTR0_PLEOS	DSHCT domain-containing protein	0
348	tr|A0A067NUZ3|A0A067NUZ3_PLEOS	Phosphoenolpyruvate carboxykinase (ATP) (Fragment)	0
349	tr|A0A067NV79|A0A067NV79_PLEOS	Proteasome subunit alpha type	0
350	tr|A0A067NPK7|A0A067NPK7_PLEOS	Isocitrate lyase	2
351	tr|A0A067NBL2|A0A067NBL2_PLEOS	60S ribosomal protein L41	0
352	tr|A0A067P2R6|A0A067P2R6_PLEOS	Aminopeptidase	0
353	tr|A0A067NKU9|A0A067NKU9_PLEOS	U6 snRNA-associated Sm-like protein LSm2	0
354	tr|A0A067NEQ4|A0A067NEQ4_PLEOS	MPN domain-containing protein	0
355	tr|A0A067NFV0|A0A067NFV0_PLEOS	HDAC_interact domain-containing protein	0
356	tr|A0A067P075|A0A067P075_PLEOS	NADPH:adrenodoxin oxidoreductase, mitochondrial	1
357	tr|A0A067NIR7|A0A067NIR7_PLEOS	Endoplasmic reticulum transmembrane protein	0
358	tr|A0A067NEB8|A0A067NEB8_PLEOS	Eukaryotic translation initiation factor 3 subunit G	0
359	tr|A0A067NMY5|A0A067NMY5_PLEOS	Translationally-controlled tumor protein homolog	0
360	tr|A0A067NEH1|A0A067NEH1_PLEOS	ATP synthase subunit 4, mitochondrial	0
361	tr|A0A067NDI3|A0A067NDI3_PLEOS	RRM domain-containing protein	0
362	tr|A0A067N771|A0A067N771_PLEOS	NMO domain-containing protein (Fragment)	0
363	tr|A0A067NSN4|A0A067NSN4_PLEOS	Methylmalonate-semialdehyde dehydrogenase (CoA acylating)	0
364	tr|A0A067P2R8|A0A067P2R8_PLEOS	DH domain-containing protein	0
365	tr|A0A067PAB4|A0A067PAB4_PLEOS	HMA domain-containing protein	0
366	tr|A0A067NFS2|A0A067NFS2_PLEOS	Ribosomal protein L37	0
367	tr|A0A067NSE6|A0A067NSE6_PLEOS	Ribosomal protein L19	0
368	RRRRRtr|A0A067NBU5|A0A067NBU5_PLEOS	REVERSED WD_REPEATS_REGION domain-containing protein	0
369	tr|A0A067N9Q5|A0A067N9Q5_PLEOS	Cytochrome c oxidase subunit OS=Pleurotus ostreatus PC15 OX=1137138 GN=COX6B PE=3 SV=1	0
370	tr|A0A067NYJ9|A0A067NYJ9_PLEOS	t-SNARE coiled-coil homology domain-containing protein	0
371	RRRRRtr|A0A067NE50|A0A067NE50_PLEOS	REVERSED Autophagy-related protein 27	0
372	RRRRRtr|A0A6B9EPK4|A0A6B9EPK4_PLEOS	REVERSED Zinc cluster transcription factor 23 (Fragment)	0

373	RRRRRtr|A0A067NJ43|A0A067NJ43_PLEOS	REVERSED Fungal_trans domain-containing protein (Fragment)	0
374	tr|A0A067PBI1|A0A067PBI1_PLEOS	EH domain-containing protein	0
375	tr|A0A067NWK2|A0A067NWK2_PLEOS	NTP_transferase domain-containing protein	0
376	tr|A0A067NT85|A0A067NT85_PLEOS	SMP-LTD domain-containing protein	0
377	RRRRRtr|A0A067PCN9|A0A067PCN9_PLEOS	REVERSED DHR10 domain-containing protein	0
378	tr|A0A067NP59|A0A067NP59_PLEOS	Multifunctional fusion protein	0
379	tr|A0A067NY35|A0A067NY35_PLEOS	M20_dimer domain-containing protein	0
380	tr|A0A067PCH4|A0A067PCH4_PLEOS	Actin cytoskeleton-regulatory complex protein PAN1	0
381	tr|A0A067PB77|A0A067PB77_PLEOS	ABC transporter domain-containing protein	0
382	tr|A0A067NLH9|A0A067NLH9_PLEOS	Calcineurin regulatory subunit	0
383	tr|A0A067N9H4|A0A067N9H4_PLEOS	Methionyl-tRNA synthetase	0
384	RRRRRtr|A0A067PAL9|A0A067PAL9_PLEOS	REVERSED AB hydrolase-1 domain-containing protein	0
385	RRRRRtr|A0A067NLN9|A0A067NLN9_PLEOS	REVERSED Actin cytoskeleton-regulatory complex protein SLA1	0
386	tr|A0A067NY19|A0A067NY19_PLEOS	DHHA2 domain-containing protein	0
387	tr|A0A067NQ58|A0A067NQ58_PLEOS	Proteasome subunit beta	0
388	tr|A0A067NAN9|A0A067NAN9_PLEOS	DUF1996 domain-containing protein	0
389	RRRRRtr|A0A067P3E6|A0A067P3E6_PLEOS	REVERSED Pep3_Vps18 domain-containing protein	0
390	RRRRRtr|A0A067NJ09|A0A067NJ09_PLEOS	REVERSED Git3 domain-containing protein	0
391	RRRRRtr|A0A067ND53|A0A067ND53_PLEOS	REVERSED JmjC domain-containing protein	0
392	RRRRRtr|A0A067N2H6|A0A067N2H6_PLEOS	REVERSED JmjC domain-containing protein	0
393	tr|A0A067NDL9|A0A067NDL9_PLEOS	Eukaryotic translation initiation factor 3 subunit I	0
394	tr|A0A067P2I0|A0A067P2I0_PLEOS	GAE domain-containing protein	0
395	tr|A0A067NFG1|A0A067NFG1_PLEOS	AP-1 complex subunit gamma	0
396	tr|A0A067P131|A0A067P131_PLEOS	Phosphotransferase	0
397	tr|A0A067NXH2|A0A067NXH2_PLEOS	Aldo_ket_red domain-containing protein	0
398	tr|A0A067N2S3|A0A067N2S3_PLEOS	HpcH_HpaI domain-containing protein (Fragment)	0
399	RRRRRtr|A0A067PCR2|A0A067PCR2_PLEOS	REVERSED Cnn_1N domain-containing protein	0
400	tr|A0A067P091|A0A067P091_PLEOS	CCT-alpha	0
401	tr|A0A067NUN3|A0A067NUN3_PLEOS	Plasma membrane ATPase	0
402	RRRRRtr|A0A2K9YPK4|A0A2K9YPK4_9AGAR	REVERSED Homing endonuclease	0

Table S3: Protein identification of *Agricus bisporus by Uniprot database*

**Number**	**Accession number**	**Protein name**	**Peptide (95 %)**
1	tr|A0A369JNN0|A0A369JNN0_HYPMA	Heat shock protein HSS1	24
2	tr|A0A369J7D3|A0A369J7D3_HYPMA	Actin-1	20
3	tr|K5Y6N0|K5Y6N0_AGABU	Elongation factor 1-alpha	21
4	tr|A0A369JCW3|A0A369JCW3_HYPMA	14-3-3 protein	17
5	tr|K5Y3Q8|K5Y3Q8_AGABU	ATP synthase subunit beta	15
6	tr|A0A369JIS9|A0A369JIS9_HYPMA	ATP synthase subunit beta	17
7	tr|A0A369JS72|A0A369JS72_HYPMA	Heat shock protein 90	13
8	tr|A0A369JUF6|A0A369JUF6_HYPMA	Heat shock protein sks2	10
9	tr|A0A369JL66|A0A369JL66_HYPMA	Tubulin beta chain	12
10	tr|A0A369KER8|A0A369KER8_HYPMA	Elongation factor 2	8
11	tr|A0A369K5X4|A0A369K5X4_HYPMA	Inorganic diphosphatase	5
12	tr|A0A369JBW1|A0A369JBW1_HYPMA	Endoplasmic reticulum chaperone BiP	9
13	tr|A0A369J8Y1|A0A369J8Y1_HYPMA	Cell division cycle protein 48	6
14	tr|K5WW32|K5WW32_AGABU	Catalase	8
15	tr|K5Y7K9|K5Y7K9_AGABU	Phosphopyruvate hydratase	10
16	tr|A0A369JYX9|A0A369JYX9_HYPMA	UDP-glucose pyrophosphorylase	6
17	tr|A0A369K708|A0A369K708_HYPMA	Pyruvate kinase	4
18	tr|K5XAS8|K5XAS8_AGABU	5-methyltetrahydropteroyltriglutamate--homocysteine S-methyltransferase =1	8
19	tr|K5XEW1|K5XEW1_AGABU	Ubiquitin-like domain-containing protein	7
20	tr|K5WX04|K5WX04_AGABU	Ubiquitin (Fragment)	7
21	tr|A0A369JSM1|A0A369JSM1_HYPMA	Septin spn4	4
22	tr|A0A369JWP4|A0A369JWP4_HYPMA	Proteasome subunit alpha type	4
23	tr|K5WY21|K5WY21_AGABU	Proteasome subunit alpha type	4
24	tr|A0A369JNM7|A0A369JNM7_HYPMA	GTP-binding nuclear protein	4
25	tr|K5Y3K4|K5Y3K4_AGABU	GTP-binding nuclear protein	3
26	tr|A0A369JF09|A0A369JF09_HYPMA	40S ribosomal protein S7	4
27	tr|A0A369JGA3|A0A369JGA3_HYPMA	Malate dehydrogenase	7
28	tr|K5Y2Q9|K5Y2Q9_AGABU	Malate dehydrogenase	6
29	tr|K5X1I1|K5X1I1_AGABU	ATP synthase subunit alpha	4
30	tr|K5WPY6|K5WPY6_AGABU	Histone H2B	4
31	tr|K5W749|K5W749_AGABU	Histone H2B	4
32	tr|A0A369K5F8|A0A369K5F8_HYPMA	Histone H2B	4
33	tr|A0A369K055|A0A369K055_HYPMA	Histone H2B	4
34	tr|A0A369JHR7|A0A369JHR7_HYPMA	Histone H2B	4
35	tr|A0A369JGV4|A0A369JGV4_HYPMA	Histone H2B	4
36	sp|P78567|H2B_AGABI	Histone H2B	4
37	tr|A0A369JL95|A0A369JL95_HYPMA	ATP-dependent RNA helicase eIF4A	4
38	tr|Q6KFX3|Q6KFX3_FLAVE	Glyceraldehyde-3-phosphate dehydrogenase	6
39	tr|G8FUB1|G8FUB1_HYPMA	Glyceraldehyde-3-phosphate dehydrogenase	3

40	tr|Q708W0|Q708W0_ARMTA	Glyceraldehyde-3-phosphate dehydrogenase	3
41	tr|K5Y3E9|K5Y3E9_AGABU	E3 ubiquitin ligase complex SCF subunit	4
42	tr|A0A369JSC8|A0A369JSC8_HYPMA	E3 ubiquitin ligase complex SCF subunit	4
43	tr|A0A369K4J4|A0A369K4J4_HYPMA	Myosin regulatory light chain cdc4	3
44	tr|A0A369K5N3|A0A369K5N3_HYPMA	Clp R domain-containing protein	3
45	tr|A0A369K1R4|A0A369K1R4_HYPMA	Cell division control protein 3	4
46	tr|K5X4D4|K5X4D4_AGABU	Transket_pyr domain-containing protein	5
47	tr|A0A369KF41|A0A369KF41_HYPMA	Rab GDP dissociation inhibitor	3
48	tr|K5XJ91|K5XJ91_AGABU	Rab GDP dissociation inhibitor	2
49	tr|A0A369J245|A0A369J245_HYPMA	Adenosylhomocysteinase	2
50	tr|K5WYA1|K5WYA1_AGABU	Adenosylhomocysteinase	2
51	tr|A0A369K4Q2|A0A369K4Q2_HYPMA	40S ribosomal protein S16-B	3
52	tr|A0A369JUK2|A0A369JUK2_HYPMA	Formate dehydrogenase	2
53	tr|K5XZ43|K5XZ43_AGABU	Formate dehydrogenase	2
54	tr|A0A369JFC8|A0A369JFC8_HYPMA	Elongation factor 2	7
55	tr|A0A369K981|A0A369K981_HYPMA	Septin spn3	2
56	tr|A0A369J209|A0A369J209_HYPMA	Fructose-bisphosphate aldolase	3
57	tr|A0A369K706|A0A369K706_HYPMA	Elongation factor 1-alpha	19
58	tr|A0A369JWX6|A0A369JWX6_HYPMA	Catalase	10
59	tr|K5X1D1|K5X1D1_AGABU	Peptidyl-prolyl cis-trans isomerase	7
60	tr|A0A369K703|A0A369K703_HYPMA	Peptidyl-prolyl cis-trans isomerase	4
61	tr|K5XAT3|K5XAT3_AGABU	Thioredoxin domain-containing protein	3
62	tr|A0A369JSV9|A0A369JSV9_HYPMA	Peroxiredoxin-6	3
63	tr|A0A369K866|A0A369K866_HYPMA	6-phosphogluconate dehydrogenase, decarboxylating	4
64	tr|A0A369JEX3|A0A369JEX3_HYPMA	3-oxoacyl-[acyl-carrier-protein] reductase FabG	2
65	tr|A0A369JK29|A0A369JK29_HYPMA	Multifunctional fusion protein	3
66	tr|K5XPB2|K5XPB2_AGABU	Malate dehydrogenase	8
67	tr|A0A369K0W7|A0A369K0W7_HYPMA	Putative voltage-gated potassium channel subunit beta	2
68	tr|A0A369JR16|A0A369JR16_HYPMA	40S ribosomal protein S0	3
69	tr|K5XBE5|K5XBE5_AGABU	40S ribosomal protein S0	3
70	tr|A0A369JZT9|A0A369JZT9_HYPMA	Aconitate hydratase, mitochondrial	4
71	tr|K5X277|K5X277_AGABU	Histone H4	2
72	tr|K5X226|K5X226_AGABU	Histone H4 (Fragment)	2
73	tr|A0A369J1D5|A0A369J1D5_HYPMA	Histone H4 (Fragment)	2
74	tr|A0A151VES4|A0A151VES4_HYPMA	Histone H4	2
75	sp|P62793|H4_AGABI	Histone H4	2
76	tr|K5Y2C2|K5Y2C2_AGABU	Histone H4	2
77	tr|A0A369KBU1|A0A369KBU1_HYPMA	Nascent polypeptide-associated complex subunit beta	3

78	tr|K5XPW2|K5XPW2_AGABU	Nascent polypeptide-associated complex subunit beta	2
79	tr|K5XVV4|K5XVV4_AGABU	AAA domain-containing protein	2
80	tr|A0A369J7L7|A0A369J7L7_HYPMA	26S protease regulatory subunit 6A	2
81	tr|K5W7Q2|K5W7Q2_AGABU	Phosphoglycerate kinase	2
82	tr|A0A369JQY3|A0A369JQY3_HYPMA	Phosphoglycerate kinase	1
83	sp|O94123|PGK_AGABI	Phosphoglycerate kinase	1
84	tr|A0A369JYY6|A0A369JYY6_HYPMA	Triosephosphate isomerase	2
85	tr|K5WQY5|K5WQY5_AGABU	Triosephosphate isomerase	1
86	tr|K5WX38|K5WX38_AGABU	Tubulin alpha chain	4
87	tr|A0A369JN52|A0A369JN52_HYPMA	Tubulin alpha chain	4
88	tr|A0A369JKN3|A0A369JKN3_HYPMA	Tubulin alpha chain	1
89	tr|A0A369JWT4|A0A369JWT4_HYPMA	Trehalose phosphorylase	3
90	tr|K5XI74|K5XI74_AGABU	Glycos_transf_1 domain-containing protein SV=1	2
91	tr|A0A0G2SWS6|A0A0G2SWS6_FLAVE	Trehalose synthase/trehalose phosphorylase (Fragment)	2
92	tr|A0A369J7L9|A0A369J7L9_HYPMA	40S ribosomal protein S19-A	2
93	tr|K5XWZ7|K5XWZ7_AGABU	40S ribosomal protein S12	2
94	tr|A0A369JG77|A0A369JG77_HYPMA	40S ribosomal protein S12	1
95	tr|A0A369JJG1|A0A369JJG1_HYPMA	Protein GVP36	2
96	tr|A0A369JT17|A0A369JT17_HYPMA	Alanine--tRNA ligase	3
97	tr|A0A369KGF1|A0A369KGF1_HYPMA	Histone H2A	2
98	tr|K5X2E9|K5X2E9_AGABU	Histone H2A	2
99	tr|A0A369KAE9|A0A369KAE9_HYPMA	Histone H2A	2
100	tr|A0A369JMC0|A0A369JMC0_HYPMA	Histone H2A	2
101	tr|A0A369KAM2|A0A369KAM2_HYPMA	Histone H2A	2
102	tr|K5XRC6|K5XRC6_AGABU	Histone H2A	2
103	tr|K5XPF5|K5XPF5_AGABU	Histone H2A	2
104	tr|A0A369JDR4|A0A369JDR4_HYPMA	Histone H2A.Z	2
105	sp|Q9HGX4|H2A_AGABI	Histone H2A	2
106	tr|K5WXK2|K5WXK2_AGABU	Ribosomal_S13_N domain-containing protein SV=1	2
107	tr|A0A369J4J8|A0A369J4J8_HYPMA	40S ribosomal protein S13	2
108	sp|P78571|RS13_AGABI	40S ribosomal protein S13	2
109	tr|K5X3G1|K5X3G1_AGABU	Aldedh domain-containing protein	5
110	sp|O74187|ALDH_AGABI	Aldehyde dehydrogenase	5
111	tr|A0A369JV15|A0A369JV15_HYPMA	40S ribosomal protein S14	2
112	tr|A0A369IZW7|A0A369IZW7_HYPMA	Mitochondrial-processing peptidase subunit beta	2
113	tr|D2JY95|D2JY95_FLAVE	40S ribosomal protein S5-1	2
114	tr|K5Y051|K5Y051_AGABU	Ribosomal_S7 domain-containing protein	2
115	tr|A0A369JXV9|A0A369JXV9_HYPMA	40S ribosomal protein S5	2
116	tr|A0A369JDR1|A0A369JDR1_HYPMA	40S ribosomal protein S2	3

117	tr|K5WZF0|K5WZF0_AGABU	S5 DRBM domain-containing protein	2
118	tr|A0A369JND0|A0A369JND0_HYPMA	Glucose-6-phosphate 1-dehydrogenase	1
119	tr|A0A369JCR5|A0A369JCR5_HYPMA	ATP-citrate (pro-S-)-lyase	1
120	tr|K5X1D8|K5X1D8_AGABU	ATP-citrate synthase	1
121	tr|A0A369JLI6|A0A369JLI6_HYPMA	Homocysteine synthase	3
122	tr|A0A369JJP4|A0A369JJP4_HYPMA	Homocysteine synthase	3
123	tr|A0A369KA85|A0A369KA85_HYPMA	D-arabinitol dehydrogenase 1	1
124	tr|A0A369JN44|A0A369JN44_HYPMA	Glutamine synthetase	2
125	tr|A0A369JPZ9|A0A369JPZ9_HYPMA	40S ribosomal protein S21	2
126	tr|K5VLR9|K5VLR9_AGABU	Plasma membrane ATPase	2
127	tr|A0A369JVJ7|A0A369JVJ7_HYPMA	Vacuolar aspartic protease	2
128	tr|D2JY88|D2JY88_FLAVE	60S ribosomal protein L18	1
129	tr|K5XFY0|K5XFY0_AGABU	Ribosomal_L18e/L15P domain-containing protein	1
130	tr|A0A369JP11|A0A369JP11_HYPMA	60S ribosomal protein L18-B	1
131	tr|A0A369JWN9|A0A369JWN9_HYPMA	60S ribosomal protein L23-B	1
132	tr|A0A369KA18|A0A369KA18_HYPMA	Oligouridylate-binding protein 1	0
133	tr|A0A369JTK1|A0A369JTK1_HYPMA	Isocitrate dehydrogenase [NAD] subunit, mitochondrial	2
134	tr|A0A369JSL1|A0A369JSL1_HYPMA	Tropomyosin-2 OS=Hypsizygus marmoreus	1
135	tr|A0A369JPH0|A0A369JPH0_HYPMA	S-adenosylmethionine synthase	2
136	tr|K5XHA2|K5XHA2_AGABU	Polyadenylate-binding protein	1
137	tr|A0A369JKM5|A0A369JKM5_HYPMA	Polyadenylate-binding protein	1
138	tr|A0A369JPI3|A0A369JPI3_HYPMA	Ubiquitin-activating enzyme E1 1	1
139	tr|K5XK69|K5XK69_AGABU	Eukaryotic translation initiation factor 5A	2
140	tr|D2JY86|D2JY86_FLAVE	Eukaryotic translation initiation factor 5A	2
141	tr|A0A369JHR9|A0A369JHR9_HYPMA	Glucose-6-phosphate isomerase	1
142	tr|A0A369JGP9|A0A369JGP9_HYPMA	Proteasome subunit alpha type	2
143	tr|A0A369K2Q9|A0A369K2Q9_HYPMA	Programmed cell death protein 6	1
144	tr|A0A369KC73|A0A369KC73_HYPMA	60S ribosomal protein L4-B	2
145	tr|A0A369JTI5|A0A369JTI5_HYPMA	Putative 26S protease subunit rpt4	2
146	tr|A0A369JSJ9|A0A369JSJ9_HYPMA	Aldehyde dehydrogenase	2
147	tr|A0A369J1T6|A0A369J1T6_HYPMA	60S ribosomal protein L11	2
148	tr|A0A369JL46|A0A369JL46_HYPMA	1,4-alpha-glucan-branching enzyme	1
149	tr|K5XX41|K5XX41_AGABU	1,4-alpha-glucan-branching enzyme	1
150	tr|A0A369JXQ1|A0A369JXQ1_HYPMA	Alpha-NAC	2
151	tr|K5X5B6|K5X5B6_AGABU	Alpha-NAC	1
152	tr|K5Y6Y1|K5Y6Y1_AGABU	Elongation factor 2	9
153	tr|A0A369JS64|A0A369JS64_HYPMA	Malate dehydrogenase	4
154	tr|A0A369K1W0|A0A369K1W0_HYPMA	D-galacturonate reductase	1
155	tr|A0A369JYT6|A0A369JYT6_HYPMA	Glycerol 2-dehydrogenase (NADP(+))	1
156	tr|A0A369JWH8|A0A369JWH8_HYPMA	Aldo_ket_red domain-containing protein	1
157	tr|K5WR11|K5WR11_AGABU	Transaldolase	2

158	tr|A0A369J3A6|A0A369J3A6_HYPMA	Transaldolase	2
159	tr|A0A369K4M7|A0A369K4M7_HYPMA	Acetylglucosamine phosphomutase	1
160	tr|A0A369JI09|A0A369JI09_HYPMA	Linoleate 10R-lipoxygenase	1
161	tr|A0A369KEH5|A0A369KEH5_HYPMA	Aldehyde dehydrogenase	2
162	tr|K5Y799|K5Y799_AGABU	Proteasome subunit beta	2
163	tr|A0A369K4H1|A0A369K4H1_HYPMA	Proteasome subunit beta	2
164	tr|A0A369J9Y1|A0A369J9Y1_HYPMA	Superoxide dismutase	6
165	tr|P78566|P78566_AGABI	Heat shock protein 70 (Fragment)	3
166	tr|K5XK18|K5XK18_AGABU	6-phosphogluconate dehydrogenase, decarboxylating	3
167	tr|G8A525|G8A525_FLAVE	Glutamine synthetase OS=Flammulina velutipes	1
168	tr|A0A369KDK0|A0A369KDK0_HYPMA	D-fructose-6-phosphate amidotransferase	1
169	tr|K5XLG7|K5XLG7_AGABU	T-complex protein 1 subunit gamma	1
170	tr|K5WVB2|K5WVB2_AGABU	Mitochondrial intermediate peptidase	1
171	tr|K5VSL0|K5VSL0_AGABU	UBC core domain-containing protein	1
172	tr|A0A369JZM2|A0A369JZM2_HYPMA	Ubiquitin-conjugating enzyme E2	1
173	tr|A0A369K201|A0A369K201_HYPMA	60S acidic ribosomal protein P0	1
174	tr|K5X6P0|K5X6P0_AGABU	60S acidic ribosomal protein P0	1
175	tr|A0A369JZI9|A0A369JZI9_HYPMA	60S ribosomal protein L14-A	2
176	tr|A0A369JT84|A0A369JT84_HYPMA	Leucine-rich repeat-containing protein 10B	1
177	tr|A0A369JPK0|A0A369JPK0_HYPMA	GTP-binding protein ypt1	1
178	tr|A0A369JH42|A0A369JH42_HYPMA	Aspartate aminotransferase	1
179	tr|A0A369J1P5|A0A369J1P5_HYPMA	Aldehyde dehydrogenase	1
180	tr|K5Y386|K5Y386_AGABU	Septin-type G domain-containing protein	1
181	tr|A0A369JP37|A0A369JP37_HYPMA	Septin spn2	1
182	tr|K5XXS7|K5XXS7_AGABU	Peptidase A1 domain-containing protein	1
183	tr|A0A369K020|A0A369K020_HYPMA	T-complex protein 1 subunit zeta	1
184	tr|A0A369K2X8|A0A369K2X8_HYPMA	Vacuolar protein 8	1
185	tr|K5X072|K5X072_AGABU	40S ribosomal protein S26 (Fragment)	1
186	tr|A0A369KEW7|A0A369KEW7_HYPMA	40S ribosomal protein S26	1
187	tr|K5WYQ3|K5WYQ3_AGABU	GLTP domain-containing protein	1
188	tr|A0A369K2J4|A0A369K2J4_HYPMA	Glycolipid transfer protein	1
189	tr|K5WVJ0|K5WVJ0_AGABU	Ribosomal_L16 domain-containing protein (Fragment) PE=4 SV=1	1
190	tr|K5WV26|K5WV26_AGABU	cobW domain-containing protein	1
191	tr|K5W4N2|K5W4N2_AGABU	ANK_REP_REGION domain-containing proteinSV=1	1
192	tr|K5W281|K5W281_AGABU	UV excision repair protein RAD23	1
193	tr|A0A369JT05|A0A369JT05_HYPMA	60S ribosomal protein L12	1
194	tr|A0A7G4WM60|A0A7G4WM60_9AGAR	Diphosphomevalonate decarboxylase	1
195	tr|A0A369KC08|A0A369KC08_HYPMA	Citrate synthase	2

196	tr|A0A369K434|A0A369K434_HYPMA	Ketol-acid reductoisomerase, mitochondrial	1
197	tr|A0A369JY18|A0A369JY18_HYPMA	Mitochondrial phosphate carrier protein 3, mitochondrial	1
198	tr|A0A369JXX7|A0A369JXX7_HYPMA	60S ribosomal protein L24	1
199	tr|A0A369JS89|A0A369JS89_HYPMA	Disulfide-bond oxidoreductase YfcG	1
200	tr|A0A369JBS6|A0A369JBS6_HYPMA	Disulfide-bond oxidoreductase YfcG	1
201	tr|A0A369JNV0|A0A369JNV0_HYPMA	NADP-dependent 3-hydroxy acid dehydrogenase	1
202	tr|A0A369JNB3|A0A369JNB3_HYPMA	Proteasome subunit alpha type	1
203	tr|A0A369JH95|A0A369JH95_HYPMA	Testicular acid phosphatase	1
204	tr|A0A369JFQ9|A0A369JFQ9_HYPMA	Actin-related protein 2/3 complex subunit 5	1
205	tr|A0A369JBG2|A0A369JBG2_HYPMA	Ran GTPase-activating protein 1	1
206	tr|A0A369J5B3|A0A369J5B3_HYPMA	ATP synthase subunit gamma	1
207	RRRRRtr|K5WRX4|K5WRX4_AGABU	REVERSED MBOAT_2 domain-containing protein	1
208	RRRRRtr|A0A369K569|A0A369K569_HYPMA	REVERSED DNA replication ATP-dependent helicase/nuclease	1
209	tr|K5XLD9|K5XLD9_AGABU	CYTOSOL_AP domain-containing protein	1
210	tr|K5WRT2|K5WRT2_AGABU	Hva1_TUDOR domain-containing protein	1
211	tr|A0A369K8F1|A0A369K8F1_HYPMA	40S ribosomal protein S17-A	1
212	tr|K5XPY9|K5XPY9_AGABU	Alpha-1,4 glucan phosphorylase	1
213	tr|A0A369JUR4|A0A369JUR4_HYPMA	Protein disulfide-isomerase	1
214	tr|A0A369K6W1|A0A369K6W1_HYPMA	Calmodulin	3
215	sp|P84339|CALM_AGABI	Calmodulin	3
216	tr|R4HKU2|R4HKU2_FLAVE	Myosin regulatory light chain	1
217	tr|K5X865|K5X865_AGABU	Inorganic diphosphatase	5
218	tr|K5X8L1|K5X8L1_AGABU	Proteasome subunit alpha type	3
219	tr|A0A369KBQ2|A0A369KBQ2_HYPMA	Proteasome subunit alpha type	2
220	tr|A0A369JQM2|A0A369JQM2_HYPMA	60S ribosomal protein L6	1
221	tr|K5WAD7|K5WAD7_AGABU	KH type-2 domain-containing protein	1
222	tr|A0A369K4W2|A0A369K4W2_HYPMA	40S ribosomal protein S3	1
223	tr|A0A369J7S7|A0A369J7S7_HYPMA	60S ribosomal protein L31	1
224	tr|A0A369JFN7|A0A369JFN7_HYPMA	T-complex protein 1 subunit delta	1
225	tr|K5Y3R7|K5Y3R7_AGABU	T-complex protein 1 subunit delta	1
226	tr|K5WHZ4|K5WHZ4_AGABU	Inosine-5'-monophosphate dehydrogenase	1
227	tr|K5Y6C4|K5Y6C4_AGABU	Prohibitin	1
228	tr|A0A369K8R3|A0A369K8R3_HYPMA	Prohibitin	1
229	tr|K5X898|K5X898_AGABU	Vacuolar proton pump subunit B	1
230	tr|A0A369JC65|A0A369JC65_HYPMA	Vacuolar proton pump subunit B	1
231	tr|A0A369J9S5|A0A369J9S5_HYPMA	Protein CFT1	1
232	tr|K5XIF2|K5XIF2_AGABU	Cell division control protein 42 homolog	1

233	tr|A0A369JWM8|A0A369JWM8_HYPMA	Cell division control protein 42 homolog	1
234	tr|A0A6C0N024|A0A6C0N024_FLAVE	Small GTPase Rac1	1
235	tr|A0A369JER8|A0A369JER8_HYPMA	GTP-binding protein rhoA	1
236	tr|K5WXJ5|K5WXJ5_AGABU	Ribosomal_L23eN domain-containing protein	1
237	tr|K5XK56|K5XK56_AGABU	Small nuclear ribonucleoprotein Sm D2	1
238	tr|A0A369JPJ9|A0A369JPJ9_HYPMA	60S ribosomal protein L36-A	1
239	tr|K5VUM6|K5VUM6_AGABU	Cytochrome c oxidase subunit	1
240	tr|K5W2T2|K5W2T2_AGABU	UTP--glucose-1-phosphate uridylyltransferase	5
241	tr|A0A369JMB5|A0A369JMB5_HYPMA	Peptidylprolyl isomerase	2
242	tr|A0A369JL47|A0A369JL47_HYPMA	Cytochrome c oxidase subunit 6, mitochondrial	1
243	tr|A0A369K761|A0A369K761_HYPMA	UDP-N-acetylhexosamine pyrophosphorylase	1
244	tr|A0A369JU17|A0A369JU17_HYPMA	Proteasome subunit beta	2
245	tr|K5XXY6|K5XXY6_AGABU	Aminotran_1_2 domain-containing protein	1
246	tr|H9ZYN3|H9ZYN3_AGABI	1-aminocyclopropane-1-carboxylate synthase 1	1
247	tr|A0A369JR54|A0A369JR54_HYPMA	Putative aminotransferase C6B12.04c	1
248	tr|A0A369K2J0|A0A369K2J0_HYPMA	Zinc-type alcohol dehydrogenase-like protein C2E1P3.01	1
249	tr|K5XUL4|K5XUL4_AGABU	PKS_ER domain-containing protein 1	1
250	tr|A0A369K711|A0A369K711_HYPMA	Zinc-type alcohol dehydrogenase-like protein C2E1P3.01	1
251	tr|A0A369KCG7|A0A369KCG7_HYPMA	Guanine nucleotide-binding protein subunit beta	1
252	tr|A0A2R3STX6|A0A2R3STX6_FLAVE	Guanine nucleotide-binding protein beta subunit	1
253	tr|A0A0A7DLB0|A0A0A7DLB0_HYPMA	Guanine nucleotide binding protein beta subunit 2	1
254	tr|K5VWN2|K5VWN2_AGABU	Proteasome subunit beta	1
255	tr|A0A369JWG3|A0A369JWG3_HYPMA	Proteasome endopeptidase complex	1
256	tr|A0A369JKI2|A0A369JKI2_HYPMA	60S ribosomal protein L25	1
257	tr|A0A369JWJ9|A0A369JWJ9_HYPMA	Glutamate decarboxylase	1
258	tr|A0A369JMQ9|A0A369JMQ9_HYPMA	Heat shock protein 16	1
259	tr|A0A369JG01|A0A369JG01_HYPMA	Metal homeostasis factor ATX1	1
260	tr|K5XUD6|K5XUD6_AGABU	Ketol-acid reductoisomerase, mitochondrial SV=1	1
261	tr|A0A7G4WM57|A0A7G4WM57_9AGAR	Acetyl-CoA C-acetyltransferase	1
262	tr|K5XBA3|K5XBA3_AGABU	Aldedh domain-containing protein	1
263	tr|A0A369J755|A0A369J755_HYPMA	60S ribosomal protein L17	0
264	tr|A0A369JTH4|A0A369JTH4_HYPMA	Phosphoglucomutase	4
265	tr|K5VWK2|K5VWK2_AGABU	Serine/threonine-protein phosphatase	1

266	tr|A0A369JYB4|A0A369JYB4_HYPMA	Serine/threonine-protein phosphatase	1
267	tr|A0A369K2V1|A0A369K2V1_HYPMA	Putative NAD(P)H-dependent D-xylose reductase xyl1	1
268	tr|K5XNX4|K5XNX4_AGABU	Extracellular metalloproteinase	1
269	tr|A0A369JKG5|A0A369JKG5_HYPMA	Carbonyl reductase family member 4	1
270	tr|A0A7G6UGX1|A0A7G6UGX1_9AGAR	Glyceraldehyde-3-phosphate dehydrogenase	5
271	tr|U6BJE7|U6BJE7_9AGAR	Glyceraldehyde-3-phosphate dehydrogenase (phosphorylating) (Fragment)	2
272	tr|A0A369JXA0|A0A369JXA0_HYPMA	Calcium-transporting ATPase	1
273	tr|A0A369KAA9|A0A369KAA9_HYPMA	Putative oxidoreductase YfjR	1
274	tr|A0A369J5I4|A0A369J5I4_HYPMA	Midasin	0
275	RRRRRtr|A0A369KEL4|A0A369KEL4_HYPMA	REVERSED F-box domain-containing protein	1
276	tr|K5XBY3|K5XBY3_AGABU	WD_REPEATS_REGION domain-containing protein	0
277	tr|A0A223GEB2|A0A223GEB2_FLAVE	Cross-pathway control protein	0
278	tr|A0A369JUZ7|A0A369JUZ7_HYPMA	Guanine nucleotide-binding protein subunit beta-like protein	0
279	tr|A0A369JSI2|A0A369JSI2_HYPMA	Coatomer subunit beta	0
280	tr|A0A369JKV9|A0A369JKV9_HYPMA	40S ribosomal protein S1	0
281	tr|A0A369JY97|A0A369JY97_HYPMA	Septin spn3	0
282	tr|K5X3I7|K5X3I7_AGABU	Acetylglutamate kinase	1
283	tr|A0A369K927|A0A369K927_HYPMA	Acetylglutamate kinase	1
284	tr|A0A369K6X7|A0A369K6X7_HYPMA	ADP,ATP carrier protein	0
285	tr|A0A369K6L6|A0A369K6L6_HYPMA	40S ribosomal protein S11-B	0
286	RRRRRtr|K5XN05|K5XN05_AGABU	REVERSED Bromo domain-containing protein	0
287	tr|A0A369JFM9|A0A369JFM9_HYPMA	Heat shock protein 16	1
288	RRRRRtr|K5Y4R1|K5Y4R1_AGABU	REVERSED Adenylyl cyclase-associated protein	0
289	tr|A0A369JVP3|A0A369JVP3_HYPMA	Single-stranded TG1-3 DNA-binding protein	0
290	tr|A0A369K7E1|A0A369K7E1_HYPMA	Protein phosphatase PP2A regulatory subunit A	0
291	tr|A0A369JDX4|A0A369JDX4_HYPMA	60S ribosomal protein L8	0
292	tr|A0A369K9L8|A0A369K9L8_HYPMA	T-complex protein 1 subunit gamma	0
293	RRRRRtr|A0A369K698|A0A369K698_HYPMA	REVERSED Tyrosine-protein phosphatase yvh1	0
294	tr|A0A369J9E5|A0A369J9E5_HYPMA	ATP synthase subunit 4, mitochondrial	0
295	tr|K5Y7X4|K5Y7X4_AGABU	Histone H3	0
296	tr|K5Y245|K5Y245_AGABU	Histone H3	0
297	tr|K5W5A9|K5W5A9_AGABU	Histone H3	0
298	tr|A0A369JT59|A0A369JT59_HYPMA	Histone H3.2	0

299	tr|A0A151W1V5|A0A151W1V5_HYPMA	Histone H3	0
300	tr|A0A151VET2|A0A151VET2_HYPMA	Histone H3	0
301	tr|A0A369IYB3|A0A369IYB3_HYPMA	Eukaryotic translation initiation factor 3 subunit I	0
302	tr|A0A369JKP4|A0A369JKP4_HYPMA	Pyridoxal 5'-phosphate synthase (glutamine hydrolyzing)	0
303	tr|A0A369JLG1|A0A369JLG1_HYPMA	Small COPII coat GTPase SAR1	0
304	RRRRRtr|A0A369J836|A0A369J836_HYPMA	REVERSED RNase H domain-containing protein	0
305	tr|K5X203|K5X203_AGABU	Importin N-terminal domain-containing protein	0
306	RRRRRtr|A0A369JS84|A0A369JS84_HYPMA	REVERSED Beta-glucosidase	0
307	tr|A0A369J4Z3|A0A369J4Z3_HYPMA	Proteasome subunit alpha type	0
308	tr|A0A369K0J9|A0A369K0J9_HYPMA	Apoptosis-inducing factor 1	0
309	tr|A0A369JVD1|A0A369JVD1_HYPMA	Subtilisin-like serine protease pepC	0
310	tr|A0A369JKD9|A0A369JKD9_HYPMA	Pyruvate carboxylase	0
311	tr|A0A369JVF3|A0A369JVF3_HYPMA	T-complex protein 1 subunit epsilon	0
312	RRRRRtr|K5VJE8|K5VJE8_AGABU	REVERSED R3H domain-containing protein (Fragment) PE=3 SV=1	0
313	tr|K5XVG2|K5XVG2_AGABU	Thioredoxin reductase1	0
314	tr|A0A369JC73|A0A369JC73_HYPMA	Thioredoxin reductase	0
315	tr|K5WYB4|K5WYB4_AGABU	Serine hydroxymethyltransferase	0
316	tr|K5X115|K5X115_AGABU	Coronin	0
317	tr|A0A369K7J9|A0A369K7J9_HYPMA	Coronin	0
318	tr|A0A369JL80|A0A369JL80_HYPMA	54S ribosomal protein L23, mitochondrial	0
319	tr|A0A369K6J6|A0A369K6J6_HYPMA	RNA-binding protein rnc1	1
320	tr|A0A369JAL5|A0A369JAL5_HYPMA	Dihydrolipoyl dehydrogenase	0
321	RRRRRtr|A0A369JMU4|A0A369JMU4_HYPMA	REVERSED dUTP diphosphatase	0
322	RRRRRtr|A0A1B2U6U0|A0A1B2U6U0_FLAVE	REVERSED Glucoamylase	0
323	tr|A0A369K1H8|A0A369K1H8_HYPMA	Aldehyde dehydrogenase	2
324	tr|K5XK87|K5XK87_AGABU	Fumarate hydratase	0
325	tr|K5Y6R8|K5Y6R8_AGABU	Clp R domain-containing protein	1
326	tr|A0A369JBY3|A0A369JBY3_HYPMA	Heat shock protein, mitochondrial	0
327	tr|A0A1B2U6U0|A0A1B2U6U0_FLAVE	Glucoamylase	0
328	tr|K5XME7|K5XME7_AGABU	Mediator of RNA polymerase II transcription subunit 13 GN=AGABI1DRAFT_131978 PE=3 SV=1	0
329	tr|A0A369K9K8|A0A369K9K8_HYPMA	D-arabinitol dehydrogenase 1	1
330	tr|K5XI96|K5XI96_AGABU	CCT-alpha	0
331	tr|A0A369JQD1|A0A369JQD1_HYPMA	CCT-alpha	0
332	tr|A0A369JWQ5|A0A369JWQ5_HYPMA	Endoglucanase C	0
333	tr|K5XYH1|K5XYH1_AGABU	Tr-type G domain-containing protein	0
334	tr|A0A369JGN9|A0A369JGN9_HYPMA	Cytoskeleton-associated protein 5	0

335	tr|G8FUB2|G8FUB2_HYPMA	Glyceraldehyde-3-phosphate dehydrogenase	3
336	tr|G8FUB1|G8FUB1_HYPMA	Glyceraldehyde-3-phosphate dehydrogenase	3
337	tr|A0A369JU01|A0A369JU01_HYPMA	Glyceraldehyde-3-phosphate dehydrogenase	2
338	tr|S0BBZ0|S0BBZ0_HYPMA	Glyceraldehyde-3-phosphate dehydrogenase (Fragment)	1
339	tr|A0A369JKP7|A0A369JKP7_HYPMA	Non-specific serine/threonine protein kinase	0
340	tr|A0A369JTI9|A0A369JTI9_HYPMA	Eukaryotic translation initiation factor 5A	1
341	tr|K5Y4C5|K5Y4C5_AGABU	H(+)-transporting two-sector ATPase	0
342	tr|K5X600|K5X600_AGABU	Obg-like ATPase 1	0
343	tr|A0A369J6N9|A0A369J6N9_HYPMA	Kinesin light chain	1
344	RRRRRtr|A0A369JZ98|A0A369JZ98_HYPMA	REVERSED Nucleoporin Nup37	0
345	tr|K5XHW6|K5XHW6_AGABU	HATPase_c domain-containing protein	4
346	tr|A0A060ILH1|A0A060ILH1_AGABI	Heat-shock protein 90	4
347	tr|U5QEH9|U5QEH9_AGABI	Heat shock protein 90	4
348	tr|K5XU94|K5XU94_AGABU	Eukaryotic translation initiation factor 6	0
349	tr|A0A369J1M2|A0A369J1M2_HYPMA	Ubiquitin-conjugating enzyme spm2	0
350	tr|K5WAB1|K5WAB1_AGABU	Elongation factor Tu	0
351	tr|A0A369K5C3|A0A369K5C3_HYPMA	Elongation factor Tu	0
352	tr|A0A369JTG5|A0A369JTG5_HYPMA	Nitronate monooxygenase	0
353	tr|A0A369JD66|A0A369JD66_HYPMA	Putative methylcrotonoyl-CoA carboxylase beta chain, mitochondrial	0
354	RRRRRtr|A0A369J7I6|A0A369J7I6_HYPMA	REVERSED Cell division control protein 25	0
355	RRRRRtr|A0A369JEA9|A0A369JEA9_HYPMA	REVERSED DDE-1 domain-containing protein	0
356	tr|A0A369K2A2|A0A369K2A2_HYPMA	Protein-lysine methyltransferase METTL21D	0
357	RRRRRtr|K5X7J6|K5X7J6_AGABU	REVERSED V-type proton ATPase subunit	0
358	tr|A0A369JHP5|A0A369JHP5_HYPMA	Tripeptidyl-peptidase sed2	0
359	tr|A0A369JK75|A0A369JK75_HYPMA	5-methyltetrahydropteroyltriglutamate--homocysteine S-methyltransferase	9
360	tr|A0A369JP03|A0A369JP03_HYPMA	U6 snRNA-associated Sm-like protein LSm7	0
361	tr|K5XJS8|K5XJS8_AGABU	Acetyltransferase component of pyruvate dehydrogenase complex	1
362	tr|K5WXA7|K5WXA7_AGABU	DIS3-like exonuclease 2	0
363	tr|A0A369JKA6|A0A369JKA6_HYPMA	DIS3-like exonuclease 2	0
364	tr|A0A369JGJ3|A0A369JGJ3_HYPMA	Seryl-tRNA synthetase	0
365	tr|A0A369J780|A0A369J780_HYPMA	RPN13_C domain-containing protein	0
366	RRRRRtr|A0A369JI71|A0A369JI71_HYPMA	REVERSED Protein mms22	0

367	RRRRRtr|K5XHG2|K5XHG2_AGABU	REVERSED Nucleolar complex-associated protein 3	0
368	tr|K5XI07|K5XI07_AGABU	eRF1_1 domain-containing protein	0
369	tr|A0A369JP65|A0A369JP65_HYPMA	Eukaryotic peptide chain release factor subunit 1	0
370	tr|K5VR22|K5VR22_AGABU	60S ribosomal protein L20	0
371	tr|A0A369K1U6|A0A369K1U6_HYPMA	60S ribosomal protein L20-B	0
372	tr|A0A369JI85|A0A369JI85_HYPMA	Epidermal growth factor receptor substrate 15-like 1	0
373	tr|A0A369JDK2|A0A369JDK2_HYPMA	Ras-related protein Rab-11B	0
374	tr|K5XCR6|K5XCR6_AGABU	U6 snRNA-associated Sm-like protein LSm2	0
375	tr|A0A369JA19|A0A369JA19_HYPMA	Putative pyruvate decarboxylase C13A11.06	0
376	tr|K5XY53|K5XY53_AGABU	40S ribosomal protein S7	1
377	RRRRRtr|K5XVE0|K5XVE0_AGABU	REVERSED APH domain-containing protein	0
378	RRRRRtr|K5X9G7|K5X9G7_AGABU	REVERSED F-box domain-containing protein SV=1	0
379	RRRRRtr|K5XXB8|K5XXB8_AGABU	REVERSED 14_3_3 domain-containing protein SV=1	0
380	RRRRRtr|A0A369JCW3|A0A369JCW3_HYPMA	REVERSED 14-3-3 protein	0
381	tr|A0A369KE53|A0A369KE53_HYPMA	60S ribosomal protein L16	0
382	tr|A0A369JX19|A0A369JX19_HYPMA	Charged multivesicular body protein 2a 2	0
383	tr|A0A369K4Z3|A0A369K4Z3_HYPMA	U6 snRNA-associated Sm-like protein LSm6	0
384	tr|K5WQ26|K5WQ26_AGABU	Sm domain-containing protein	0
385	tr|A0A369JKE3|A0A369JKE3_HYPMA	Lactose permease	0
386	tr|A0A369JUC6|A0A369JUC6_HYPMA	40S ribosomal protein S4	0
387	tr|K5WX16|K5WX16_AGABU	60S ribosomal export protein NMD3	0
388	tr|A0A369K0H0|A0A369K0H0_HYPMA	60S ribosomal export protein NMD3	0
389	RRRRRtr|A0A369J6C0|A0A369J6C0_HYPMA	REVERSED Baeyer-Villiger monooxygenase	0
390	tr|A0A369J828|A0A369J828_HYPMA	Alpha-1,4 glucan phosphorylase	0
391	tr|A0A369K2W0|A0A369K2W0_HYPMA	Pre-mRNA-processing factor 19	0
392	RRRRRtr|Q8J1I8|Q8J1I8_AGABI	REVERSED Putative serine-threonine protein kinase (Fragment)	0
393	tr|A0A369K2X1|A0A369K2X1_HYPMA	60S ribosomal protein L13	0
394	tr|A0A369JNP1|A0A369JNP1_HYPMA	1-(5-phosphoribosyl)-5-[(5-phosphoribosylamino)methylideneamino] imidazole-4-carboxamide isomerase	0
395	tr|A0A369K6L4|A0A369K6L4_HYPMA	40S ribosomal protein S29	0
396	tr|K5X942|K5X942_AGABU	DUF2235 domain-containing protein 1	0
397	tr|A0A369JPF0|A0A369JPF0_HYPMA	DNA repair protein crb2	0
398	tr|A0A369JI16|A0A369JI16_HYPMA	Serine/threonine-protein kinase ssn3	0

399	tr|K5WZI1|K5WZI1_AGABU	Endoplasmic reticulum chaperone BiP	4
400	tr|K5W7M9|K5W7M9_AGABU	Proteasome subunit alpha type	1
401	tr|A0A369JI83|A0A369JI83_HYPMA	ATP-dependent RNA helicase FAL1	0
402	tr|A0A369JX12|A0A369JX12_HYPMA	Ubiquitin-like modifier HUB1	0
403	tr|A0A369K9H4|A0A369K9H4_HYPMA	Nucleosome assembly protein 1	0
404	tr|A0A369JZK5|A0A369JZK5_HYPMA	Nucleoside diphosphate kinase	0
405	tr|A0A369JYX3|A0A369JYX3_HYPMA	MYND-type domain-containing protein	0
406	tr|A0A369J400|A0A369J400_HYPMA	Apoptosis inhibitor 5 OS=Hypsizygus marmoreus	0
407	tr|A0A369JM63|A0A369JM63_HYPMA	60S ribosomal protein L3 OS=Hypsizygus marmoreus	0
408	RRRRRtr|A0A369J929|A0A369J929_HYPMA	REVERSED Non-specific serine/threonine protein kinase	0
